# High-throughput identification and quantification of single bacterial cells in the microbiota

**DOI:** 10.1038/s41467-022-28426-1

**Published:** 2022-02-22

**Authors:** Jianshi Jin, Reiko Yamamoto, Tadashi Takeuchi, Guangwei Cui, Eiji Miyauchi, Nozomi Hojo, Koichi Ikuta, Hiroshi Ohno, Katsuyuki Shiroguchi

**Affiliations:** 1 Laboratory for Prediction of Cell Systems Dynamics, RIKEN Center for Biosystems Dynamics Research (BDR), 6-2-3, Furuedai, Suita, Osaka 565-0874 Japan; 2 Laboratory for Intestinal Ecosystem, RIKEN Center for Integrative Medical Sciences (IMS), 1-7-22, Suehiro-cho, Tsurumi-ku, Yokohama, Kanagawa 230-0045 Japan; 3 Department of Microbiology and Immunology, Keio University School of Medicine, 35 Shinanomachi, Shinjuku-ku, Tokyo 160-8582 Japan; 4 Laboratory of Immune Regulation, Department of Virus Research, Institute for Frontier Life and Medical Sciences, Kyoto University, 53 Shogoin Kawahara-cho, Sakyo-ku, Kyoto 606-8507 Japan; 5 Intestinal Microbiota Project, Kanagawa Institute of Industrial Science and Technology, 3-2-1, Sakado, Takatsu-ku, Kawasaki, Kanagawa 213-0012 Japan; 6 Graduate School of Medical Life Science, Yokohama City University, 1-7-29, Suehiro-cho, Tsurumi-ku, Yokohama, Kanagawa 230-0045 Japan

**Keywords:** Metagenomics, Next-generation sequencing, Bacterial techniques and applications, Microbiome, Microbiology techniques

## Abstract

The bacterial microbiota works as a community that consists of many individual organisms, i.e., cells. To fully understand the function of bacterial microbiota, individual cells must be identified; however, it is difficult with current techniques. Here, we develop a method, Barcoding Bacteria for Identification and Quantification (BarBIQ), which classifies single bacterial cells into taxa–named herein cell-based operational taxonomy units (cOTUs)–based on cellularly barcoded 16S rRNA sequences with single-base accuracy, and quantifies the cell number for each cOTU in the microbiota in a high-throughput manner. We apply BarBIQ to murine cecal microbiotas and quantify in total 3.4 × 10^5^ bacterial cells containing 810 cOTUs. Interestingly, we find location-dependent global differences in the cecal microbiota depending on the dietary vitamin A deficiency, and more differentially abundant cOTUs at the proximal location than the distal location. Importantly, these location differences are not clearly shown by conventional 16S rRNA gene-amplicon sequencing methods, which quantify the 16S rRNA genes, not the cells. Thus, BarBIQ enables microbiota characterization with the identification and quantification of individual constituent bacteria, which is a cornerstone for microbiota studies.

## Abstract (web summary)

Here, Jin *et al*., develop a method called Barcoding Bacteria for Identification and Quantification (BarBIQ), which allows to both characterize the global microbiome and to identify and quantify single-cell bacterial members in a high-throughput manner.

## Introduction

Bacteria widely exist on Earth as communities^1^ that consist of a vast number of bacteria from a large number of bacterial species^2^, and they often live symbiotically with other organisms. These commensal bacterial communities, or microbiotas, are associated with the homeostasis of their hosts^3^. To understand how the bacterial microbiota affects the respective host homeostasis, two fundamental questions should be addressed: what kinds of bacteria (i.e., taxa) and how many organisms of each taxon are in the microbiota^4,5^. This compositional analysis is a basis for further understanding the mechanism of microbiota-host interactions and may be directly integrated with other analyses, such as metabolome analysis and spatial analysis^6–11^. However, measuring the microbiota composition at the organism (single cell) level has been difficult with the current techniques (described below), such as shotgun- and 16S rRNA gene-amplicon-based metagenomics^12–14^. Therefore, a cell-based high-throughput quantitative method is needed.

High-throughput methods based on 16S rRNA gene-amplicon sequencing using next-generation sequencing technology, including absolute quantification^4,15^, accurate sequencing^16–19^, full gene sequencing^20,21^, and bacteria-bacteria interactions^22^, have contributed to understanding bacterial diversity in microbiota for years^23,24^. However, because these conventional methods amplify 16S rRNA genes from purified bulk bacterial genomes and measure the number of amplified molecules, these methods basically have the following limitations for cell-based high-throughput quantification: (i) it is difficult to measure and compare the number of bacterial cells because different bacteria have distinct copy numbers of 16S rRNA genes in their genome (from 1 to 15 copies)^12,13,25^ and the copy numbers for many bacteria are unknown^26^; (ii) there are bacteria that have multiple 16S rRNA sequence types in their genome, and it is difficult to identify whether detected distinct 16S rRNA sequences are from the same cell^21,27^; and (iii) although recently developed methods, such as DADA2^19^, have corrected sequencing errors in the conventional methods, amplification errors, which are mainly from chimera generation (up to ~70%), cannot yet be effectively removed^28^. Without removing these errors, it is difficult to confirm the existence of newly identified 16S rRNA sequences from unknown bacteria.

**Fig. 1:**
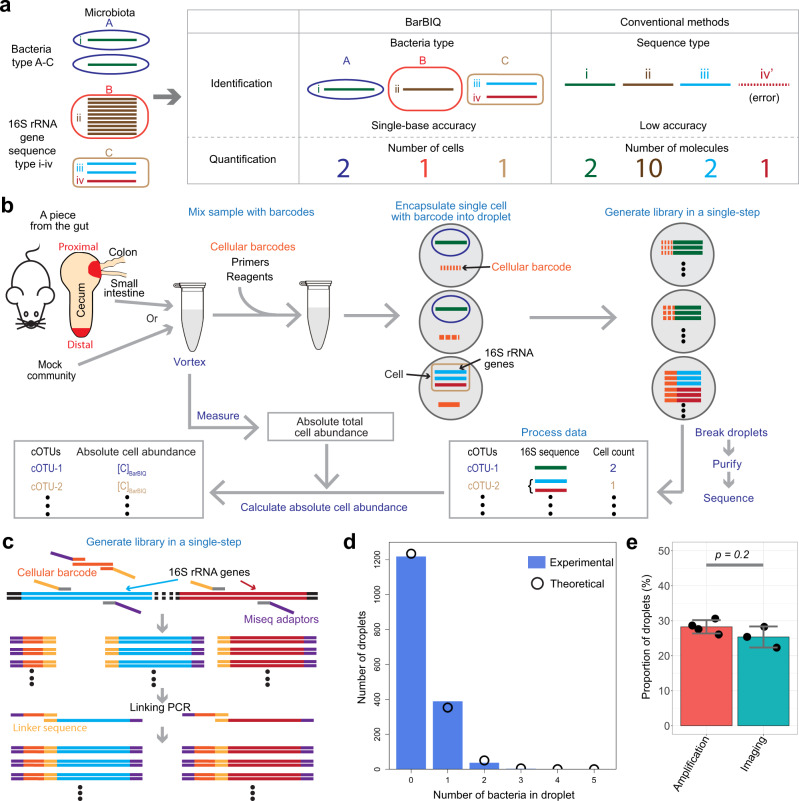
BarBIQ and its quality controls. **a** Main concept of BarBIQ and its comparison with conventional 16S rRNA gene-amplicon sequencing methods. **b** Schematic of BarBIQ. After the sample was suspended in a solution, vortexing was performed to break the clumps of bacteria. Cellular barcodes, DNA molecules containing random bases and primed sites for amplification; primers, DNA primers for amplification of both 16S rRNA genes and cellular barcodes, for linking both amplified products, and for attaching sequencing adapters; reagents, reagents for DNA amplification. Details for the schematics for library generation, purification, and sequencing are shown in Supplementary Fig. 4, and details for the data processing are shown in Supplementary Fig. 5. **c** Schematic of the library generation in a droplet. Both a cellular barcode and 16S rRNA genes (V3–V4 region, ~450 bases) in a bacterial genome in a drop were initially amplified by primers containing sequencing adapters and a linker sequence. Subsequently, the amplified barcodes were linked^36^ with the amplified 16S rRNA genes via the linker sequence. The DNA length is not to scale. **d** Comparison between the distribution of the number of bacteria in droplets (bars) observed by microscopic imaging (Supplementary Fig. 2) and the theoretical distribution (dots) calculated based on Poisson distribution. **e** Comparison between the proportion of droplets in which the 16S rRNA gene(s) in bacteria were amplified by ddPCR (Supplementary Note 1) and the proportion of droplets in which bacteria were observed by microscopic imaging (Supplementary Fig. 2); the droplets for both experiments were generated with the same cecal sample and with the same dilution factor. Data are presented as mean values ± SD (*n* = 4 for amplification, *n* = 3 for imaging). *P* value was calculated by the Kruskal-Wallis rank-sum test. Source data for (**d**) and (**e**) are provided as a Source Data file.

To overcome these limitations in conventional methods, we developed a method that identified and quantified individual bacteria in microbiota in a high-throughput manner by cellularly barcoded 16S rRNA sequencing with single-base accuracy, which is named Barcoding Bacteria for Identification and Quantification (BarBIQ) (Fig. 1a–c, Methods section, Supplementary Note 1 and 2). This method clarified both the global microbiota and individual bacterial members. We applied BarBIQ to murine cecal microbiota and found that the effect of dietary vitamin A deficiency on the microbiota at the proximal location (close to both joints of the colon-cecum and small intestine-cecum, Fig. 1b) of the cecum was larger than that at the distal location. Importantly, this was not clearly shown by the two conventional methods we conducted.

## Results

### BarBIQ, a high-throughput cell-based method

In BarBIQ (Fig. 1b), we first prepared a bacterial sample (mock community and murine cecal content; see below) in a buffer and broke the bacterial clumps by vortexing (Supplementary Fig. 1 and Supplementary Note 3). Next, we mixed the bacterial sample with a solution including cellular barcodes^29–32^, primers, and reagents for DNA amplification and then encapsulated them in droplets (~120 µm in diameter) using the Bio-Rad Droplet Digital^TM^ PCR (ddPCR) system. We adjusted the concentrations of barcodes and bacteria and their ratio based on the Poisson distribution. For barcodes, ~93% of the barcode occupied droplets each had a single barcode; multiple barcodes in the remaining 7% droplets did not significantly affect the measurement since the quantification of bacteria was on average changed by the factor 1.07 (=0.93 × 1 + 0.07 × 2). Moreover, this factor is common for all bacteria so that their relative proportions were not affected. We found that the bacteria did not affect barcode (83-base single-strand DNA) encapsulation (Supplementary Fig. 3a), which should have followed a Poisson distribution^33,34^. For bacteria, ~90% of the bacterial occupied droplets each had a single cell; multiple cells in the remaining 10% droplets were mostly different bacteria under this condition (Supplementary Note 1), and they can be distinguished by their 16S rRNA sequences (Supplementary Note 2). We confirmed that the cecal sample bacteria were successfully encapsulated into the droplets following Poisson distribution by microscopic imaging of individual bacteria (Fig. 1d and Supplementary Fig. 2). For subsequent sequencing, bacterial cell lysis by heating^35^, amplifications of barcode and 16S rRNA genes (V3–V4 region, ~450 bases), attachment of sequencing adapters, and linkage^36^ between amplified barcodes and 16S rRNA genes were performed in a single step (Fig. 1c and Supplementary Fig. 4). This process mostly avoids the generation of chimeras from different bacteria, which were largely generated by bulk PCR in 16S rRNA gene-amplicon sequencing^28^. We found that the proportion of droplets in which bacteria were detected by microscopic imaging was close to the proportion of droplets in which 16S rRNA genes were amplified for a cecal sample (Fig. 1e). We also confirmed that the amplification of 16S rRNA genes, including the cell lysis process, was robust by adjusting the initial heating time for cell lysis (Supplementary Fig. 3b). After amplification, we disrupted the droplets, purified the library (the linked amplicons), and sequenced both cellular barcodes and 16S rRNA sequences for individual amplified molecules using a high-throughput MiSeq sequencer. We analyzed the sequenced molecules (i.e., reads) for each sequence type of the barcodes (i.e., cell), identified 16S rRNA sequence(s) (termed Bar sequence(s)) for each cell, and counted the number of cells for each type of 16S rRNA sequence (Methods section, Supplementary Fig. 5, and Supplementary Note 2). More than 10^5^ cells were determined in a MiSeq run. Notably, this analysis also worked for a bacterium that had multiple 16S rRNA sequence types in its genome^27^ because the same cellular barcode was attached to the multiple amplified 16S rRNA sequences from the same cell, and these same bacterial 16S rRNA sequences were distinguished from incidentally coexisting 16S rRNA sequences, which were from different bacteria in a droplet, based on the quantification of cooccurrence and a statistical model (Supplementary Note 2, step 15). We finally obtained the absolute cell number per unit weight or volume for each 16S rRNA sequence type(s) (termed cOTU, explained below) in the sample by normalizing the sequencing-determined cell number using the total cell number per unit weight or volume of the same sample measured by ddPCR (Supplementary Note 1).

An essential difference between BarBIQ and conventional methods is the unit used for defining the composition of the microbiota and the quantification with the unit (Fig. 1a). In conventional methods, one of the commonly used units is the operational taxonomic unit (OTU)^37,38^, which represents a group of similar 16S rRNA sequences that are obtained by clustering based on the identities of sequences detected from a bulk sample^23,24^. Another widely used unit in conventional methods is amplicon sequence variant (ASV)^19,39^ which is used for a detected unique 16S rRNA sequence in the sample. OTUs and ASVs do not always represent bacterial cells because there are bacteria that have multiple 16S rRNA sequence types in their genome, as described above. However, BarBIQ identifies 16S rRNA sequence(s) (i.e., Bar sequence(s)) from each barcoded cell. To distinguish our cell-based method from conventional methods using OTUs, we named the 16S rRNA sequence(s) (i.e., Bar sequence(s)) from the same cell ‘cell-based operational taxonomy unit (cOTU)’. Furthermore, BarBIQ quantifies the number of cells for each cOTU, while conventional methods measure the number of amplified 16S rRNA gene molecules (16S rRNA gene abundance)^12,13^.

### Efficacy of BarBIQ performed using human gut bacterial strains

**Fig. 2:**
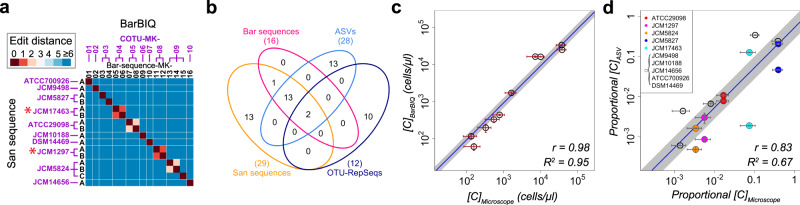
Efficacy of BarBIQ for the mock community and comparison with the conventional methods. **a** Comparison of the 16S rRNA sequences identified by Sanger sequencing and by BarBIQ. Edit distance, Levenshtein distance^68^, defined as the minimum number of substitutions, insertions, and deletions; San sequence, Sanger sequencing-identified 16S rRNA sequence; ATCC/JCM/DSM <number>, strain ID; A, B, or C, San sequences for each strain; Bar-sequence-MK-<number>, BarBIQ-identified sequences (Bar sequences); COTU-MK-<number>, cell-based operational taxonomy units (cOTUs); red star, Bar sequences that had one base difference. Source data are provided as a Source Data file. **b** Comparison (Venn diagram) among San sequences identified from each of the ten strains, Bar sequences, amplicon sequence variants (ASVs), and the representative sequences of operational taxonomy units (OTU-RepSeqs) identified from the mock community. Circle, total sequences from each method; numbers in circles, number of unique or identical sequences detected by the given method(s); numbers in the parentheses, total number of sequences detected by the given method. **c** Comparison of the absolute cell abundance per unit volume of 10 strains in the mock community measured by BarBIQ (*[C]*_*BarBIQ*_) (Supplementary Data 6) and by microscopic imaging (*[C]*_*Microscope*_) (Supplementary Table 1). Data are presented as mean values ± SD (*n* = 3 for *[C]*_*BarBIQ*_, *n* = 5 for *[C]*_*Microscope*_). Blue thin line, a fitting line with a fixed slope of one in log scale (intercept: -0.035) by considering the standard errors of both *[C]*_*BarBIQ*_ and *[C]*_*Microscope*_, indicating the averaged ratio *[C]*_*BarBIQ*_*/[C]*_*Microscope*_ as 0.92; ﻿ gray thick line, 95% confidence interval of the fitted line, indicating the 95% confidence interval of the ratio *[C]*_*BarBIQ*_*/[C]*_*Microscope*_ as 0.68~1.25; *r*, Pearson coefficient; *R*^*2*^, coefficient of determination. **d** Comparison of the proportional abundances of 15 ASVs in the mock community measured by Conv_ASV (proportional *[C]*_*ASV*_) (Supplementary Data 5) with the proportional *[C]*_*Microscope*_ measured by microscopic imaging. Data are presented as mean values ± SD (*n* = 2 for proportional *[C]*_*ASV*_, *n* = 5 for proportional *[C]*_*Microscope*_). Strains that had commonly detected sequence(s) are shown. The strains that were compared with multiple identical ASVs are shown in colors. By the same fitting as **c** (intercept: −0.28), the averaged ratio of proportional *[C]*_*ASV*_ and proportional *[C]*_*Microscope*_ was 0.52, and its 95% confidence interval was 0.28–0.99.

We demonstrated that BarBIQ robustly worked for a mock community that contained designed abundances of 10 cultured human gut bacterial strains (Methods section, Supplementary Table 1); these strains have been used as a model community that represented the four most prominent bacterial phyla (*Actinobacteria* (1 strain), *Bacteroidetes* (3 strains), *Firmicutes* (4 strains), and *Proteobacteria* (2 strains)) in the healthy human gut microbiota^40^ and included both Gram-positive (5 strains) and Gram-negative (5 strains) bacteria. We found 16 Bar sequences from the mock community (Fig. 2a and Supplementary Data 1). These identified Bar sequences were completely consistent among the three technical replicates (Supplementary Data 1). All 16 Bar sequences were identical to one of the Sanger sequencing-identified 16S rRNA sequences (San sequences) that we identified from each of the 10 cultured strains (Fig. 2a, b, Methods section, Supplementary Data 2). On the other hand, there were another 13 San sequences that had one or two substitutions from the 16 sequences above (San-Bar matched sequences) (Fig. 2b and Supplementary Data 2). We found that these 13 San sequences were reasonably explained by PCR errors in the amplification for Sanger sequencing with the error rate of the polymerase we used (Supplementary Fig. 6a), suggesting that the San sequences and Bar sequences were completely consistent. We then identified 10 cOTUs from the 16 Bar sequences based on the cellular barcodes (Methods section, Supplementary Note 2), and each corresponded to one of the 10 strains, which showed as well that two pairs of Bar sequences that each differed in one base and was from the same bacterial cell (Fig. 2a). Therefore, we concluded that BarBIQ identified the correct number of strains (cOTUs) in the mock community and had single-base accuracy and resolution for 16S rRNA sequence identification.

In comparison, we analyzed the same mock community by conventional methods with ASV-based analysis using DADA2^19^ and with OTU-based analysis using Mothur^41^ (Methods section) and identified 28 ASVs and 12 OTUs (Supplementary Data 3 and 4). The numbers of ASVs and OTUs were larger than the number of strains in the mock community, indicating that at least some of the ASVs and OTUs did not represent the strains. Furthermore, we compared the identity of ASVs and the representative sequences of OTUs (OTU-RepSeqs, Methods section) to both the San sequences from the ten strains and the Bar sequences from the mock community. For ASV, although 15 ASVs were identical to one of the San-Bar matched sequences, the other 13 ASVs were not identical to any San sequence (Fig. 2b). We found that the abundances of the 13 nonidentical ASVs varied more than the estimated sampling noise, while the 15 identical ASVs followed the estimated sampling noise well (Supplementary Fig. 6b), and the abundances of the 13 nonidentical ASVs were globally much different from that of the 15 identical ASVs (Supplementary Fig. 6b and Supplementary Data 5), suggesting that the 13 nonidentical ASVs were not correct 16S rRNA sequences from the ten strains. For OTUs, only two of 12 OTU-RepSeqs were identical to one of the San-Bar matched sequences from different strains, and the other 10 OTU-RepSeqs were not identical to any San sequence (Fig. 2b and Supplementary Fig. 6c), meaning that the OTU-based analysis did not detect the correct 16S rRNA sequences from at least eight strains in the 10-strain mock community. Importantly, the 13 San sequences that were not identical to any Bar sequence did not match any ASV or OTU-RepSeqs (Fig. 2b), which supported our interpretation that these 13 San sequences included amplification error(s). Thus, BarBIQ had the highest accuracy of the 16S rRNA sequence identification for the mock community in comparison with that of the conventional methods that we used here.

By BarBIQ, we then measured the cell abundance (number of cells) per unit volume of each cOTU (*[C]*_*BarBIQ*_) in the mock community (Supplementary Data 6) and compared *[C]*_*BarBIQ*_ with the cell abundance per unit volume in the mock community, which was calculated by the dilution factor from the measured abundance by microscopic imaging (*[C]*_*Microscope*_) for each strain (Fig. 2c, Methods section, Supplementary Fig. 7, and Supplementary Note 4). First, the reproducibility among three technical replications of *[C]*_*BarBIQ*_ was high (standard deviation/mean: 0.01~0.25, *N* = 3; Fig. 2c). Second, the Pearson product-moment correlation coefficient *r* (Pearson’s *r*) between *[C]*_*BarBIQ*_ and *[C]*_*Microscope*_ was 0.98, and the averaged ratio *[C]*_*BarBIQ*_*/[C]*_*Microscope*_ was 0.92 (95% confidence interval: 0.68–1.25; Fig. 2c). Thus, BarBIQ accurately measured the cell abundance of each bacterial strain in the mock community with high reproducibility.

We then compared the 16S rRNA gene abundance of the 15 San sequence-identical ASVs (*[C]*_*ASV*_) to the *[C]*_*Microscope*_ (the strains that had multiple 16S rRNA sequences were compared to multiple identical ASVs) with proportional normalization. As expected, the proportional *[C]*_*ASV*_ was not well correlated with the proportional *[C]*_*Microscope*_ (Fig. 2d). To understand this difference, we estimated the cell abundance from [*C*]_*ASV*_ ([*C*]_*ASV-estimated*_); for 6 of the 10 strains that have 16S rRNA gene copy numbers in their genome that are registered in the rrnDB database^42^, we summed the abundance of multiple ASVs that were identical to the San sequences from the same strain and calculated the proportional *[C]*_*ASV-estimated*_ from the *[C]*_*ASV*_ using their 16S rRNA gene copy numbers (Supplementary Fig. 8). The Pearson’s *r* between the proportional *[C]*_*ASV-estimated*_ and proportional *[C]*_*Microscope*_ (0.97) were increased from that between proportional *[C]*_*ASV*_ and proportional *[C]*_*Microscope*_ (0.91), which was consistent with the design principle of the conventional method that measured the number of gene copies but not cells.

### Measurement conditions for murine cecal microbiota

**Fig. 3:**
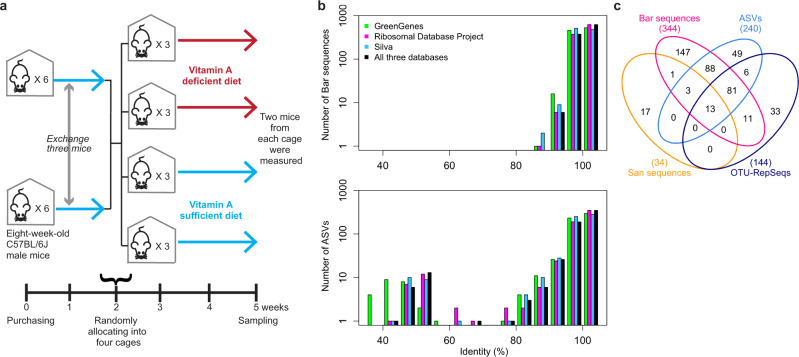
Dietary vitamin A experimental design and identified 16S rRNA sequences of the murine cecal microbiotas. **a** Schematic of the experimental design of the dietary vitamin A experiments (Methods). **b** Sequence identity profile of Bar sequences and ASVs; identity, the identity between each Bar sequence or ASV and its closest 16S rRNA sequence in three public databases: GreenGenes, Ribosomal Database Project, and Silva. Source data are provided in Supplementary Data 1 and 3. **c** Comparison (Venn diagram) among San sequences, Bar sequences, ASVs, and OTU-RepSeqs identified from the cell-sample at the proximal location of the mouse VDd. Numbers, same as Fig. 2b.

As an application of BarBIQ for biological samples, we surveyed the microbiota of the murine cecum at two locations (distal and proximal, Fig. 1b) in C57BL/6J male mice mainly depending on diets. The mice maintained under three conditions as follows were analyzed (Methods section): (i) three 6-week-old mice (CEa, CEb, and CEc) from CLEA Japan that was maintained by a balanced nutrient diet CE-2 for their full life span (CE2-nutrient group); (ii) four 8-week-old mice (VSa, VSb, VSc, and VSd) that were purchased from Japan SLC, Inc. and maintained for 5 more weeks by being fed with a compositionally well-defined nutrition-balanced diet (Supplementary Table 2) (VA-sufficient group) (Fig. 3a); and (iii) four mice (VDa, VDb, VDc, and VDd) that underwent the same as those in (ii) except that the vitamin A was not included in the diet (Supplementary Table 2) for the last 3 weeks (VA-deficient group) (Fig. 3a). Investigating the effect of dietary vitamin A on gut microbiota is important because bacteria are essential for vitamin A absorption and storage, and vitamin A deficiency affects the vision, growth, and immune function of the human body, which causes public health problems^43,44^. For the BarBIQ measurement, we first separated cells and extracellular DNA (ecDNA) from the cecal content of each location in each mouse by filtration, and then measured the filter-residue (cell-sample) and flow-through (ecDNA-sample) (Methods section, Supplementary Fig. 9, and Supplementary Note 5), because extracellular bacterial DNA may affect the quantification of the intestinal microbiota, as recently reported^45^. We note that this filtration procedure may be used for different types of samples, e.g., oral and skin samples, even if purification and/or concentration is required. We performed three technical replicates for both cell- and ecDNA-samples at each location in the mouse CEa and one measurement for all others; each measurement required <1 mg content.

### Accurate identification of the 16S rRNA sequences of cOTUs for murine ceca

In total for all cell-samples, we counted 3.4 × 10^5^ bacterial cells and identified 810 cOTUs containing 954 Bar sequences (Supplementary Data 1). In addition, we uniquely identified another 50 Bar sequences from the ecDNA-samples (Supplementary Data 1); we did not define these Bar sequences as cOTUs since the ecDNAs do not represent cells. Importantly, 383 identified Bar sequences (38% of the 1004 (954 + 50)) were not registered in three widely used public databases (GreenGenes^46^, Ribosomal Database Project^47^, and Silva^48^), and all Bar sequences had >93% identity to their closest 16S rRNA sequences in these three databases (Fig. 3b, Methods section, Supplementary Data 1). To confirm that the database-unregistered Bar sequences were accurate, we performed Sanger sequencing for the PCR amplicons of 16S rRNA genes from randomly selected single bacterial cells of the cell-sample from the proximal location of the mouse VDd (VDd^prox^) and identified 34 unique San sequences (Methods section, Supplementary Data 2). Seventeen (six were not registered in the databases) of the 34 were identical to one of the Bar sequences identified from the same sample VDd^prox^ (Fig. 3c); the remaining 17 San sequences had one, two, or three substitutions or one deletion from the Bar sequences, which was reasonably explained by simulated PCR errors for Sanger sequencing with the error rate of the polymerase we used (Supplementary Fig. 10a). These results suggested that the identified Bar sequences are accurate, which was also evidenced by the mock community experiment above.

In comparison, we also measured 16 cell-samples (4 mice × 2 locations × 2 diet conditions) from the VA-sufficient and VA-deficient groups (VA group) using the ASV-based and OTU-based analyses, respectively (Supplementary Data 3, 4, 5, and 7). To investigate the accuracy of sequence identification for both the ASV-based and OTU-based analyses here, we again used the results of cell-sample VDd^prox^ as above. For ASV, 16 San sequences were identical to one of the ASVs, and the other 18 nonidentical San sequences were reasonably explained by simulated PCR errors for Sanger sequencing (Fig. 3c and Supplementary Fig. 10b). However, for all 16 cell-samples, 4% of ASVs (21 ASVs) had a low (<70%) identity (7% ASVs had <93% identity) with the closest registered 16S rRNA sequence in the databases (Fig. 3b), while the identity between almost all pairs of registered 16S rRNA sequences in the databases was >70% (Supplementary Fig. 10c), suggesting that these low-identity ASVs were not 16S rRNA sequences but probably PCR-errored sequences, e.g., chimeras. For OTUs, although 13 San sequences were identical to one of the OTU-RepSeqs, the other 21 nonidentical San sequences had large differences from the OTU-RepSeqs, which cannot be explained by PCR errors for Sanger sequencing globally (Fig. 3c and Supplementary Fig. 10d), suggesting that the 16S rRNA sequence identification accuracy by the OTU-based analysis was not high. Importantly, the 17 San sequences that were not identical to any Bar sequence did not match any ASV or OTU-RepSeqs (Fig. 3c), which supported our interpretation that these 17 San sequences included amplification error(s). Collectively, for both the mock community and the murine cecal samples, BarBIQ showed the highest accuracy of 16S rRNA sequence identification, which enables BarBIQ to identify unknown 16S rRNA sequences without any predetermined sequences.

### Highly reproducible cell abundance quantification of cOTUs in murine ceca

**Fig. 4:**
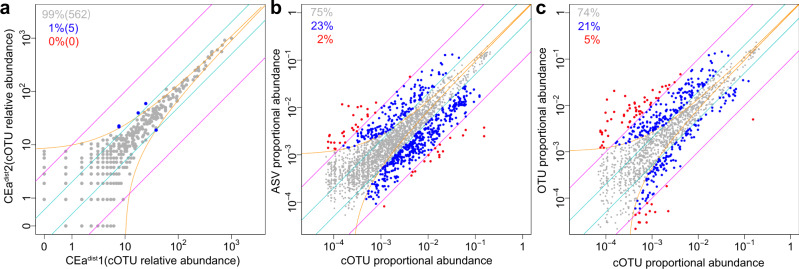
Highly reproducible quantification of BarBIQ and comparison with conventional methods. **a** Comparison (dots) of the relative abundances of cOTUs between a pair of technical replicates (other pairs in Supplementary Fig. 12). Orange lines, theoretical confidential interval (99.9%) for sampling noise based on the Poisson distribution and normalization to the relative abundance (to the proportional abundance for **b** and **c**); cyan lines, 2-fold change; magenta lines, 10-fold change; blue dots, cOTUs that showed larger difference than that of the sampling noise and 2-fold change and was smaller than the 10-fold change; proportions and numbers in the parentheses, proportions and the numbers of dots in corresponding colors, respectively; CEa, mouse; dist and prox, locations; 1 and 2, technical replicates (see text). **b** Comparison between the proportional abundances of the common pairs of cOTUs and ASVs with all 16 cell-samples shown in the same format as **a**. Red dots, common pairs of cOTUs and ASVs that showed larger difference than the sampling noise and 10-fold change. **c** Comparison between the proportional abundances of the common pairs of cOTUs and OTUs with all 16 cell-samples shown in the same format as **a** and **b**. Source data are provided as a Source Data file.

For each identified cOTU in each cell-sample, we quantified the absolute cell abundance per unit weight by BarBIQ (Supplementary Data 8) and calculated the relative cell abundance by normalization among samples using the median of ratios method^49^ (Methods section). We found that the total absolute cell abundances per unit weight were different among mice (maximum fold change: 2.6 (distal) and 3.2 (proximal)), and the abundance at the distal location was always higher (1.1–3.4 times) than that at the proximal location for all mice (Supplementary Fig. 11). For each cOTU, we confirmed that the results of both relative and absolute cell abundance were highly reproducible by technical replicates for the same sample (CEa^dist^ and CEa^prox^) (Fig. 4a and Supplementary Fig. 12a, b) and that the noise for quantification was mainly from sampling by simulation (Supplementary Fig. 12c–e).

To investigate the difference in the quantifications by BarBIQ with those by the ASV-based and OTU-based analyses, we compared the proportional cell abundances (divided by the total number of cells) of the cOTUs to the proportional 16S rRNA gene abundances of the ASVs and OTUs with the commonly detected 16S rRNA sequences in each of the 16 cell-samples in the VA group; the cOTU that contained multiple Bar sequences was compared with each corresponding ASV or OTU. As expected, the differences were large (Fig. 4b, c and Supplementary Fig. 13a, b): in all samples, between cOTUs and ASVs, 25% of the compared pairs were >2 times different, and 2% were >10 times; between cOTUs and OTUs, 26% were >2 times and 5% were >10 times. In addition, the 16S rRNA gene abundances of ASVs and OTUs for the commonly detected 16S rRNA sequences were also different (12% >2 times and 2% >10 times) (Supplementary Fig. 13a–c). To understand this difference, we considered the copy numbers of 16S rRNA genes in the genome as described for the mock community (Supplementary Fig. 14), which was again consistent with the design principle of these methods in which BarBIQ measures cell abundance and the ASV-based and OTU-based analyses measure 16S rRNA gene copies.

### cOTU-based alpha and beta diversities

**Fig. 5:**
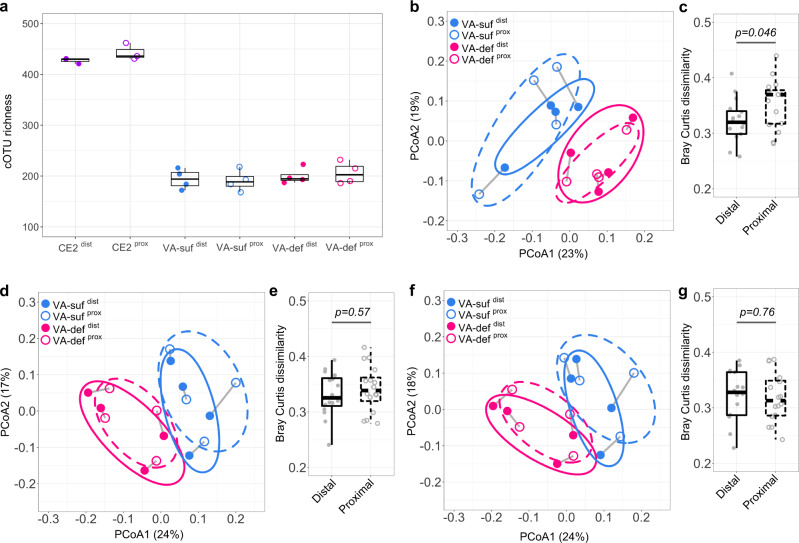
cOTU richness and Bray-Curtis dissimilarity of the murine cecal microbiotas. **a** The cOTU richness of each cell-sample determined by subsampling 6608 cells using the function rarefy in the R package Vegan. CE2, CE2 nutriment group; VA-suf, VA-sufficient group; VA-def, VA-deficient group; dist and prox, locations. **b**, **d**, and **f**, Principal coordinate analysis (PCoA) of Bray-Curtis dissimilarities calculated based on the relative cell abundances of cOTUs (**b**), ASVs (**d**), and OTUs (**f**) between each pair of cell-samples in the VA group. Labels, same as (**a**); gray line, linkage from the same mouse; circles, 95% confidence ellipses for each group. **c**, **e**, and **g**, Quantitative comparison of Bray-Curtis dissimilarities in **b**, **d**, and **f**, respectively. Distal, all possible pairs from VA-suf^dist^ and VA-def^dist^, respectively; Proximal, all possible pairs from VA-suf^prox^ and VA-def^prox^, respectively. Boxes in **a**, **c**, **e**, and **g** represent 25th to 75th percentiles (the interquartile range), horizontal black lines indicate medians, and whiskers show 1.5 times the interquartile range (*n* = 3 for CE2^dist^ and CE2^prox^; *n* = 4 for VA-suf^dist^, VA-suf^prox^, VA-def^dist^, and VA-def^prox^; *n* = 16 for Distal and Proximal). *P* values were calculated by the Kruskal–Wallis rank-sum test. Source data are provided as a Source Data file.

BarBIQ enabled both alpha and beta diversity analyses of the bacterial microbiota based on cells (organisms); defining alpha and beta diversity based on organisms is important for understanding the bacterial ecosystem, and this was proposed approximately a half century ago^50^. As an example of alpha diversities, we defined cOTU-based taxon richness (cOTU richness) as the number of observed cOTUs in a certain total number of detected cells from a sample; taxon richness is a common biodiversity assessment^51^. We found that the cOTU richness was very different (~2 times) between the CE2-nutrient and VA groups (Fig. 5a), which may be due to the different diets, maintaining facilities, or ages among the groups. On the other hand, the cOTU richness was consistent between the VA-sufficient and VA-deficient groups as well as between distal and proximal locations within each group (Fig. 5a).

As an example of beta diversities, we calculated Bray-Curtis dissimilarity (abundance-based beta diversity)^52^ based on the relative cell abundances of the detected cOTUs (Methods section), which quantified the global difference for each pair of samples, and performed principal coordinates analysis (PCoA) for the CE2-nutrient (Supplementary Fig. 15a) and VA groups (Fig. 5b). First, the distal and proximal location groups were not separated for the CE2-nutrient, VA-sufficient, and VA-deficient groups. In detail, the dissimilarities between samples from different mice at the distal or proximal location (magenta symbols in Supplementary Fig. 15b) were higher than those at different locations from the same mouse (blue symbols in Supplementary Fig. 15b), indicating that the mouse variances were globally larger than the location variances. Second, the VA-sufficient and VA-deficient groups were well separated for both the distal and proximal locations (Fig. 5b). Interestingly, the difference between the VA-sufficient and VA-deficient groups at the proximal location was significantly larger than those at the distal location (Fig. 5c). This phenomenon was also found by the Bray-Curtis dissimilarities calculated based on the absolute cell abundances of the cOTUs (Supplementary Fig. 15c). This location difference suggested that the microbiota at the proximal location of the murine cecum was globally affected more than that at the distal location by VA-deficient diet feeding for 3 weeks.

We also calculated the Bray-Curtis dissimilarities between cell-samples using the 16S rRNA gene abundance measured by the ASV-based and OTU-based analyses and performed PCoA for the VA group (Fig. 5d, f, Methods section). Smaller dissimilarities between the distal and proximal locations were found again within the same mice compared to that between mice at each location (Supplementary Fig. 15d, e). However, the dissimilarities based on 16S rRNA gene abundance between VA-sufficient and VA-deficient groups at the distal location were not significantly different from those at the proximal location, which was inconsistent with the cOTU-based BarBIQ results (Fig. 5e, g). These results suggested that biological conclusions may be tilted by the difference between 16S rRNA gene abundance and cell abundance.

### Differential cell abundance of cOTUs between cecal locations

**Fig. 6:**
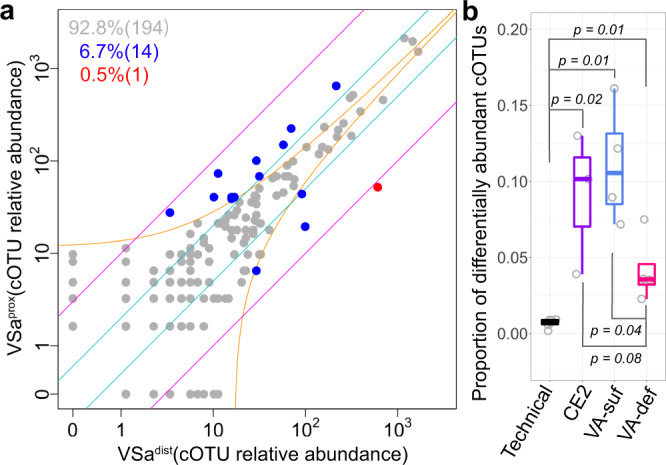
Comparison of location-dependent relative cell abundance in each mouse. **a** Comparison between the relative cell abundances of cOTUs detected from the distal location and proximal location of the mouse VSa, shown in Fig. 4a (other mice in Supplementary Fig. 16a). **b** Proportion of location-dependent differentially abundant cOTUs (differences were larger than the sampling noise and 2-fold) in each mouse. Technical, all pairs from three technical replicates within the distal and proximal locations for the mouse CEa; CE2, the mice in the CE2 nutriment group; VA-suf, the mice in the VA-sufficient group; VA-def, the mice in the VA-deficient group. Boxes represent 25th to 75th percentiles (the interquartile range), horizontal black lines indicate medians, and whiskers show 1.5 times the interquartile range (*n* = 6 for Technical, *n* = 3 for CE2, *n* = 4 for VA-suf and VA-def). *P* values were calculated by the Kruskal–Wallis rank-sum test. Source data are provided as a Source Data file.

We first compared the relative cell abundances of each cOTU between the distal and proximal locations in each mouse (one example in Fig. 6a; all in Supplementary Fig. 16a). The results showed that the proportions of differentially abundant cOTUs (differences were larger than the sampling noise and 2-fold) between the locations of the same mouse in the CE2-nutrient (4–13%; 22–75 cOTUs), VA-sufficient (7–16%; 15–43 cOTUs), and VA-deficient groups (2–8%; 5–18 cOTUs) were all significantly larger than those between technical replicates (0.2–0.9%; 1–5 cOTUs) and that the mice in the VA-deficient group had the lowest proportions of differentially abundant cOTUs (Fig. 6b). We also found similar results for the absolute cell abundances (Supplementary Fig. 16b, c). These results suggested that the bacterial compositions were slightly different between the two locations in each mouse, which was consistent with the Bray–Curtis dissimilarities above (Fig. 5b and Supplementary Fig. 15b). However, most of the differentially abundant cOTUs between locations in the same mouse were not consistent among mice (Supplementary Fig. 17), suggesting that each cOTU should be investigated to accurately characterize the microbiota.

### Differential cell abundance of cOTUs regarding dietary vitamin A deficiency

**Fig. 7:**
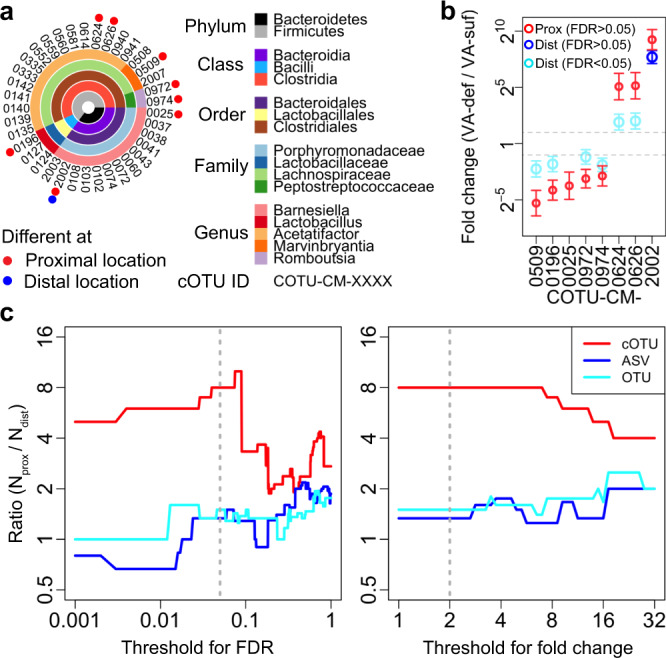
Differential cell abundance of cOTUs depending on dietary vitamin A deficiency. **a** The detected cOTUs and taxonomies (predicted by the RDP classifier, see Methods section). The genera that included at least one differentially abundant cOTU between the VA-sufficient and VA-deficient groups at either the distal or proximal location are shown (other in Supplementary Data 8). Different, FDR < 0.05 and fold change > 2. **b** The estimated fold changes (dots) and standard errors (error bars, *n* = 4) by DESeq2 between the VA-sufficient group (VA-suf) and VA-deficient group (VA-def) of the differentially abundant cOTUs (FDR < 0.05, fold change > 2) at either distal (Dist) or proximal (Prox) location. The differential analysis of COTU-CM-0025 at the distal location was not performed since its abundances were too low (Methods section). **c** The ratio between the number of differentially abundant cOTUs, ASVs, or OTUs at the proximal location (N_prox_) and at the distal location (N_dist_) as a function of threshold for FDR (left) and for fold change (right). FDR < 0.05 and a fold change > 2 (gray dotted lines) were considered “significant differences” in other analyses. Source data for (**b**) and (**c**) are provided as a Source Data file.

To investigate the effect of dietary vitamin A deficiency on each cOTU, we performed differential abundance analysis^53^ between the VA-sufficient and VA-deficient groups using the relative cell abundances of the cOTUs for the distal and proximal locations; 153 cOTUs for distal location and 150 cOTUs for proximal location were compared after removing low abundant noisy cOTUs (Methods section, Supplementary Fig. 18a, b). We found that eight cOTUs from five genera (*Acetatifactor, Barnesiella, Lactobacillus*, *Marvinbryantia*, and *Romboutsia*) and two phyla (predicted by the Bar sequence(s) for each cOTU; Methods section) were significantly different (false discovery rate (FDR) < 0.05 and fold change > 2) between the VA-sufficient and VA-deficient groups at the proximal location, while only one (cOTU-CM-2002) (of the eight above) showed a significant change at the distal location (Fig. 7a, b and Supplementary Fig. 18a, b). This result is consistent with a study showing that only a few strains were significantly increased or decreased when a colonized human gut-derived bacterial community (92 strains) in mice were fed a vitamin A-deficient diet for 3 weeks^43^. The maximum compositions of the eight cOTUs respectively among all 16 cell-samples in the VA-group were in the range of 0.4% (COTU-CM-0025) to 6.5% (cOTU-CM-2002) (Supplementary Data 8). The cOTU-CM-2002 exhibited the largest increase upon the vitamin A-deficient diet (fold change: 320–1100 (log2-fold change with standard error: 9.2 ± 0.9) at the proximal location and 140–320 (7.7 ± 0.6) at the distal location) (Fig. 7b and Supplementary Fig. 18a, b). This cOTU was from the genus *Barnesiella*; however, 11 of 13 analyzed cOTUs in the genus *Barnesiella* did not show a significant difference. Similarly, in the genera *Acetatifactor, Lactobacillus*, and *Marvinbryantia*, some cOTUs showed significant differences, but others did not (Fig. 7a). These results indicated that vitamin A deficiency shaped the microbiota at the cOTU level and that the cOTU level difference was larger at the proximal location than at the distal location.

We again compared BarBIQ with conventional methods in vitamin A diet experiments by performing differential abundance analysis between VA-sufficient and VA-deficient groups using the relative 16S rRNA gene abundances of the ASVs and OTUs, respectively, for each location (distal location: 223 ASVs and 131 OTUs were compared; proximal location: 236 ASVs and 138 OTUs) (Methods section; Supplementary Fig. 18c–f). The number of significantly different ASVs or OTUs (FDR < 0.05 and fold change > 2) between VA-sufficient and VA-deficient groups at the distal location (6 ASVs or 6 OTUs) was similar to that at the proximal location (8 ASVs or 9 OTUs), which was inconsistent with the results based on cOTUs. This discrepancy between cOTUs and ASVs or OTUs was robust even when we changed the thresholds of FDR and fold change, which defined the significance (Fig. 7c). These results again suggested that the inconsistency between the cell abundance and 16S rRNA gene abundance, which has been pointed out for years,^12,13,25^ is not negligible for understanding this biological phenomena. Importantly, the largest differentially abundant cOTU (cOTU-CM-2002) determined by BarBIQ (mentioned above) was determined as two separate units by both the ASV-based (ASV28 and ASV31) and OTU-based analyses (Otu029 and Otu032), which also indicated the essential difference between cell-based identification by BarBIQ and gene-based identification by conventional methods. In addition, the OTU-RepSeq of Otu032 had one base difference from ASV31, which is an example of an inconsistency between the ASV-based and OTU-based analyses. Notably, five of the six differentially abundant ASVs at distal location and seven of the eight ASVs at proximal locations were detected by BarBIQ, while four of the six OTUs at distal location and five of the nine OTUs at proximal locations were detected by BarBIQ (Supplementary Data 1, 3, and 4).

### Quantification of ecDNA in murine ceca

Although our main goal of ecDNA separation was to accurately measure cell abundance, the measurement of ecDNA may be useful for some studies, e.g., measuring the drug killing efficiency for specific bacteria by monitoring ecDNAs from dead cells. Here, we applied BarBIQ to DNA fragments containing 16S rRNA gene(s), which were segmented by vortexing, (Methods section, Supplementary Note 5) from the ecDNA-samples and detected 664 cOTUs that were determined by the cell-samples and uniquely identified 50 Bar sequences. For these cOTUs and Bar sequences, we quantified the number of DNA fragments (Supplementary Data 8). We found that the ratios of total DNA fragment abundances (Supplementary Fig. 19a) and total cell abundances (ecDNA/cell) per unit weight (Supplementary Fig. 11) for each location and mouse in the CE2-nutrient group (0.01–0.04) were much smaller than those in the VA group (0.08–1.3) (Supplementary Fig. 19b). We then compared the ecDNA/cell ratios for five cOTUs that were commonly detected in all cell- and ecDNA-samples. Interestingly, the ratios of cOTU-CM-0074 and cOTU-CM-2009 in the CE2-nutrient group were significantly smaller than those in the VA group, which was consistent with the tendency of the total ecDNA/cell ratios, while the ratios of cOTU-CM-0823 were comparable between the CE2-nutrient and VA groups (Supplementary Fig. 19c). The results showing the variance in cOTUs suggested that the distinct tendencies of the ratio between ecDNA and cells, which may represent the ratio between broken and nonbroken cells in different bacteria, might be related to bacteria-microenvironment interactions.

## Discussion

In this study, BarBIQ, a developed high-throughput and accurate method, defined microbiota at the single-cell level. By the cOTU-based analysis provided by BarBIQ, we discovered that dietary vitamin A deficiency effects the microbiota at both the proximal and distal locations of the murine cecum, and the effect at the proximal location was larger after 3 weeks of feeding. Based on the structure of the murine intestinal tract, this observed phenomenon suggested that the content within the cecum was not completely homogenized, and the dietary effect on the microbiota at the proximal location was partially transformed to the distal location in 3 weeks. Importantly, this phenomenon was not clearly identified by the ASV- or OTU-based conventional methods. Thus, BarBIQ accurately visualizes both the global microbiota and individual bacterial members, which is essentially different from conventional 16S rRNA gene-amplicon analyses and will enable an effective understanding of microbiota functions.

BarBIQ will continue to contribute to future studies, as it can provide a database with cOTUs, quantification of each cOTU, and newly identified 16S rRNA sequences in any microbiota community. Importantly, cOTUs may be directly linked to organisms with whole-genome sequence and/or functions. Furthermore, cOTU-based profiling can also successfully facilitate the progress of diverse microbiota research from current associative studies to the required mechanistic studies^6,7,11,54–57^ by quantitative integration of multiple meta-omics^6,7,9,11,55–57^, imaging, and/or computational modeling^7,9,11^ using a common unit, the cell. BarBIQ may thus be used in microbiota-related wide research fields such as those which focus on the gut^6^, oral^58^, skin^59^, marine^56^, soil^57^, plant^60^, and other terrestrial environments^55^ to clarify both the global microbiota and individual bacterial members as mentioned above, which will provide new insights, such as the examples shown in our studies, into biological phenomena. Moreover, BarBIQ can be further extended to the quantitative profiling of cancer cells, fungi, and viruses by designing target-specific primers. Indeed, high-throughput single-cell genomic sequencing, e.g., profiling cellular gene variations in a tumor, has been difficult. Thus, BarBIQ will be useful for microbiota- and high-throughput genomic sequencing-related wide studies.

## Methods

### Mouse preparation

All mouse procedures were performed in accordance with the ethical guidelines of the institute under the protocols approved by the Institutional Animal Care and Use Committee of RIKEN or the Animal Experimentation Committee of the Institute for Frontier Life and Medical Sciences, Kyoto University. For the CE2-nutrient group, 6-week-old C57BL/6J male mice were purchased from CLEA Japan and maintained in the RIKEN facility (temperature: 23 °C ± 2 °C, humidity: ≲70%, and light: 24-hour light/dark cycle (lights on at 7:00 and off at 19:00)) for 3 days by being fed a CE-2 diet (CLEA Japan) in the same cage before sampling. For the VA group (Fig. 3a), 8-week-old C57BL/6J male mice were purchased from Japan SLC, Inc. and maintained under specific pathogen-free (SPF) conditions (temperature: 24 °C ± 2 °C, humidity: 50% ± 10%, and light: 24-h light/dark cycle (lights on at 8:00 and off at 20:00)) at the Experimental Research Center for Infectious Diseases in the Institute for Frontier Life and Medical Sciences, Kyoto University. In total, 12 mice were housed in groups of six per cage by being fed a sterile nutrition-balanced diet (VA-sufficient diet; designed based on AIN-93G Diet; A18041301, Research Diets, Inc.) (Supplementary Table 2) for 2 weeks; in the middle of the 2 weeks, three randomly selected mice in each cage exchanged cages. For subsequent vitamin A-dependent experiments, all 12 mice were randomly allocated into groups of three per cage. Two cages were continuously fed the VA-sufficient diet for 3 weeks, while the other two cages were fed a sterile VA-deficient diet (A21022401, Research Diets, Inc.; only vitamin A was not included compared to the VA-sufficient diet) (Supplementary Table 2) for 3 weeks. Two of the three mice in each cage were randomly selected for both BarBIQ and conventional method measurements.

### Mock community preparation

A mock community that consisted of ten reported human gut bacterial strains (ATCC29098, ATCC700926, DSM14469, JCM1297, JCM5824, JCM5827, JCM9498, JCM10188, JCM14656, and JCM17463) was prepared^40^ (details in Supplementary Table 1). Briefly, cultured bacteria of each strain were stored in the culture medium of the strain with 10% glycerol or in PBS (phosphate-buffered saline, Thermo Fisher Scientific or FUJIFILM Wako) at −80 °C until the experiments (Supplementary Table 1). Before the stocks were made, JCM14656 and DSM14469 cells were washed once with PBS using centrifugation after culturing. JCM10188 was cultured on GAM agar (Nissui); thereafter, the bacteria were collected from colonies and suspended in PBS by vortexing at 3200 rpm for 1 min (Vortex Genie 2, Scientific Industries).

The ten strains were diluted using PBS and then mixed according to the designed concentrations in a class II biosafety cabinet (Supplementary Table 1). Each step of dilution or mixing was followed by vortexing at 3200 rpm for 1 min. We called this the mixed 10-strain mock community. The mock community was stored at −80 °C until the experiments.

### Microscopy measurements of bacterial concentration

The concentration of each strain was determined by fluorescence microscopy imaging. Bacteria that were fluorescently stained using propidium iodide (Thermo Fisher Scientific) and heating at 70 °C for 5 min were counted in a volume that was measured using polystyrene microspheres (Bacteria Counting Kit from Thermo Fisher Scientific). The volume was calculated based on the predetermined concentration of the microspheres, which had been measured using a Bacteria Counting Chamber (Sunlead Glass). For each strain, five independent measurements were performed. Details are provided in Supplementary Note 4.

### Sanger sequencing of 16S rRNA genes

For each of the ten strains, the 16S rRNA genes were amplified from bulk bacteria using 2 × KAPA HiFi Hot start ready mix (Roche) and primers F1-full-Fw/F3-full-Rv (Supplementary Table 3) and were purified using a Zymo DNA concentration & clean kit (Zymo Research). For the cecal cell-sample Dd^prox^, the 16S rRNA gene(s) of each bacterial cell was amplified separately in droplets (the ratio between bacteria and droplets was ~4%) using the Bio-Rad Droplet Digital^TM^ PCR (ddPCR) system with primers (final concentrations of 400 nM F1-full-Fw and 400 nM F3-full-Rv), 1× ddPCR^TM^ Supermix for Probes (No dUTP) (Bio-Rad), a final concentration of 0.13 unit/µl of Platinum Taq (Invitrogen), and a final concentration of 100 nM dNTPs (New England Biolabs). The amplified 16S rRNA genes in droplets were recovered using chloroform (Sigma) and further purified using AMPure XP magnetic beads (Beckman Coulter) and gel purification (2% E-Gel^TM^ EX Agarose Gels, Thermo Fisher Scientific). For each sample (a strain or Dd^prox^), the amplified 16S rRNA genes were subsequently cloned and amplified in *E. coli* using the Zero Blunt TOPO PCR Cloning Kit (Thermo Fisher Scientific). Then, the 16S rRNA genes were amplified individually from randomly selected single colonies of *E. coli* using the primers T7-Promoter and SP6-Promoter (Supplementary Table 3) and 2× KAPA HiFi Hot start ready mix. Finally, the V3–V4 region (see the main text) of the amplified 16S rRNA gene from each colony was sequenced using the F1-Fw and F2-Rv primers (Supplementary Table 3) by Sanger sequencing (FASMAC); one strand was sequenced for the ten strains, and both strands were sequenced for the cell-sample Dd^prox^.

### Murine cecal content collection

The murine ceca were exteriorized by surgery within 10 min after cervical dislocation under sevoflurane (FUJIFILM Wako) or isoflurane (FUJIFILM Wako) anesthesia. The cecal contents at two locations (Fig. 1b) were sampled by slicing using sterile scissors. The sampling process was performed in a class II biosafety cabinet within 10 min after the surgery. Samples of each location in each mouse (see main text) were collected in a DNA LoBind tube (Eppendorf). For controls, two empty tubes were subjected to the whole process of cecal content sampling. The sample weight was measured immediately after collection into the DNA LoBind tubes and ranged from 8.57 to 19.82 mg for the samples in the CE2-nutrient group and from 1.7 to 5.6 mg for the samples in the VA group. Each sample of CE2-nutrient group was dispersed in PBS (50 μl per 1 mg) and mixed by vortexing at 3200 rpm for 1 min. The suspended samples were stored at 4 °C until further experiments (in 1 day). The samples of VA group were immediately frozen in liquid N_2_ and stored at −80 °C. For further experiments, the samples were thaw, dispersed in PBS (50 μl per 1 mg), and mixed by vortexing at 3200 rpm for 1 min.

### Separation of extracellular DNA

The murine cecal samples were diluted accordingly using PBS (1 ml PBS per 1 mg cecal content) followed by vortexing at 3200 rpm for 1 min. For the empty tubes (i.e., control), the same volume of PBS matching the lightest sample was added. Then, 400 µl of the diluted sample was filtered using a 0.22-µm pore size Ultrafree^®^-MC Centrifugal Filter (Merck) by centrifugation (10,000 × *g*, 10 mins, 4 °C). The sample remaining above the filter membrane (cell-sample) was suspended in 400 µl of PBS by pipetting, and the suspended cell-sample was subsequently transferred into a new DNA LoBind tube and was vortexed at 3200 rpm for 1 min. The suspended cell-sample and the flow-through containing extracellular DNA (ecDNA-sample) were stored at 4 °C until further measurements (see Supplementary Note 5).

### Conventional 16S rRNA sequencing method

The detailed protocol is described elsewhere^61^. Briefly, bacteria of the mock community or each cecal cell-sample in the VA group were suspended in PBS and sequentially subjected to lysozyme, achromopeptidase, and proteinase K for cell lysis. Then, DNA was recovered by phenol-chloroform extraction. The V3–V4 region of the 16S rRNA genes was amplified using region-specific primers containing Illumina adapter overhang nucleotide sequences (CONV-341F and CONV-805R in Supplementary Table 3). The amplicons were purified using AMPure XP magnetic beads and indexed using the Nextera XT Index Kit v2 (Illumina). Following purification using AMPure XP, the pooled libraries were qualified and quantified by a TapeStation (Agilent) and the KAPA Library Quantification Kit for Illumina platforms (Kapa Biosystems). Denatured libraries were spiked with 20% PhiX control v3 (Illumina) and sequenced on a MiSeq platform (2 × 300 bp paired-end reads, Illumina). For the OTU-based analysis, sequence data were checked for their quality and trimmed using Trimmomatic (version 0.38)^62^. OTUs were clustered at a 97% identity threshold using Mothur (version 1.35.1) by following its instructions^41^. The most abundant sequence in each OTU was selected as its representative sequence. For the ASV-based analysis, sequence data were analyzed using DADA2 (version 1.20.0)^19^ by following its instructions; Read 1 and 2 were truncated to 280 and 200 bp, respectively.

### BarBIQ procedures

#### Design of cellular barcodes

Four types of cellular barcode templates, each containing twenty-four random bases and six fixed bases (Barcode-1 to Barcode-4; Supplementary Table 3), were designed according to our previous publication^29^. We confirmed from the sequencing results that the number of random bases in the barcodes was sufficient for distinguishing individual cells in a single MiSeq sequencing run (Supplementary Note 6).

#### Total abundance measurement

The total abundances per unit weight or volume of cells or ecDNAs were measured by Bio-Rad ddPCR using primers F1-Fw and F1-Rv (Supplementary Note 1 and Supplementary Table 3). The concentrations of four equimolar-mixed cellular barcodes were also measured by ddPCR using primers NoBiotin-Link-barcode-F and P5-index-R1P-barcode-R (Supplementary Table 3). ddPCR was performed according to the user’s manual for QX200^TM^ ddPCR^TM^ EvaGreen^®^ Supermix (Bio-Rad).

#### Single-step in-droplet amplification

To generate a sequencing library, ~240,000 cells for each cell-sample, ~20,000 copies of ecDNAs for each ecDNA-sample in CE2-nutrient group, or ~240,000 copies of ecDNAs for each ecDNA-samples in VA group were mixed with ~160,000 copies of the equimolar-mixed cellular barcodes, primers (final concentrations of 400 nM P7-R2P-341F, 400 nM P5-index-R1P-barcode-R, 10 nM Biotin-Link-805R, and 10 nM Biotin-Link-barcode-F) (Supplementary Table 3), 1× ddPCR^TM^ Supermix for Probes (No dUTP), 128 units of Platinum Taq, and a final concentration of 100 nM dNTPs in 960 μl of solution. After vortexing at 3200 rpm for 1 min, the mixed solution was encapsulated into droplets by a Bio-Rad droplet generator: 30 μl of mixed solution and 80 μl of Droplet Generation Oil for Probe (Bio-Rad) were loaded for each channel on a DG8^TM^ Cartridge (32 channels were used for each sample). For mock community measurements, ~600,000 cells were mixed with ~600,000 copies of the cellular barcodes, 320 units of Platinum Taq, primers (final concentrations of 400 nM P7-R2P-341F, 400 nM P5-index-R1P-barcode-R, 10 nM Biotin-Link-805R, and 10 nM Biotin-Link-barcode-F), 1 × ddPCR^TM^ Supermix for Probes (No dUTP), and a final concentration of 100 nM dNTPs in 2400 μl of solution. Then, after vortexing, the mixed solution was encapsulated into droplets using 80 channels per sample. The library for MiSeq sequencing was generated by single-step PCR in droplets (5 min at 95 °C; 6 cycles of 45 s at 94 °C and 150 s at 60 °C; 49 cycles of 25 s at 94 °C and 80 s of 60 °C; 10 min at 98 °C).

#### Library recovery and purification

The library generated by the in-droplet amplification was recovered using chloroform; 80 μl of TE buffer (Invitrogen) and 280 μl of chloroform were mixed with droplets collected from eight wells of the DG8^TM^ Cartridge, which was followed by pipetting 10 times and vortexing until the water and organic phases separated; after centrifugation (21,900 × *g*, 10 min, room temperature), the water phase solution containing the library was extracted. Then, nontarget DNAs such as unlinked barcode amplicons, remaining primers, and byproducts in the recovered solution were removed by bead purification (AMPure XP magnetic beads) and gel purification (2% E-Gel^TM^ EX Agarose Gels). Subsequently, the biotinylated unlinked 16S rRNA amplicons were removed by streptavidin magnetic beads (New England Biolabs) (Supplementary Fig. 4)^36^. The purification steps using AMPure XP beads, gels, and streptavidin beads were performed twice. Finally, the purified libraries were concentrated by a Zymo DNA Clean and Concentrator Kit. The quality of each library was checked by an Agilent 2100 Bioanalyzer, and the concentration was determined by qPCR (KAPA SYBR Fast qPCR kit, Kapa Biosystems) using primers P1_qPCR_Fw and P2_qPCR_Rv (Supplementary Table 3). The protocols of the purification steps using AMPure XP beads, gels, and streptavidin beads basically followed the instructions from the suppliers.

#### MiSeq sequencing

The libraries of samples were paired-end sequenced on a MiSeq platform (MiSeq Reagent Kit v3, 600 cycles, Illumina) by allocating 30 cycles for Read 1, 295 cycles for Index 1, 8 cycles for Index 2, and 295 cycles for Read 2 (Supplementary Fig. 4). The Illumina Index 1 sequencing primer was replaced by a custom primer named I1_primer (Supplementary Table 3) to read the 16S rRNA sequences instead of the indexes. To maintain the heterogeneity of sequences for sequencing, spike-in controls generated in our laboratory were co-sequenced with the samples (details in Supplementary Note 1). We confirmed that the sequencing depths in all sequencing runs were sufficient for accurate counting (Supplementary Note 7).

#### Pipeline for data processing

We developed a pipeline for processing data obtained by sequencing to identify Bar sequences and cOTUs and to quantify each cOTU. The main strategies for the pipeline are shown in Supplementary Fig. 5, and the details of each step are described in Supplementary Note 2. In principle, reads from MiSeq were first clustered using cellular barcodes (read R1)^29^. Then, 16S rRNA sequences (reads I1 and R2) linked to the same cellular barcode were further clustered based on their sequence identities. A representative sequence (RepSeq) for each clustered 16S rRNA sequence group was generated based on both the number of reads for each sequence type and their sequencing qualities. After removal of possible errors basically depending on the number of reads, the remaining RepSeq sequence types were named BarBIQ-identified sequences (Bar sequences). The Bar sequences were then grouped into cOTUs based on their codetection frequency in the same droplets. If two or more Bar sequences were frequently detected in the same droplets, we considered these multiple 16S rRNA genes to be detected from the same bacterium in a droplet. Next, the number of cells for each cOTU was determined by the number of unique cellular barcodes (i.e., the barcode clusters) that were linked to the cOTU. The absolute cell abundance per unit weight or volume of each cOTU was determined by normalizing the sequencing-determined count of cells using the total cell abundance of the sample measured by ddPCR. In addition, contaminated cOTUs during the sampling and/or measurement were identified by a control.

Most parts of the pipeline were written in Perl (Version 5.22.1), and others were implemented by R (Version 3.5.1 or 4.1.1), nucleotide-sequence-clusterizer (Version 0.0.7)^29^, and bwa (Version 0.7.15)^63^ software. The modules in Perl and packages in R used in this pipeline are listed in Supplementary Table 4.

### Comparison with 16S rRNA gene databases

The identity shared between an identified Bar sequence and its closest (i.e., highest identity) 16S rRNA gene sequence in three public databases, GreenGenes (Release 13_5)^46^, Ribosomal Database Project (Release 11.5)^47^, and Silva (Release 138; SSU Ref NR 99)^48^, was calculated using NCBI BLAST (Version 2.7.1)^64^; when the coverages of all hits obtained by BLAST were less than 100%, the identity was calculated using the Perl module Text::Levenshtein::XS (Nick Logan).

### Taxonomy prediction by the RDP classifier

Taxa from the phylum to the genus level of identified cOTUs were predicted based on their Bar sequence(s) by the RDP classifier using a bootstrap cutoff of 50%^65^. The RDP classifier was trained by 16S rRNA training set 16 (available from the RDP at https://rdp.cme.msu.edu/)^47^. For a cOTU containing multiple Bar sequences, the predicted taxon showing the highest score was selected.

### Bray–Curtis dissimilarity

Bray–Curtis dissimilarity between each pair of samples based on the relative abundances or the absolute abundances of the detected cOTUs was calculated using the function vegdist in the R package vegan. The relative abundances were normalized among samples using the median of ratios method in DESeq2 (Version 1.32.0)^49^. Analyses, Bray-Curtis dissimilarity calculations, estimation of technical noise, and differential abundance analyses were carried out using R (version 3.5.1 or 4.1.1) by JupyterLab (Version 0.34.9).

### Estimation of technical noise

We confirmed that noise in cOTU concentration measurements among the technical replicates of the sample CEa^dist^ measured by BarBIQ mainly resulted from sampling by comparing the measured technical noise to simulated noise assuming a Poisson distribution in the sampling process. To exclude bias from different numbers of totally detected cells in the technical replicates, we normalized the cell count of each replicate by sub-sampling using the function rarefy in the R package vegan. The normalized total counts of all replicates were the same as their smallest unnormalized total count. The noise of a cOTU was quantified by CV2: here, CV represents the coefficient of the variation, which was calculated based on the normalized cell counts of the cOTU in three technical replicates^66,67^. The simulated noise for each cOTU was calculated using three numbers (to mimic three technical replicates) randomly generated from a Poisson distribution with a mean determined by the averaged cell count of the given cOTU in the sample. We then calculated the theoretical mean-corrected residual (*R*_mc_) of CV^*2*^ for each cOTU as follows:

$${R}_{{{{{{{\mathrm{mc}}}}}}}}={{\log }}_{10}({{{{{{\mathrm{CV}}}}}}}^2)-{{\log }}_{10}({{{{{{{{\mathrm{CV}}}}}}}}_{{{{{{{\mathrm{Poisson}}}}}}}}}^{2})$$

where CV_Poisson_ is the theoretical CV for the given cOTU assuming a Poisson distribution. The distribution of all *R*_mc_ values of the sample CEa^dist^ was consistent with those of the simulations, which suggested that the technical noise in BarBIQ was mainly from sampling (Supplementary Fig. 12c–e).

### Differential abundance analysis

All differential abundance analyses were performed using DESeq2 (Version 1.32.0)^49,53^ with the parameters *betaPrior* = *TRUE* and *minreplicatesforreplace* = *Inf*. For the differential abundance analyses based on relative abundances, the cOTUs, ASVs, or OTUs with raw counts ≥ 5 in at least three samples in each pair of groups were used, and subsequently, the sequencing-determined raw counts of the cOTUs, ASVs, or OTUs were normalized using the median of ratios method in DESeq2. The FDR (padj in DESeq2), log-2-fold change, and the standard error of the log-2-fold change for each cOTU, ASV, or OTU were calculated using DESeq2.

### Reporting summary

Further information on research design is available in the Nature Research Reporting Summary linked to this article.

## Supplementary information

Supplementary Information file

Description of Additional Supplementary Files

Supplementary Data 1

Supplementary Data 2

Supplementary Data 3

Supplementary Data 4

Supplementary Data 5

Supplementary Data 6

Supplementary Data 7

Supplementary Data 8

Reporting Summary

## Data Availability

The sequencing data are available from the NCBI Sequence Read Archive under accessions PRJNA639639, PRJNA639647, PRJNA780331, and PRJNA780361. The databases, GreenGenes (Release 13_5) [https://greengenes.secondgenome.com/], Ribosomal Database Project (Release 11.5) [https://rdp.cme.msu.edu], Silva (Release 123.1 and 138; SSU Ref NR 99) [https://www.arb-silva.de], and rrnDB (version 5.7) [https://rrndb.umms.med.umich.edu] are available online. Source data are provided with this paper.

## Source data

Source Data

## Code availability

The BarBIQ pipeline is available at github (https://github.com/Shiroguchi-Lab/BarBIQ).
