# Supplementary material for: High-throughput identification and quantification of single bacterial cells in the microbiota
Source: Nat Commun. 2022 Feb 22;13:863. doi: 10.1038/s41467-022-28426-1 (PMC8863893; doi:10.1038/s41467-022-28426-1)
Supplement: Supplementary file 1 — Supplementary Information file [file 41467_2022_28426_MOESM1_ESM.pdf]

Supplementary Information
for
High-throughput identification and quantification of single bacterial cells in the microbiota

Jianshi Jin, Reiko Yamamoto, Tadashi Takeuchi, Guangwei Cui, Eiji Miyauchi, Nozomi Hojo, Koichi Ikuta, Hiroshi Ohno, Katsuyuki Shiroguchi*

*Correspondence should be addressed to: Katsuyuki Shiroguchi (katsuyuki.shiroguchi@riken.jp)

Table of Contents

Supplementary Notes 4

(1) BarBIQ measurement in detail 4
Total abundance measurements by two sets of primers 4
Bacterial concentration adjustment for droplet generation 5
Control for BarBIQ measurement of the mock community 6
Spike-in control for BarBIQ sequencing 6

(2) BarBIQ data processing in detail (steps 1-17, Supplementary Fig. 5) 6
Step 1: Clustering based on cellular barcodes 6
Step 2: Trimming the low-quality 3' end and the primer part of reads I1 and R2 7
Step 3: Clustering by 16S rRNA sequences (I1 and R2) 7
Step 4: Generating a representative sequence (RepSeq) for each SCluster 8
① Correcting possible errors 1 (step 5) 8
Step 5: Correcting shifted RepSeqs 8
Step 6: Linking I1 and R2 RepSeqs 9
② Correcting or removing possible errors 2 (steps 7–9) 10
Step 7: Correcting one insertion and deletion (1-Indel) RepSeqs 10
Step 8: Removing chimeras 11
Step 9: Correcting other Indel-related and substitution errored RepSeqs 11
Step 10: Counting BClusters for each RepSeq in each index 12
③ Removing possible errors 3 (steps 11–12) 12
Step 11: Removing low-count RepSeq types 12
Step 12: Removing RepSeq types with single substitution errors 12
Step 13: Naming RepSeq types as Bar sequences 13
Step 14: Retrieving false-negative RepSeq types 13
Step 15: Identifying multiple Bar sequences from the same bacterium 13
Step 16: Counting the number of cells for each cOTU 16
Step 17: Removing contaminated cOTUs 16

(3) Breaking bacterial clumps by vortexing 17

(4) Microscopy measurements of bacterial abundance in detail 18

(5) Evaluation of separation of extracellular DNA (ecDNA) 19

(6) Barcode length 20

(7) Sequencing depth 20

Supplementary Figures 21

Supplementary Figure 1: Breaking bacterial clumps 21

Supplementary Figure 2: Counting bacteria in droplets by microscopy imaging. 23

Supplementary Figure 3: Barcode encapsulation in droplets and heating time for cell lysis. .. 25

Supplementary Figure 4: Schematic with sequence information for library generation, purification, and sequencing in BarBIQ. 26

Supplementary Figure 5: Schematic of BarBIQ data processing. 27

Supplementary Figure 6: Unique San sequences, ASVs, and OTU-RepSeqs. 29

Supplementary Figure 7: Bacterial counting by microscopy imaging. 31

Supplementary Figure 8: Comparison between the proportional abundance of ASV and strains measured by microscopic imaging. 33

Supplementary Figure 9: Quality controls for the separation of ecDNA and cells. 34

Supplementary Figure 10: Difference from San sequence to its closest Bar sequence, ASV, or OTU-RepSeq, and difference among 16S rRNA sequences in the database 36

Supplementary Figure 11: Total cell abundance per unit weight of each cell-sample. 38
Supplementary Figure 12: Sampling noise for cOTU abundance in the BarBIQ measurement. 39
Supplementary Figure 13: Comparison of the quantification between different methods. 41
Supplementary Figure 14: Difference between cell abundance and 16S rRNA gene abundance for cecal cell-samples in the VA group. 42
Supplementary Figure 15: Bray-Curtis dissimilarity. 44
Supplementary Figure 16: Comparison of location-dependent cell abundance in each mouse. 45
Supplementary Figure 17: Differential cell abundance of cOTUs between locations. 47
Supplementary Figure 18: Dietary vitamin A deficiency-based differential abundance analyses using cOTUs, ASVs, or OTUs 48
Supplementary Figure 19: Quantification of ecDNA in murine ceca. 50
Supplementary Figure 20: Comparison between ddPCR measurements using primer sets F1-Fw/F1-Rv and 341F/805R for the same sample. 51
Supplementary Figure 21: Four types of DNA as the spike-in control. 53
Supplementary Figure 22: Logic diagram of step 3.2 54
Supplementary Figure 23: Logic diagram of step 5. 55
Supplementary Figure 24: Distribution of the lengths of 16S rRNA genes at the V3–V4 region. 56
Supplementary Figure 25: Logic diagram of step 7. 57
Supplementary Figure 26: Logic diagram of step 8. 58
Supplementary Figure 27: Logic diagram of step 9. 59
Supplementary Figure 28: Distribution of average count ratios between pairs of RepSeq types that had one substitution 60
Supplementary Figure 29: Identifying multiple Bar sequences from the same bacterium. 61
Supplementary Figure 30: Comparison of average cOTU counts between the mock community and the control M0. 63
Supplementary Figure 31: Dependence of the number of clusters (unique barcodes) on the number of random bases designed in the barcodes. 64
Supplementary Figure 32: Dependence of the counts of cOTUs on the average number of reads per unique barcode. 65
Supplementary Tables 66
Supplementary Table 1: Information for the ten cultured strains 66
Supplementary Table 2: Diet formulation for vitamin A-sufficient and vitamin A-deficient diets. 67
Supplementary Table 3: Sequences of primers and barcodes used in all experiments. 68
Supplementary Table 4: Perl modules or R packages used in the data analysis. 69
Supplementary Table 5: Information on the presented sequencing runs. 70
Supplementary References 73

Supplementary Notes

(1) BarBIQ measurement in detail

Total abundance measurements by two sets of primers

The total abundances of bacterial cells, extracellular DNA (ecDNA), and cellular barcodes per unit volume or weight were measured by Droplet Digital^{TM} PCR (ddPCR) according to the instructions supplied with the QX200^{TM} ddPCR^{TM} EvaGreen$^{®}$ Supermix (Bio-Rad). For cell- and ecDNA-samples, the primers F1-Fw and F1-Rv, targeting the V1–V2 region of the 16S rRNA gene, or 341F and 805R, targeting the V3–V4 region of the 16S rRNA gene, were used (Supplementary Table 3). For cellular barcodes, the primers NoBiotin-Link-barcode-F and P5-index-R1P-barcode-R (with index GTACTGAC) were used (Supplementary Table 3). The QX200^{TM} ddPCR^{TM} EvaGreen$^{®}$ Supermix, final concentrations of 1 μM each primer and 1 μM dNTPs (New England Biolabs), and sample (multiple dilutions, with vortexing at 3,200 rpm for 1 min for each dilution, were performed) were mixed in a volume of 30 μl and pipetted for mixing. Then, the mixed solution was encapsulated into droplets using Droplet Generation Oil for EvaGreen (Bio-Rad), a DG8^{TM} Cartridge (Bio-Rad), and a droplet generator (Bio-Rad). Droplet PCR was performed using the following steps: 5 min at 95 °C for initial denaturation; 6 cycles of 45 sec at 95 °C for denaturation and 150 sec at 60 °C for annealing and extension; 39 cycles of 25 sec at 95 °C (for F1-Fw and F1-Rv) or 34 cycles of 25 sec at 95 °C (for primers 341F and 805R) for denaturation, and 80 sec at 60 °C for annealing and extension; and 5 min of 4 °C and 5 min of 90 °C for signal stabilization. Subsequently, the fluorescence intensities of the droplets were measured by a QX200 Droplet Reader (Bio-Rad), and the numbers of positive and negative droplets were determined based on a threshold that was the valley of the bimodal distribution of the intensities by QuantaSoft software (Bio-Rad) (Supplementary Fig. 20a). Finally, the abundances per unit volume or weight of the samples were calculated based on the ratios of positive to negative droplets and the dilution of samples.

We measured the total abundances of both cells and ecDNA per unit weight for the same sample (a cecal sample obtained from a C57BL6/J male mouse that was not shown in the main text) using both primer-sets, F1-Fw/F1-Rv and 341F/805R, and confirmed that the measured abundances of the two were consistent. For this reason, we used the primer pair F1-Fw/F1-Rv for the total abundance measurements of the bacterial samples in BarBIQ.

For this comparison, we determined the proportions of positive and negative droplets by Gaussian fitting due to unclear separation between distributions of positive and negative droplets in the cases using 341F/805R (Supplementary Fig. 20b). We fitted the peaks of the

intensity distributions using four Gaussian distributions and the function normalmixEM in the R package mixtool (Supplementary Fig. 20c). Fitting with two Gaussian distributions may be sufficient: one for the positive droplets and the other for the negative droplets. However, the data showed that there were apparently more than two Gaussian distributions. Therefore, we fitted the intensity distribution with different numbers of Gaussian distributions. We found that the proportion of positive droplets was stable when we used four or more Gaussian distributions (≤ 6 were tested) (Supplementary Fig. 20d), which suggested that four Gaussian distributions were sufficient to explain the intensity distribution. To calculate the proportion of positive droplets, we assumed a fitted Gaussian distribution for positive droplets if the mean of this Gaussian distribution was larger than an apparent valley of the intensity bimodal distribution, and vice versa for the negative droplets. Finally, we compared the proportion of positive droplets between the results using the two primer sets and found that they were not different for both the bacterial cell- and ecDNA-samples (Supplementary Fig. 20e). Because the separation between positive and negative droplets using primers F1-Fw/F1-Rv was much clearer than that using 341F/805R (Supplementary Fig. 20a,b), we chose primers F1-Fw/F1-Rv for BarBIQ.

Bacterial concentration adjustment for droplet generation

To barcode each bacterium uniquely for accurate cell-based quantification and identification, we used 250 cells/μl bacteria for droplet generation (see Methods). This concentration resulted in a ratio between the number of cells and the number of droplets of 1:5 since the volume of the droplet was approximately 0.8 nl, which was calculated from the diameter of the droplet. Assuming a Poisson distribution, 82% of the droplets did not contain a bacterial cell, 16% contained one cell, 2% contained two cells, and < 0.01% contained more than two cells. Therefore, if all bacterial cells were the same type (multiple cells in the same droplet that cannot be distinguished), 100 barcoded bacteria, as an example, were counted as 90 ($\approx 100 \times 5 \times (0.16 + 0.02)$) bacteria by BarBIQ, meaning that the determined count would be approximately 90% of the cell number of the barcoded bacteria. Since in our measurements, bacterial cell types were diverse, the maximum fraction of cell numbers for cell types was < 0.4 for the mock community and < 0.21 for the cecal samples. Therefore, the determined count in our measurement was > 96% ($\approx 100 \times 5 \times (0.074 + 0.003)/(100 \times 0.4)$) of the cell number of the barcoded bacteria for the mock community and > 98% ($\approx 100 \times 5 \times (0.0403 + 0.0009)/(100 \times 0.21)$) for the cecal samples. Conversely, the high co-occurrence of different

bacteria in the same droplet may affect the identification of different 16S rRNA sequences and cOTUs (Supplementary Note 2, step 15). All things considered, the selected bacterial concentration of 250 cells/µl was appropriate for both bacterial quantification and identification in BarBIQ.

Control for BarBIQ measurement of the mock community

An additional cecal sample (M0) acquired from another C57BL6/J male mouse without filtration was measured by BarBIQ and was only used as a control for the measurement of the mock community. Three independent measurements were performed for the control. For each measurement, 240,000 cells were mixed with 240,000 copies of the cellular barcodes, 128 units of Platinum Taq (Invitrogen), primers (final concentrations of 400 nM P7-R2P-341F, 400 nM P5-index-R1P-barcode-R, 10 nM Biotin-Link-805R, and 10 nM Biotin-Link-barcode-F), 1 × ddPCR™ Supermix for Probes (No dUTP) (Bio-Rad), and a final concentration of 100 nM dNTPs in 960 µl of solution. After vortexing, the mixed solution was encapsulated into droplets using DG8™ Cartridges (32 channels per measurement). The following steps were the same as those for the mock community measurements.

Spike-in control for BarBIQ sequencing

Four types of spike-in controls were mixed with libraries and co-sequenced to avoid unbalanced base types in sequencing, as is often performed using PhiX in amplicon sequencing[1] (Supplementary Fig. 21).

(2) BarBIQ data processing in detail (steps 1-17, Supplementary Fig. 5)

In our sequencing, R1 (30 bases) was a cellular barcode, I1 (295 bases) and R2 (295 bases) were 16S rRNA sequences from both ends, and I2 (8 bases) was an index; the indexes uniquely labelled samples, technical replicates, controls for contamination, and spike-in controls, respectively, in each sequencing run (Supplementary Fig. 4). All five sequencing runs (1, 2, 3, 4, and 5) are summarized in Supplementary Table 5.

Step 1: Clustering based on cellular barcodes

Reads of cellular barcodes (R1) were clustered based on their sequences as carried out in our previous report[2] except for the initial deletion of low-quality reads. First, we deleted the low-quality R1 reads that contained at least one window of four continuous bases with an average

sequencing quality score (determined by MiSeq) less than 15, as widely found[3]. Fractions of reads in sequencing runs 1, 2, 3, 4, and 5 (0.23%, 0.05%, 0.06%, 0.08%, and 0.07%, respectively) were deleted by this process. Then, the R1 reads in which the last four fixed bases were identical to the designed bases were selected for the next step. All the selected R1 reads were clustered for each sequencing run using nucleotide-sequence-clusterizer[2] software with a distance of 2. When more than one index was present in the same cluster, the number of reads in the cluster for each index was checked, and low-abundance reads were deleted; if the numbers of reads were equal, all reads were deleted. The obtained clusters were named barcode clusters (BClusters). Then, I1 and R2 from the same DNA molecule were linked to R1 using the MiSeq sequence IDs. Finally, the clusters were grouped based on the indexes.

After step 1, 57%, 86%, 87%, 93%, and 93% of reads in sequencing runs 1, 2, 3, 4, and 5, respectively, were retained. The remaining fraction of reads in sequencing run 1 was low, possibly because relatively short nontargeted DNAs remained in the library, as observed using a Bioanalyzer.

Step 2: Trimming the low-quality 3' end and the primer part of reads I1 and R2

We trimmed the 3' ends of both I1 and R2 reads, which tend to contain errors due to their low sequencing qualities[4]. To determine the trimming length for each sequencing run, average quality scores for two consecutive bases were calculated for all positions and all reads. When we first found (from 5' to 3' end) that the average quality score was < 25, we trimmed the bases from the second position to the 3' end. By this process, we trimmed 64 bases (I1) and 101 bases (R2), 1 (I1) and 28 (R2), 24 (I1) and 58 (R2), 1 (I1) and 20 (R2), and 25 (I1) and 57 (R2) for sequencing runs 1, 2, 3, 4, and 5, respectively.

We also trimmed some bases from the 5' end that corresponded to the designed primers for 16S rRNA gene amplification: 21 bases for I1 and 17 for R2. We removed reads I1 and R2 containing an undetermined base shown as "N" in the Illumina platform, which represented approximately 0.4% of the reads.

Step 3: Clustering by 16S rRNA sequences (I1 and R2)

In step 3, the two clustering processes below were performed for the trimmed I1 and R2 reads (*i.e.*, 16S rRNA sequences) in each BCluster (see step 1) based on sequence identities.

<u>Step 3.1: Clustering based on substitution distance</u>

We generated a single sequence (referred to as I1-R2 read) by tandemly linking the trimmed I1 and R2 for each MiSeq ID (sequenced molecule). The generated I1-R2 reads in each BCluster were clustered into sub-clusters (SClusters) based on the substitution distance using nucleotide-sequence-clusterizer software with a distance parameter of 3.

Step 3.2: Clustering based on a single position of the reads

To separately identify very similar sequences that might be real 16S rRNA sequences rather than errors, we clustered all I1-R2 reads in each SCluster from step 3.1 based on base types at a particular position of the I1-R2 reads (Supplementary Fig. 22). The workflow was as follows. i) For all bases, a converted score was calculated from the sequencing quality score at a given base: when the sequencing quality score was < 15, the converted score was 0, and when the sequencing quality score was ≥ 15, the converted score was equal to the sequencing quality score divided by 41 (highest sequencing quality score). ii) In each SCluster, a value for each base type (A, T, C, or G) in each position was calculated as the sum of the converted scores from the same base in the same position (four values corresponding to four base types were calculated for each position). ii) For each position, a ratio of the second highest value to the highest value was calculated. iii) The position of the I1-R2 reads with the highest ratio among all positions was selected. iv) If the ratio of the selected position was ≥ 0.75, the I1-R2 reads that contained the base type corresponding to the second highest value were separated from the original SCluster and made a separate BCluster. v) The same process was carried out for the original and separated SClusters until the ratio became lower than 0.75. vi) SClusters containing only one read were deleted.

We note that step 15 includes the same process as step 3.2 with a different threshold for a different purpose.

Step 4: Generating a representative sequence (RepSeq) for each SCluster

For each SCluster from step 3.2, representative sequences (RepSeqs) for I1 and R2 reads were generated. For each position of the reads, a value for each base type (A, T, C, or G) was calculated in an SCluster using the same strategy as in step 3.2. The base type with the highest value was used as the base type in the RepSeq at the given position (Supplementary Fig. 5).

① Correcting possible errors 1 (step 5)

Step 5: Correcting shifted RepSeqs

We corrected shifted RepSeqs generated by insertion or deletion errors at amplification primer sites (5' end of the I1 and R2 reads). We named the shifted RepSeq Shift and the correct RepSeq (an original of a Shift) Mother (Supplementary Fig. 23). We performed the following steps for I1 and R2 RepSeqs independently in each index (Supplementary Fig. 23): i) All possible pairs of Mother and Shift for RepSeqs in each BCluster that had < 8 shifted bases were found. ii) For each found pair (RepSeq-A and RepSeq-B), the number of BClusters that satisfied the following conditions was counted: BClusters containing RepSeq-A (No[A]), RepSeq-B (No[B]), both RepSeq-A and RepSeq-B where the number of reads for generating RepSeq-A was greater than that of RepSeq-B (No[A>B]), and both RepSeq-A and RepSeq-B where the number of reads for generating RepSeq-B was greater than that for RepSeq-A (No[A<B]). iii) If No[A] > No[B] and No[A>B] > No[A<B], RepSeq-A was considered Mother and RepSeq-B Shift; if No[A] < No[B] and No[A>B] < No[A<B], RepSeq-B was considered Mother and RepSeq-A Shift; if neither of the above cases occurred, the pair was not considered Mother or Shift because error sequences are generally rarer than correct sequences. iv) If RepSeqs in any BCluster were the same as one of the determined Shifts, the RepSeqs were corrected; in case 1, if a Shift had only one Mother, then the Shift was corrected to its Mother, while in case 2, if a Shift had multiple Mothers, then the Shift was corrected to a Mother whose number of BClusters was the highest among the Mothers.

Step 6: Linking I1 and R2 RepSeqs

I1 and R2 RepSeqs were linked by their overlapping 3' end sequences. Because the length of 16S rRNA genes at the V3–V4 region, which are defined by the two primers 341F and 805R (Supplementary Table 3), were almost 400 to 500 bp depending on the Silva database (v123.1)[5] (Supplementary Fig. 24), sequencing of 295 bases for both I1 and R2 reads may basically result in more than 90 (295×2–500) overlapping bases between the I1 and R2 reads. However, due to the low sequencing qualities at the 3' end, the number of bases used for data processing was limited (see step 2), resulting in 0, 61, 8, 69, and 8 overlapping sequences for sequencing runs 1, 2, 3, 4, and 5, respectively, assuming that the length of the V3–V4 region was 500 bases. Therefore, the I1 and R2 RepSeqs in sequencing run 1 (including the mock community and its control) were not linked, while those in sequencing runs 2, 3, 4, and 5 (murine cecal samples and their controls) were linked.

We linked I1 and R2 when the number of overlapped bases was more than five: the possibility of accidental overlapping is $(1/4)^b$, where b is the number of overlapped bases, and

the possibility in the case of 5 bases is $(¼)^5 \approx 0.00098$. Thus, no overlap was found in sequencing run 1, while all reads were linked in sequencing runs 2, 3, 4, and 5.

To remove substitution errors in the overlap, we performed the following steps: i) unlinked RepSeqs were compared to linked RepSeqs within the same BCluster (the I1 and R2 RepSeqs were compared, respectively). ii) If there was a linked RepSeq containing one base difference in the overlap from the unlinked RepSeq, the unlinked RepSeq was corrected to be the same as the linked RepSeq.

② Correcting or removing possible errors 2 (steps 7–9)

Step 7: Correcting one insertion and deletion (1-Indel) RepSeqs

We corrected RepSeqs with possible errors generated by one insertion or deletion (1-Indel) in the linked RepSeqs from step 6. Reads with errors containing indels were separated in different SClusters, which made individual RepSeqs, because the clustering in step 3 was based on the substitutions. In this step, we corrected the 1-Indels, and in step 9, two-base indels, a 1-Indel with one substitution, and a 1-Indel with two substitutions.

To correct the 1-Indels, we performed the following steps for RepSeqs in each index (Supplementary Fig. 25). i) All possible pairs of 1-Indel and Mother (an origin of the 1-Indel) for RepSeqs in each BCluster were found. ii) For each found pair (RepSeq-A and RepSeq-B), the number of BClusters that satisfied the following conditions were counted: BClusters containing RepSeq-A (No[A]), RepSeq-B (No[B]), both RepSeq-A and RepSeq-B where the number of reads for generating RepSeq-A was greater than that of RepSeq-B (No[A>B]), both RepSeq-A and RepSeq-B where the number of reads for generating RepSeq-B was greater than that for RepSeq-A (No[A<B]), BClusters containing RepSeq-A but not RepSeq-B (No[A-only]), and BClusters containing RepSeq-B but not RepSeq-A (No[B-only]). iii) If No[A] > No[B], No[A>B] > No[A<B], and No[B-only] < 3, RepSeq-A was considered Mother and RepSeq-B a 1-Indel; if No[A] < No[B], No[A>B] < No[A<B], and No[A-only] < 3, RepSeq-B was considered Mother and RepSeq-A a 1-Indel; if neither of the above cases occurred, then the pair was not considered a 1-Indel or Mother because errored sequences are generally rarer than correct sequences. iv) If RepSeqs in any BCluster were the same as one of the determined 1-Indel sequences, then the RepSeqs were corrected. In case 1, if a 1-Indel sequence had only one Mother, then the 1-Indel was corrected to its Mother sequence, while in case 2, if a 1-Indel sequence had multiple Mothers, then the 1-Indel was corrected to the sequence of a Mother with the highest number of BClusters among the Mothers.

Step 8: Removing chimeras

We removed chimeras, which were mainly generated during the amplification. Chimeras often occur during PCR amplification and are a common problem in 16S rRNA amplicon measurements[6].

We performed the following steps for RepSeqs in each index (Supplementary Fig. 26). i) All possible triples of a chimera and its parents (the origins of the chimera) for RepSeqs in each BCluster were found; if the 5' end of RepSeq (RepSeq-A) was the same as the 5' end of another RepSeq (RepSeq-B), the part of RepSeq-A that differed from RepSeq-B at the 3' end was the same as the 3' end of a third RepSeq (RepSeq-C), and RepSeq-A did not have the highest number of reads among the three RepSeqs, then we considered RepSeq-A a possible chimera and RepSeq-B and RepSeq-C the possible parents. ii) For each identified triple of possible chimera and parents, the number of BClusters satisfying the following conditions was counted: BClusters containing the chimera (Total_No[Chimera]) and BClusters containing the chimera but not its parents (No[Chimera-only]). iii) If No[Chimera-only]/Total_No[Chimera] ≤ 0.1 or No[Chimera-only] = 1, then the possible chimera was considered a real chimera. iv) If the RepSeqs in any BCluster were the same as the real chimeras, then the RepSeqs were deleted.

BarBIQ had only 1–5% chimeras, which is much lower than the number (up to 70%) obtained by conventional methods[6]. A possible reason for the small number of chimeras generated in BarBIQ is that 16S rRNA amplicons from different bacteria did not mix in the amplification step because single-step amplification was performed basically in separate spaces (droplets) for each bacterium. This approach has not been performed, even in recent studies on high-throughput 16S rRNA gene sequencing using droplets and barcodes[7,8].

Step 9: Correcting other Indel-related and substitution errored RepSeqs

As mentioned in step 7, in this step, we first corrected three types of RepSeqs with indel-related errors: type 1, which has one indel with one substitution; type 2, which has one indel with two substitutions; and type 3, which has two indels. Generally, complicated errors occurred less frequently than simple errors, and such complicated errors were deleted in step 12.

We performed the following steps for RepSeqs in each index (Supplementary Fig. 27): i) all possible pairs of RepSeqs in each BCluster that had one of the above differences (types 1, 2, and 3) were found. ii) For each found RepSeq pair, if the ratio of the number of reads between

the RepSeqs (small one/large one) was lower than 0.2, then we considered the small one as a RepSeq with an error, assuming that the large one was its original RepSeq of the error, and corrected the RepSeq with an error to the original one.

We also corrected, using the same procedure as above, another type of RepSeqs with substitution (< 5) errors that were generated by the shifted error correction in step 5.

Step 10: Counting BClusters for each RepSeq in each index

For each unique RepSeq (RepSeq type), we counted the number of BClusters containing the given RepSeq type in each index.

③ Removing possible errors 3 (steps 11–12)

Step 11: Removing low-count RepSeq types

We removed low-count RepSeq types because unexpected errors might occur. For the mock community sample, we removed RepSeq types when their average counts from three sampling replicates were < 2. To calculate the average counts, the counts of RepSeq types for each replicate were normalized by the total count of all RepSeq types, and the total count after normalization was the same as the highest unnormalized total count among three replicates. For the cecal samples in each index, we removed the RepSeq types with counts < 6 to avoid false positives since we performed only one sampling replicate.

In addition, in the mock community, one short RepSeq type (260 bases without primer sites) that was matched to the middle of the San sequences JCM5824-A and JCM5824-B (419 bases between the primer sites; Supplementary Data 2) was found. We interpreted that this short RepSeq type might have been generated by nontargeted priming to the 16S rRNA genes in strain JCM5824 based on three observations. i) Six bases in the middle of JCM5824-A/B matched the 3' end of the forward primer 341F (Supplementary Table 3), which was used for amplification. ii) This short sequence was always co-detected with JCM5824-A and/or JCM5824-B in the same droplet. iii) The numbers of BClusters containing this short RepSeq type were 2, 4, and 1 in the three sampling replicates Mock-a, Mock-b, and Mock-c. We did not find such short RepSeq types in the cecal samples; all RepSeq types identified were > 400 bases long without primer sites (Supplementary Data 1).

Step 12: Removing RepSeq types with single substitution errors

We removed RepSeq types with single substitution errors that might be generated by PCR amplification. We considered only one substitution error since PCR errors are generally rare,

and in this study, we did not find other error types of RepSeq types in the mock community sample.

For the mock community sample, we removed a RepSeq type if it had one substitution from another RepSeq type and if the ratio of the average count (see step 11) of the former RepSeq type to that of the latter RepSeq type was < 0.0025 (Supplementary Fig. 28). All the removed RepSeq types showed low average counts (≤ 8). We note that BarBIQ is able to detect two different bacteria containing 16S rRNA sequences that differ in a single base if the abundance of the less-abundant bacterium is > 0.0025 the abundance of the more-abundant bacterium in a sample.

For the cecal samples, we removed a RepSeq type if it had one substitution from another RepSeq type in the same index and the ratio of the count of the former RepSeq type to that of the latter RepSeq type was < 0.01, since we had only one sampling replicate.

We note that steps 11 and 12 were independent and that the processing order of these two steps did not affect the results.

Step 13: Naming RepSeq types as Bar sequences

We named the resulting unique RepSeq types from step 12 BarBIQ-identified sequences (Bar sequences), and each was labelled by an ID number.

Step 14: Retrieving false-negative RepSeq types

The removed RepSeq types in steps 11 and 12 that were processed in each index were retrieved if those RepSeq types were the same as one of the Bar sequences in other indexes for mice (without M0), expecting to reduce the number of false negatives generated in steps 11 and 12.

Step 15: Identifying multiple Bar sequences from the same bacterium

We identified multiple Bar sequences (*i.e.*, 16S rRNA sequences) for the same bacterium using the cellular barcodes by distinguishing a natural co-occurrence of Bar sequences of the same bacterium from an accidental co-occurrence of Bar sequences from different bacteria that existed in the same droplet. Ideally, multiple Bar sequences from the same bacterium always coexist in a droplet. Conversely, the frequency of accidental co-occurrence depends on the concentration of the given bacterium. Therefore, we compared the experimental co-occurrence with the theoretical random co-occurrence assuming a Poisson distribution; these may be significantly different under our experimental conditions with low concentrations of bacteria.

In addition to the simple thought process here, in real experiments, some Bar sequences might not be detected because the number of amplicons of multiple Bar sequences in a droplet may not always be similar. Therefore, we carefully determined a threshold for the discrimination of natural and random co-occurrences based on equations and statistics, including simulations, as stated below.

In each index, we considered all possible Bar-sequence pairs from step 14. For each Bar-sequence pair (Bar sequence A and Bar sequence B), the number of droplets that contained both Bar sequence A and Bar sequence B (*Experimental_Overlap*), the number of droplets that contained only Bar sequence A (named *A*), and the number of droplets that contained only Bar sequence B (named *B*) were counted. For this counting, we performed the same process as in step 3.2 with a threshold of 0.1 followed by steps 4 and 5 using the processed data from step 3.1 to achieve higher detection efficiency for multiple Bar sequences in the same droplet instead of using the threshold 0.75 for Bar sequence identification from step 3.2. Then, the number of droplets in which a pair of Bar sequences from different bacteria was present was calculated based on the Poisson distribution:

$$Poisson\_Overlap = \frac{A \times B \times \mu}{Droplets}$$

where *Droplets* is the total number of droplets containing cellular barcodes and μ is the detection efficiency for droplets containing different bacteria. We then converted the equation using $\log_{10}$ transformation:

$$log_{10}(Poisson\_Overlap) = log_{10}(A \times B) + log_{10}\left(\frac{\mu}{Droplets}\right)$$

We assumed μ was constant for different Bar-sequence pairs in each measurement, resulting in the term $log_{10}\left(\frac{\mu}{Droplets}\right)$ being constant for all Bar-sequence pairs; we named this term the operational droplet (*OD*).

Next, we estimated *OD* by fitting running medians of $log_{10}(Poisson\_Overlap)$ against $log_{10}(A \times B)$ using a model $y=x+OD$. In our measurements, *Experimental_Overlaps* were similar to *Poisson_Overlaps* because most pairs of Bar sequences were from different bacteria. Therefore, we used the running medians of $log_{10}(Experimental\_Overlap)$ instead of the unmeasurable $log_{10}(Poisson\_Overlap)$: the running medians may remove the noise caused by the pairs of Bar sequences that were from the same bacterium. The running medians of $log_{10}(Experimental\_Overlap)$ were obtained against $log_{10}(A \times B)$ with a 0.4 window and 0.2 overlap. The medians that were more than zero were used for fitting (red circles in Supplementary Fig. 29a).

After OD was obtained by fitting, we re-plotted $log_{10}(Experimental\_Overlap)$ against $log_{10}(A \times B) + OD$ (Supplementary Fig. 29b), which actually indicates the relationship between $log_{10}(Experimental\_Overlap)$ and $log_{10}(Poisson\_Overlap)$. Therefore, the data from different bacteria pairs should be on the line of $y=x$, though noise was seen, especially for low-abundance bacteria.

For the mock community sample, we found that the $log_{10}(Experimental\_Overlap)$ values of Bar-sequence pairs from the same bacterium were larger than those from different bacteria when their $log_{10}(A \times B) + OD$ values were similar. To statistically distinguish these two types of Bar-sequence pairs, we estimated the confidence intervals of the $log_{10}(Poisson\_Overlap)$ by simulation. First, we confirmed that for different values of A, B and OD, if the value of $log_{10}(A \times B) + OD$ is constant, $A=B$ showed the widest distribution. Therefore, we obtained the distribution of $log_{10}(Poisson\_Overlap)$ with a parameter A ($=B$), which was changed from 1 to 1,500 by 500,000 calculations for every integer using a fixed OD. Then, the one-sided confidence intervals for the distribution were calculated. When A was not equal to B, $A \times B$ was replaced by C^2 for the one-sided confidence interval, and C was the closest higher integer of $\sqrt{A \times B}$. We found that the upper 99.9% one-sided confidence intervals (UP999) distinguished the Bar-sequence pairs from the same bacterium and from different bacteria (Supplementary Fig. 29b). We note that the consistency between the experimentally measured co-occurrence of different strains in the same droplet (i.e., linked to the same barcode) and the theoretically calculated random co-occurrence assuming a Poisson distribution in three independent measurements (Supplementary Fig. 29b) suggested that the distribution of the bacteria in mock community followed a Poisson distribution.

However, when we applied this process to the cecal samples, false positives might occur because this process relied on statistics, and > 20,000 Bar-sequence pairs were analyzed in each sample. Indeed, in cecal samples, we found that the $log_{10}(Experimental\_Overlap)$ of some Bar-sequence pairs with Bar sequences mapped to different bacterial names in the public database Silva (v138)[5] was larger than UP999 (grey circles in Supplementary Fig. 29c).

To avoid such false positives, we utilized all the cecal cell-samples, including technical replicates to determine whether a pair of Bar sequences was from the same bacterium because the same bacteria in different samples have essentially the same 16S rRNA sequences. We chose the Bar-sequence pairs which satisfied the following conditions for further analyses: in at least two samples, the two Bar sequences in the pair were detected and the pair's *Experimental_Overlap* was not one. Then for each pair, we used the samples where the two

Bar sequences were detected and the *Experimental_Overlap* was not one, and calculated the ratio of the number of samples that exhibited a Bar-sequence pair log_{10}(*Experimental_Overlap*) larger than UP999 to the total number of samples (Ratio_Positive). All the Bar-sequence pairs from different bacteria based on the database Silva showed that Ratio_Positive was ≤ 0.5 (Supplementary Fig. 29d). Therefore, we used a threshold of Ratio_Positive > 0.5 to identify Bar-sequence pairs from the same bacterium. The accuracy of this analysis will increase when more datasets are obtained because the same bacteria in different samples have essentially the same 16S rRNA sequences.

Finally, if we found common Bar sequences in different same-bacterial Bar sequences, we concluded that all these Bar sequences were in one bacterium. We named each of unique same-bacterial Bar sequence(s) a cell-based Operational Taxonomic Unit (cOTU).

Step 16: Counting the number of cells for each cOTU

The number of cells for each cOTU was determined by the number of cellular barcodes (number of BClusters) linked to each cOTU in each index. The different RepSeqs detected in the same BCluster were considered to be from the same cell if they were identified as the same cOTU by step 15. We note that the identification of bacteria depends on the target sequences used (V3–V4 region in this study); it does not work for bacteria that have identical target sequences. However, one may easily use different target sequences in the same protocol here.

Step 17: Removing contaminated cOTUs

We removed contaminated bacteria using a control, M0, for the mock community, or empty tube controls for the cecal samples in the CE2-nutrient and VA groups.

For each replicate of the mock community, the final cell count of the detected cOTUs was obtained by subtracting the average count of the cOTUs in the control; the average count in the control was calculated from three replicates of the control with normalization by the total number of droplets. For subtraction, two different methods were applied for two different conditions (Supplementary Fig. 30). (i) If the average count of a cOTU from three replicates of the mock community minus the standard error of the average count was lower than the average count of the cOTU plus the standard error in the control, then the final count of the cOTU in the mock community became zero (*i.e.*, the cOTU was removed). (ii) If the condition in (i) was not satisfied for a cOTU, then the final count of the cOTU in the mock community was obtained by subtracting the average count of this cOTU in the control from the original

count of the cOTU in the mock community. In case (ii), this process changed fewer than 1% of the counts for these cOTUs.

For the cecal samples in the CE2-nutrient and VA groups, only one sampling replicate was measured. Therefore, to statistically compare the counts in samples and controls, we estimated the errors for counts in the samples based on the Poisson sampling noise (square root of the count of each cOTU). For the controls for the cecal samples, *i.e.*, two or three empty tubes, we calculated the mean and standard deviation (SD) for the count of each cOTU (n = 2 or 3). We used a 99.9% confidence interval (3.27 × SD) as the error, and when n = 2, we set the minimum error as 10% of the mean. We removed contaminants from these samples using essentially the same methods as for the mock community: (i) if the count of a cOTU minus the calculated error in the sample was lower than the average count of the cOTU plus the calculated error in the control, then the cOTU in the sample was removed, and (ii) if the condition in (i) was not satisfied for a cOTU, then the final count of this cOTU in the sample was obtained by subtracting the average count of the cOTU in the control from the original count of the sample. We note that we did not perform normalization for comparison between the cOTU counts in the cecal samples and empty tube controls because the number of droplets used was almost the same (see Methods).

By these processes, approximately 0.5%, 4% and 3% of cells were considered to be contaminants in the mock community, the cecal samples in the CE2-nutrient group, and the cecal samples in the VA group, respectively. The relatively large fraction of the contaminants in the cecal samples was mainly due to one cOTU (different one in CE2-nutrient and VA groups) that showed similar detected cell numbers in all samples and controls.

(3) Breaking bacterial clumps by vortexing

We broke bacterial clumps (an example in Supplementary Fig. 1a) using vortexing (3,200 rpm, 1 min) in the BarBIQ measurement. We confirmed by fluorescence microscopy imaging (see Methods and Supplementary Note 4) that the bacterial clumps were broken after vortexing. We observed the shapes of fluorescence-illuminated spots for each of the ten strains after vortexing and found that most spots contained one dot, but some contained multiple dots (examples of both cases are shown in Supplementary Fig. 1b). We then analyzed the distribution of the number of dots per spot and showed the average number of dots per spot for each strain (Supplementary Fig. 1c,d). The dot-per-spot distribution of the nine strains ranged from 1.0 to 1.3, while that of JCM10188 was 2.0, which might be due to the different culture

condition compared with the others (see Methods)[9]. Multiple dots in a spot might indicate a dividing cell or an incomplete cell fission, which is often seen in bacteria[10]. We comprehensively concluded that we successfully broke clumps of bacteria by vortexing.

Next, we applied this vortexing method to a cecal sample and observed the sample by fluorescence microscopy. The dot-per-spot distribution of the cecal sample was similar to those of the nine strains above other than JCM10188, and its average dot-per-spot was 1.1 (Supplementary Fig. 1c,d). In fact, we observed 208 spots for the cecal sample and found that 22 spots had multiple dots (Supplementary Fig. 1e). These multiple-dot spots apparently contained similarly shaped dots in each spot; only one spot (the yellow arrow in Supplementary Fig. 1e) contained two kinds of shaped dots. These results suggested that bacterial clumps also did not exist in the cecal sample after vortexing.

(4) Microscopy measurements of bacterial abundance in detail

We measured the cell abundances per unit volume of the ten bacterial strains individually by fluorescence microscopy imaging (ex: 532 nm, em: 572 nm). Before imaging, bacteria were stained with 0.1 mg/ml propidium iodide (PI, Thermo Fisher Scientific) with heating at 70 °C for 5 min after vortexing (3,200 rpm, 1 min). We confirmed that the cell abundance of *E. coli* (DH5α) determined by fluorescence imaging with PI was consistent with that determined by phase contrast illumination (Supplementary Fig. 7a). Then, we chose fluorescence illumination because it was not always easy to distinguish dust from bacteria, especially small ones, by phase contrast illumination.

To decrease the thermal motion of bacteria in a solution, which may affect their counting, a chamber made of two coverslips (24×50 mm; Matsunami Glass) without any spacer was used for observation (Supplementary Fig. 7b). PI-stained bacteria and polystyrene microspheres (Bacteria Counting Kit from Thermo Fisher Scientific) were observed together by fluorescence and/or phase contrast using an Olympus IX81 microscope with a 20× objective. For each chamber, seven randomly selected fields were illuminated for counting. The fluorescence signal was sufficiently intense to be separated from the background so that a threshold to remove the background was determined by eye (an example with strain ATCC700926 is shown in Supplementary Fig. 7c–e). We counted the multiple-dot spots as single cells by assuming that the multiple-dot spot was a single bacterium that was dividing or showed incomplete fission (see Supplementary Note 3).

(5) Evaluation of separation of extracellular DNA (ecDNA)

We performed multiple experiments to confirm that the separation of ecDNA and cells by filtration using a 0.22-μm pore size Ultrafree®-MC Centrifugal Filter (Merck) was reliable. We first compared Ultrafree®-MC Centrifugal Filters with pore sizes of 0.1, 0.22, and 0.45 μm. The amount of ecDNA in the flow-through using the 0.22-μm filter was similar to those using the 0.1-μm and 0.45-μm filters, which suggested that the separation of the ecDNA was not size dependent in the range from 0.1 μm to 0.45 μm (Supplementary Fig. 9a). The abundances of cells (*i.e.*, filter-residue) recovered from the filter membrane by suspension using PBS (Thermo Fisher Scientific) (see Methods) were comparable in the case of 0.1-μm and 0.22-μm filters but lower when a 0.45-μm filter was used. This phenomenon might be due to the lower recovery efficiency of cells from the 0.45-μm filter membrane than from the other two.

We then observed both cells in the flow-through and the recovered filter residue from the 0.22-μm filter membrane by fluorescence imaging with PI (see Methods and Supplementary Note 4). The number of bright spots that were supposed to be cells in the flow-through was almost zero, suggesting that almost all the cells remained in the filter-residue (Supplementary Fig. 9b). Furthermore, the number of bright spots observed in the filter-residue was consistent with the abundance measured by ddPCR (Methods, Supplementary Fig. 9b).

Finally, we compared two methods for the separation of ecDNA and cells, filtration and centrifugation (21,900 × *g*, 5 min, 4 °C). We found that the abundances of both the ecDNA and cells separated by filtration were consistent with those obtained by centrifugation (Supplementary Fig. 9c).

The results of these control experiments suggested that the separation of ecDNA and cells in the cecal samples by filtration using the 0.22-μm filter was reliable. We note that cecal samples used for these control experiments are supplemental to those shown in the main text; these were acquired from C57BL/6J male mice and have been stocked for more than one year at –80 °C. The abundance of ecDNA indicates the number of fragmented DNA molecules containing detectable 16S rRNA genes because vortexing was applied for the ecDNA samples. Extending the duration of vortexing did not change the measured abundance of the ecDNA, suggesting that the vortexing fragmented DNA molecules but did not break the ecDNA into shorter fragments than 16S rRNA genes (Supplementary Fig. 9d).

We measured the total abundances per unit weight of the separated ecDNA, separated cells, and unfiltered-samples of the cecal contents obtained from CEa, CEb, CEc, and CEd mice. The sums of the total abundances per unit weight of separated cells and ecDNAs was comparable

to the total abundance per unit weight of their unfiltered-sample (Supplementary Fig. 9e), suggesting that the recovery efficiency of both cells and ecDNAs was high after the filtration step.

Next, we used sequencing to measure the absolute abundances per unit weight of cOTUs in the cell-samples, or cOTUs and uniquely detected Bar sequences in the ecDNA-samples and unfiltered-samples from CEa mouse. The sums of absolute abundances of detected cOTUs (or unique Bar sequences) in the separated cell- and ecDNA-samples were highly correlated (Pearson's $r \geq 0.95$) to the absolute abundances of the detected cOTUs (or unique Bar sequences) in the unfiltered-sample for the samples from CEa mouse (Supplementary Fig. 9f, g), which suggested that the filtration process did not have significant cOTU-specific bias.

(6) Barcode length

We confirmed that the number of random bases in our designed barcode was sufficient for digital measurements, as performed in our previous publication[2]. We trimmed random bases in the sequenced barcodes from the 3' end and counted the number of clusters as a function of the number of remaining random bases (Supplementary Fig. 31). The results showed that having more than 16 random bases did not further increase the number of clusters, indicating that having 24 random bases was sufficient for measuring approximately 10^5 bacteria in a single MiSeq sequencing run.

(7) Sequencing depth

We confirmed that the sequencing depths of sequencing runs 1, 2, and 3 were sufficient for digital measurements[2]. To investigate how many reads per unique barcode was sufficient for digital measurement by BarBIQ, we randomly sampled a fraction (1%, 2.5%, 5%, 10%, 25%, 50%, and 100%) of the reads in the results of Mock-b in sequencing run 1 (Supplementary Fig. 32a), $CEa^{dist}1$ and $CEa^{dist}3$ in sequencing run 2, and CEb^{dist} and CEc^{dist} (Supplementary Fig. 32b) in sequencing run 3 using the software Fastq-tools-0.8 (version 0.8--1), and counted the number of cells for each cOTU. The results showed that the cell number of each cOTU was saturated when the average number of reads per unique barcode was more than 70. In our measurement, the average number of reads per unique barcode in each index of sequencing runs 1, 2, 3, 4, and 5 was > 70.

Supplementary Figures

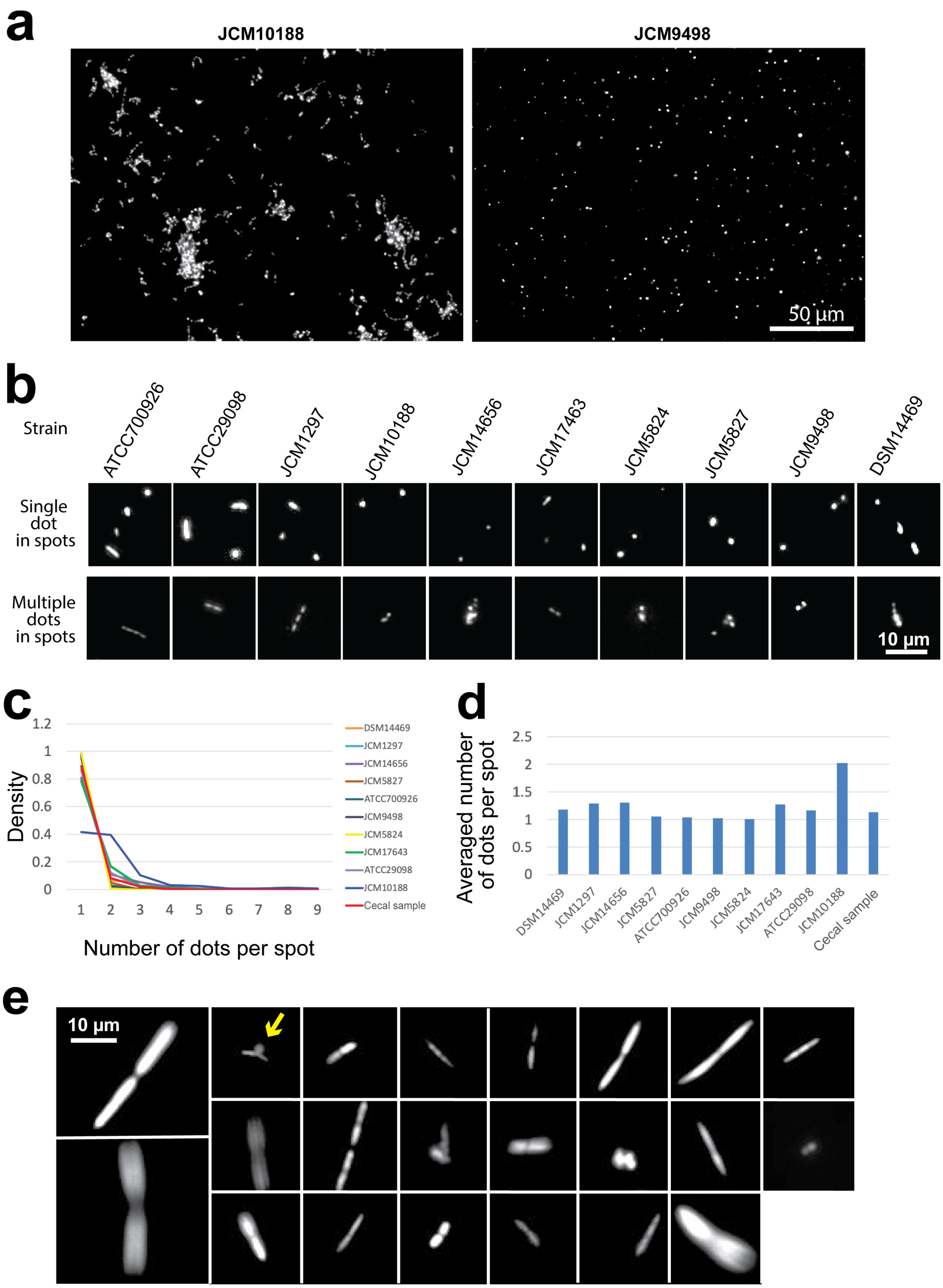

Supplementary Figure 1: Breaking bacterial clumps.

a, Bacterial clumps were present in strain JCM10188 but not in the other nine strains (JCM9498 is shown as an example) before vortexing. Similar results were found in five independent experiments. **b,** Examples of spots containing one dot or multiple dots after vortexing. Similar results were found in five independent experiments. **c,** Distribution of the number of dots per spot for each strain and a cecal sample after vortexing. **d,** Average number of dots per spot for each strain and the cecal sample after vortexing. **e,** All 22 spots containing multiple dots from the 208 analyzed spots in five independent experiments of the cecal sample. Yellow arrow, the only case that had two differently shaped dots. The contrast of the images shown in (**a**), (**b**), and (**e**) changed linearly. Source data for (**c**) and (**d**) are provided as a Source Data file.

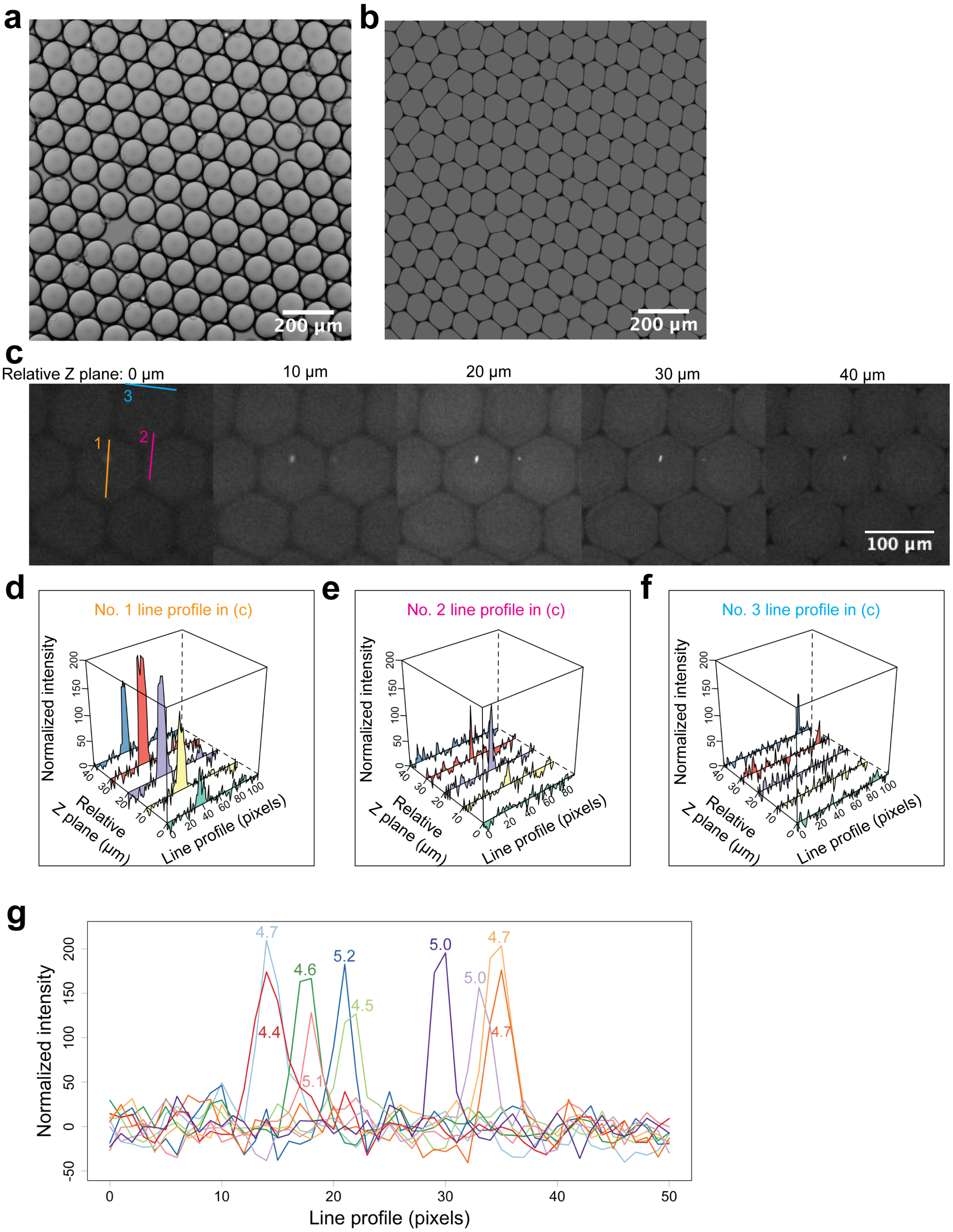

Supplementary Figure 2: Counting bacteria in droplets by microscopy imaging.

DNA in the bacteria of a cecal cell-sample was stained with 0.1 mg/ml PI with heating at 70 °C for 5 min after vortexing (3,200 rpm, 1 min) (see Supplementary Note 4) and was encapsulated into droplets by the Bio-Rad ddPCR system. Then, the droplets were loaded into a Countess^{TM} cell counting chamber slide (Invitrogen). The droplets were spread as a single

layer since the depth of the chamber was 100 μm. After 20 min, the droplets stopped moving in the chamber. Images were captured with both bright-field and fluorescent images of the droplets at multiple positions of the chamber by Z-scanning using a Nikon A1R confocal Ti2-E microscope system with NIS-Elements C. For both the bright-field and fluorescent images, LU-N4 Laser Unit 405/488/561/640 (wavelength: 561.1 nm; laser power: 40.0; gain: 40), A1-SHR-LFOV Scan Head (scan direction: one way; scanner zoom: 0.720; scan speed: 0.35; line average mode: average; line average/integrate count: 4; scanner selection: Galvano) with dichroic mirror (405/488/561/640 nm) and pinhole (size = 129.0 μm), and Plan Apo 10× objective (for (**a**) and (**b**)) or Plan Apo λ 20× Ph2 DM objective (for counting bacteria, see below) were used. For bright-field images, an A1-DUT Diascopic Detector Unit (HV:80; PMT offset:0) was used. For fluorescent images, an A1-DUVB-2 GaAsP Detector Unit with variable bandpass mode and emission wavelengths of 580.0~660.0 nm was used. Z-scanning with a step size of 10 μm and a total range of 150 μm that covered the whole droplets was performed using Ti2 ZDrive (Nikon). The number of bright spots (which were supposed to be bacteria) in fluorescent images in each droplet in which the boundary was determined by both bright-field and fluorescent images were quantitatively analyzed as below and were finally counted by eye. **a** and **b,** Examples of bright-field images of droplets after the droplets were loaded in a Countess^{TM} chamber for approximately 5 min (**a**) and 20 min (**b**). Similar results were found in three independent experiments. **c,** Examples of Z-scanned fluorescent images of bacteria in droplets. The colored lines were used for line profiles of intensity measurements by ImageJ[11]. Numbers, the plots in (**d**), (**e**), and (**f**). The contrast of images in (**a-c**) was changed linearly. **d, e,** and **f,** Intensities (gray value) measured along the lines in (**c**). Normalized intensity, the measured intensity was subtracted by the median of all measured intensities in each line profile. For all three bright spots, more than one Z-plane showed clear signals, suggesting that the Z-scanning step size of 10 μm was sufficient to detect all labeled bacteria in 3D of which the intensity was comparable to or higher than these three spots. **g,** Ten line profiles of the counted spots that apparently showed the lowest intensities in images (judged by eye). Normalized intensity, the same as (**d**), (**e**), and (**f**); numbers, signal/noise: maximum of normalized intensity/standard deviation of the normalized intensities of all pixels in the given profile. The cecal cell-sample here was prepared from the distal location of another mouse (eight-week-old C57BL/6N female) using the same protocol as the CE2-nutrient mice (Methods); the experiments above were performed immediately after the sampling. Source data for (**d-g**) are provided as a Source Data file.

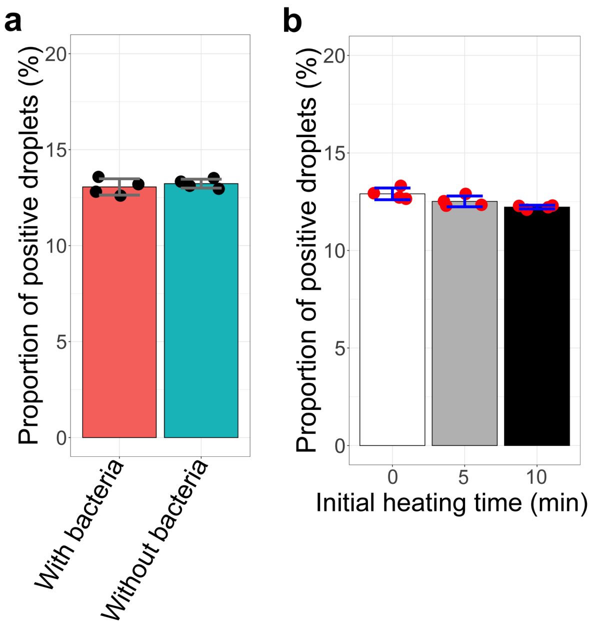

Supplementary Figure 3: Barcode encapsulation in droplets and heating time for cell lysis. **a,** The effect of bacteria on cell barcode encapsulation in droplets. The following conditions were compared: (i) a solution containing a certain concentration of barcodes and bacteria of a cecal cell-sample (concentrations were comparable to the BarBIQ measurement), primers for amplification of the barcode, and reagents for ddPCR (Supplementary Note 1); (ii) the same solution as (i) but without the bacteria. Two solutions were encapsulated into droplets using the Bio-Rad ddPCR system. After ddPCR, the proportion of droplets that had positive fluorescent signals was determined by the Bio-Rad QX200 Droplet Reader (Supplementary Note 1). The proportion of positive droplets was comparable between these two conditions, indicating that the bacteria did not affect cell barcode encapsulation. Data are presented as mean values +/- SD (n = 4). **b,** The proportion of positive droplets depending on the time of the initial heating step of ddPCR (Supplementary Note 1). 16S rRNA genes of a cecal cell-sample were amplified with different times of the initial heating step. The proportion of positive droplets did not depend on time, suggesting that the in-droplet amplification of the 16S rRNA genes from bacterial cells, including cell lysis, was robust. The cecal cell-sample used for both experiments here was prepared from the distal location of another six-week-old C57BL/6J male mouse (same as the CE2-nutrient mice); the sample preparation protocol was the same as the VA-group mice (Methods). Data are presented as mean values +/- SD (n = 4). Source data are provided as a Source Data file.

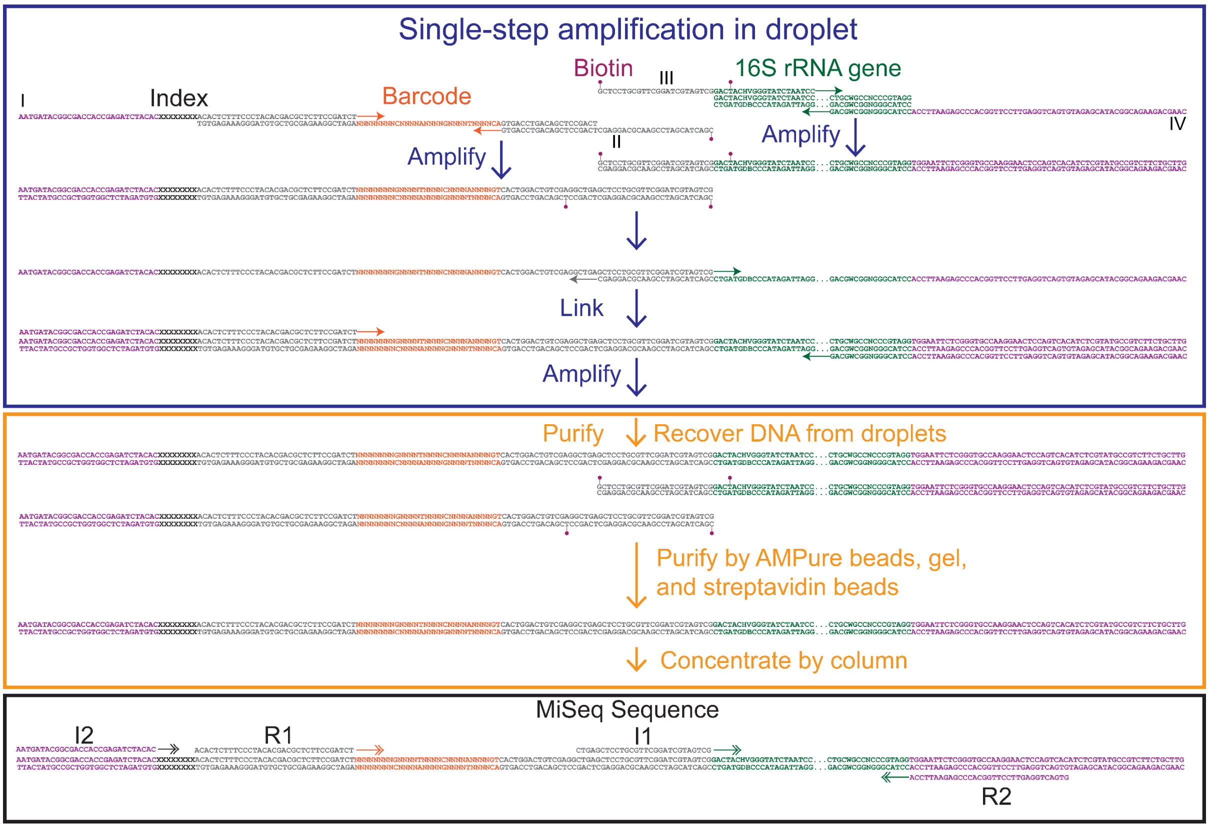

Supplementary Figure 4: Schematic with sequence information for library generation, purification, and sequencing in BarBIQ.

Details in Methods. I, II, III, and IV are the designed primers named P5-index-R1P-barcode-R, Biotin-Link-barcode-F, Biotin-Link-805R, and P7-R2P-341F, respectively, listed in Methods and Supplementary Notes; Index (XXXXXXXX), designed eight base sequence; Barcode, random and fixed bases (the other three designed barcodes are in Supplementary Table 3); Link, linking PCR; N in sequence, A, C, G, or T; I2, R1, and R2, Illumina sequencing primers for MiSeq; I1, customized sequencing primer.

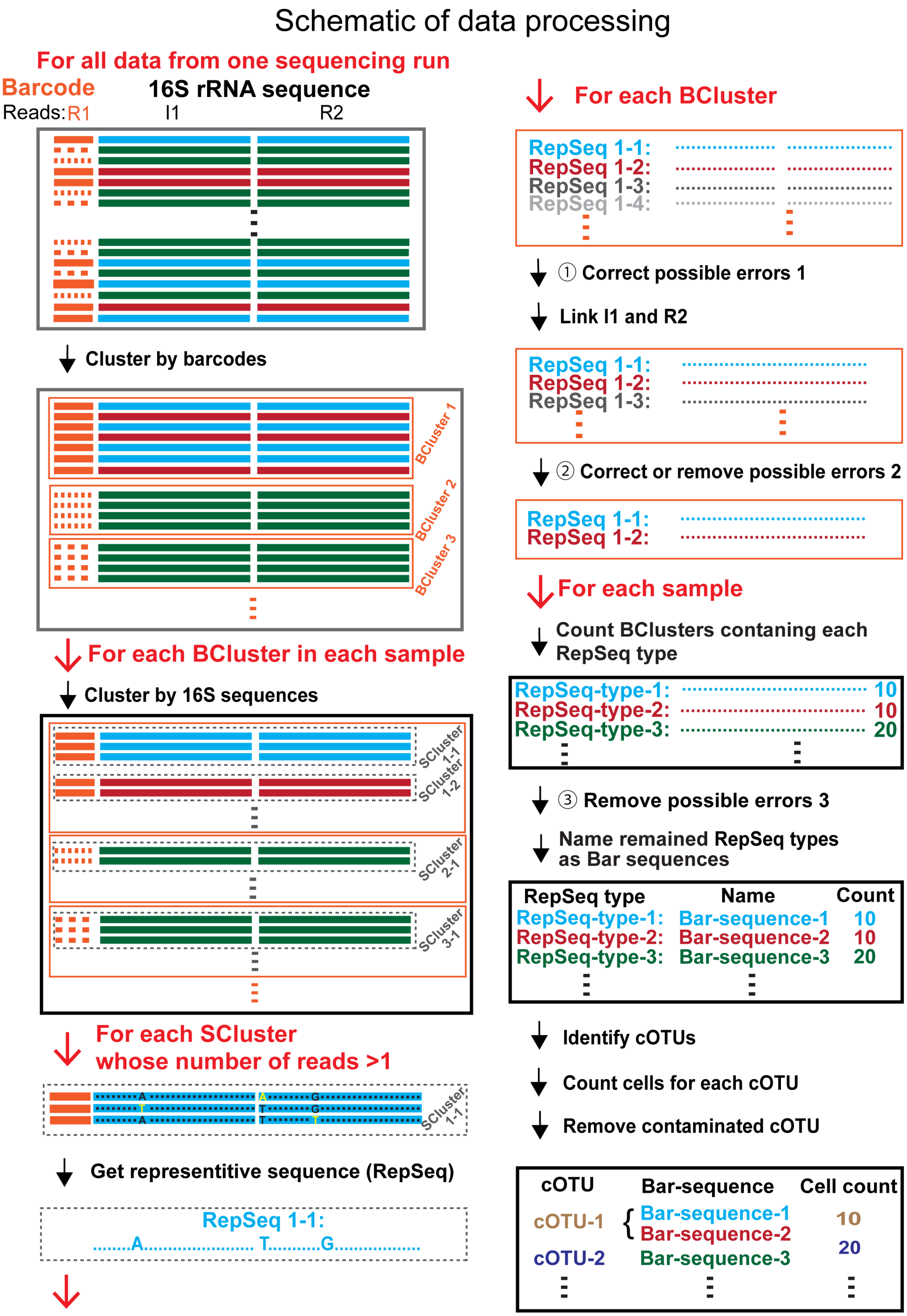

Supplementary Figure 5: Schematic of BarBIQ data processing.

Black arrows, processing steps, which are detailed in Methods and Supplementary Note 2; red arrow, the operand for the next steps; Barcode, cellular barcode; R1, R2 and I1, R1, R2 and I1 reads; BCluster, a cluster clustered by barcode; SCluster, a subcluster clustered by 16S rRNA sequences in each BCluster. ①, One step to correct the possible incorrect representative sequences (RepSeqs) for I1 and R2; Link I1 and R2, I1 and R2 RepSeqs linked using their overlapped sequences at the 3’ end of sequences. ②, Three steps to correct or remove multiple types of possible incorrect RepSeqs (linked), including substitutions, insertions, deletions, and chimeras, basically based on their low numbers of reads. ③, Two steps to remove possible incorrect unique RepSeqs (RepSeq-types) depending on the abundances of each RepSeq type. Bar sequences, BarBIQ-identified sequences; cOTUs, cell-based operational taxonomy units (see main text).

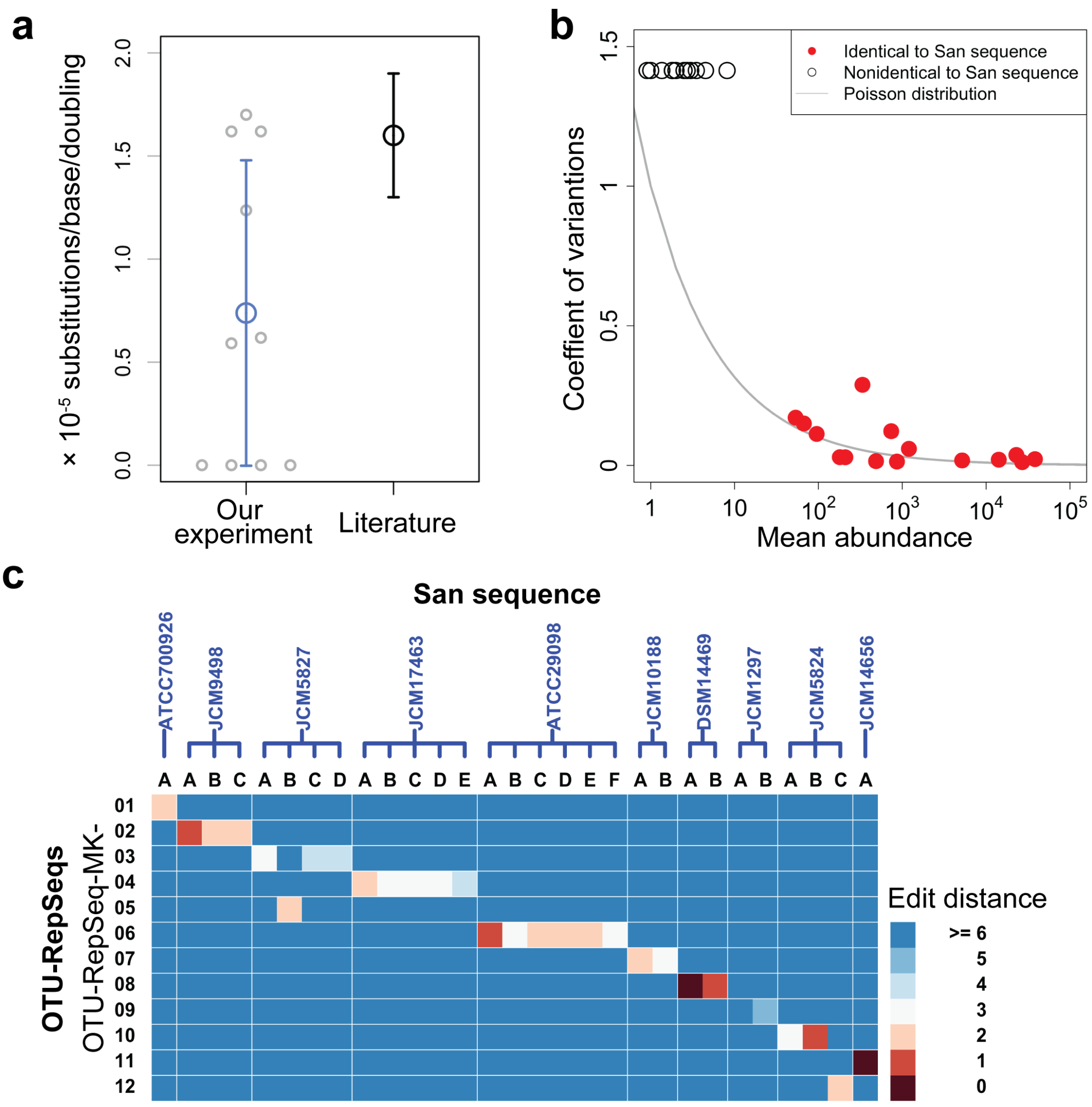

Supplementary Figure 6: Unique San sequences, ASVs, and OTU-RepSeqs.

a, Calculated substitution rate for each strain. For each strain, the substitution rate was calculated as the total number of substations between the San sequences of all colonies with its closest Bar sequence divided by the total length of the San sequences and the PCR cycles (38) we used in 16S rRNA gene amplification for a given strain. The calculated substitution rates were on average comparable to the amplification error rate measured in the literature[12], suggesting that San sequences that were not identical to any Bar sequence were possibly generated by amplification. Data are presented as mean values +/- SD (n = 10 in Our experiment). **b,** Coefficient of variance (mean divided by standard deviation) of the raw abundances of ASVs from two technical replicates as a function of the mean of the ASV abundances. Identical to San sequence, the ASV that was identical to one of the San sequences; Nonidentical to San sequence, the ASV that was not identical to any San sequence; Poisson

distribution, theoretical line based on Poisson distribution. **c.** Comparison between the 16S rRNA sequences identified by Sanger sequencing and by the conventional OTU-based analysis. OTU-RepSeq-MK-<number>, sequences representing OTUs; other labels, the same as in Fig. 2a. Source data are provided as a Source Data file.

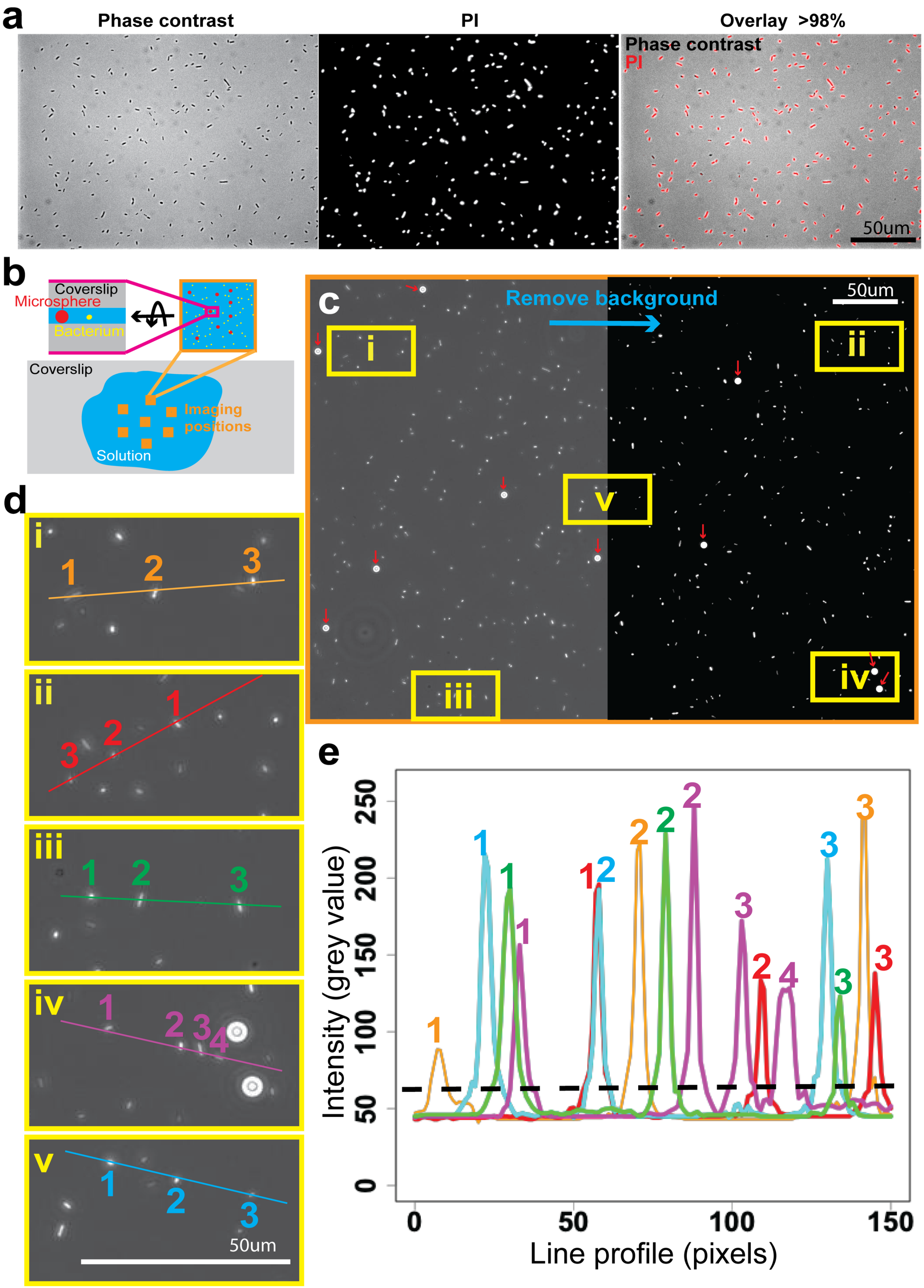

Supplementary Figure 7: Bacterial counting by microscopy imaging.

a, Comparison between phase contrast illumination and fluorescence microscopy (PI; see Supplementary Note 4) of *E. coli* (DH5α) in the same field. The contrast was changed linearly.

Similar results were found in three independent experiments. **b,** Schematic of bacterial counting by microscopy imaging. **c,** Strain ATCC700926 stained by PI, illuminated by both fluorescence illumination and phase contrast. A threshold for background removal is shown in (**e**). Red arrows, microspheres observed by phase contrast illumination. Similar results were found in five independent experiments. **d,** Enlarged images (i-v) of (**c**). The colored lines are line profiles used for intensity measurements by ImageJ[11]; numbers, IDs of bright spots (*i.e.*, bacteria), shown in (**e**). **e,** Intensities (gray value) measured along the line profiles in (**d**). The dashed line is a threshold used for background removal (see **c**). Source data for (**e**) are provided as a Source Data file.

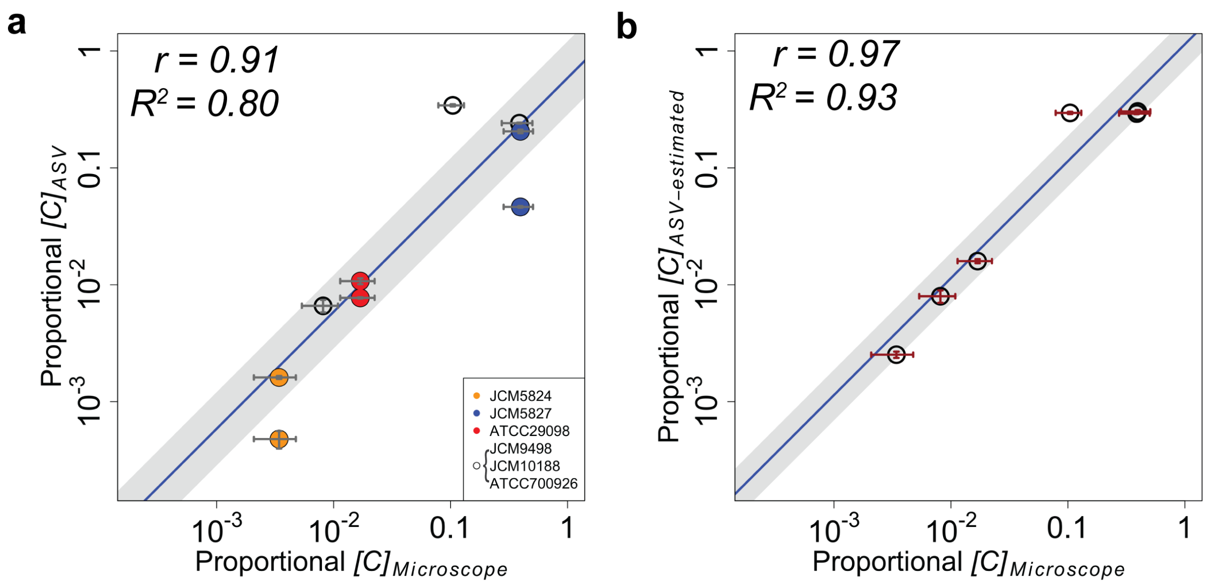

Supplementary Figure 8: Comparison between the proportional abundance of ASV and strains measured by microscopic imaging.

a. Same comparison as Fig. 2d, but only six strains with 16S rRNA gene copy numbers in their genome that were registered in the rrnDB database[13] were involved. Data are presented as mean values +/- SD (n = 2 for proportional $[C]_{ASV}$, n = 5 for proportional $[C]_{Microscope}$). b, Comparison of the estimated proportional cell abundances of ASVs (proportional $[C]_{ASV\text{-}estimated}$) with the proportional $[C]_{Microscope}$ measured by microscopic imaging. The proportional $[C]_{ASV\text{-}estimated}$ for each strain was calculated as follows: the abundances of multiple ASVs that were identical to the San sequences from the same strain were summed; the summed abundances were further normalized using the 16S rRNA gene copy numbers of the strains. Both proportional $[C]_{ASV\text{-}estimated}$ and proportional $[C]_{Microscope}$ were normalized to proportion again by the total abundance of these six strains. Data are presented as mean values +/- SD (n = 2 for $[C]_{ASV\text{-}estimated}$, n = 5 for proportional $[C]_{Microscope}$). Source data are provided as a Source Data file.

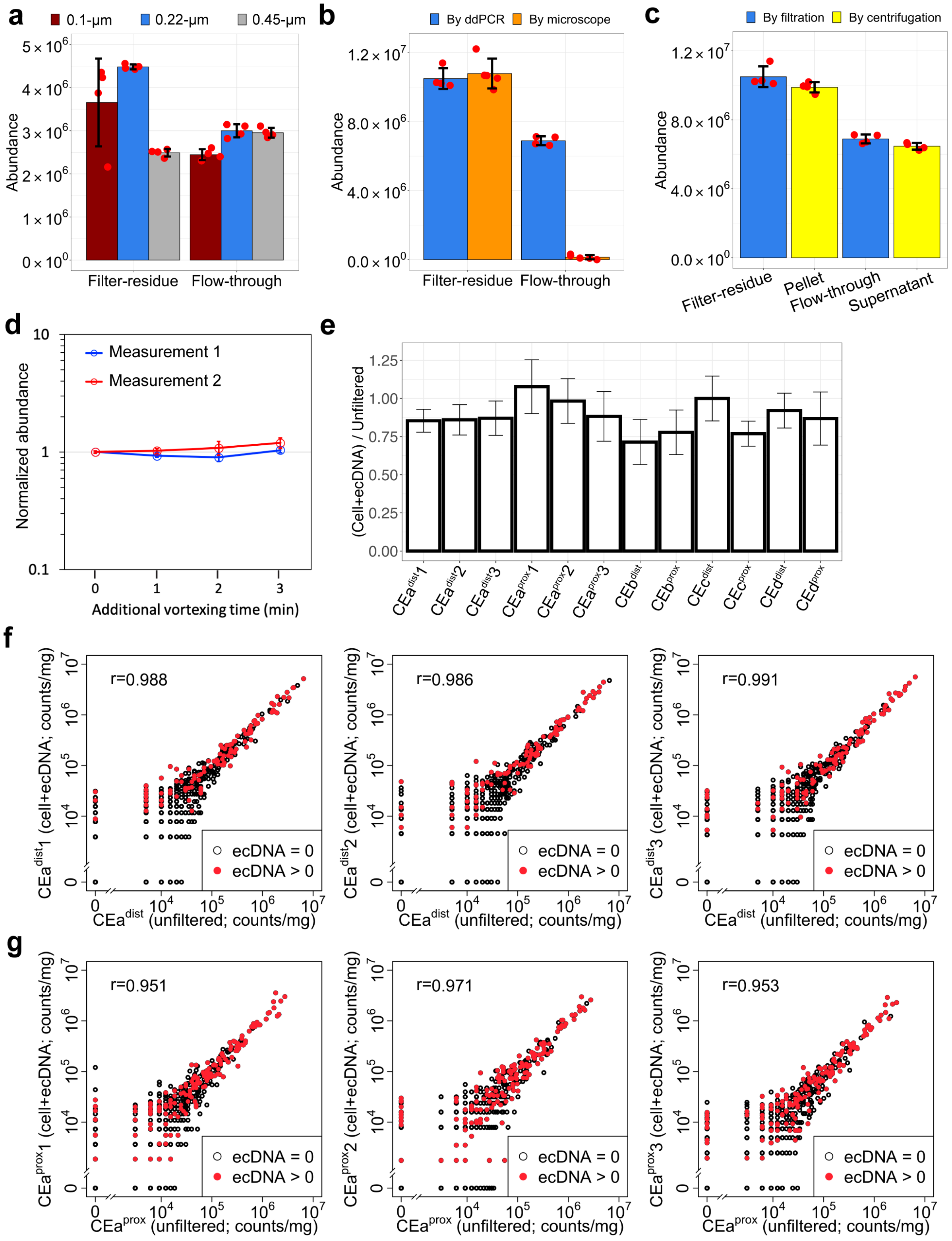

Supplementary Figure 9: Quality controls for the separation of ecDNA and cells.

a, Comparison of filtrations using 0.1-µm, 0.22-µm, and 0.45-µm pore size Ultrafree®-MC Centrifugal Filters. Filter-residue, sample that remained above the filter membrane; Abundance, total number of cells in filter-residue the filter and total number of DNA molecules in the flow-through as measured by ddPCR. Data are presented as mean values +/- SD (n = 4). **b,**

Comparison of the abundances of both cells and ecDNA after filtration between ddPCR and fluorescence microscopy imaging. Abundance, the same as in (**a**) for the ddPCR measurement and total number of bright spots for imaging measurements; data are presented as mean values +/- SD (n = 4 for ddPCR, n = 5 for microscope). **c,** Comparison of the separation of ecDNA and cells using filtration and centrifugation. Abundance, same as in (**a**); data are presented as mean values +/- SD (n = 4). **d,** Abundances of the ecDNA in cecal samples after vortexing as measured by ddPCR. Time zero, no additional vortexing after the vortex procedure in BarBIQ; additional vortexing time, additional time for vortexing after the vortex procedure in BarBIQ for the same sample; normalized abundance, number of fragmented DNA molecules normalized to that of time zero; error bars, standard deviations, n = 4. **e,** Ratio of the summed total abundance per unit weight (counts/mg) of the separated cells and ecDNAs to that of their unfiltered sample. Data are presented as mean values +/- propagated standard deviations which were calculated from the standard deviations of the total abundance of the cells (n = 5) and the ecDNA (n = 5). **f** and **g,** Comparison of each cOTU between the corresponding summed absolute abundance of cells and ecDNAs and the absolute abundance in their unfiltered-sample for the CEa^{dist} and CEa^{prox} samples, respectively. Red dots, cOTUs with detected ecDNA; black dots, cOTUs with undetected ecDNA. Three filtration replicates were compared. Source data for (**a-e**) are provided as a Source Data file. Source data for (**f**) and (**g**) are provided in Supplementary Data 8.

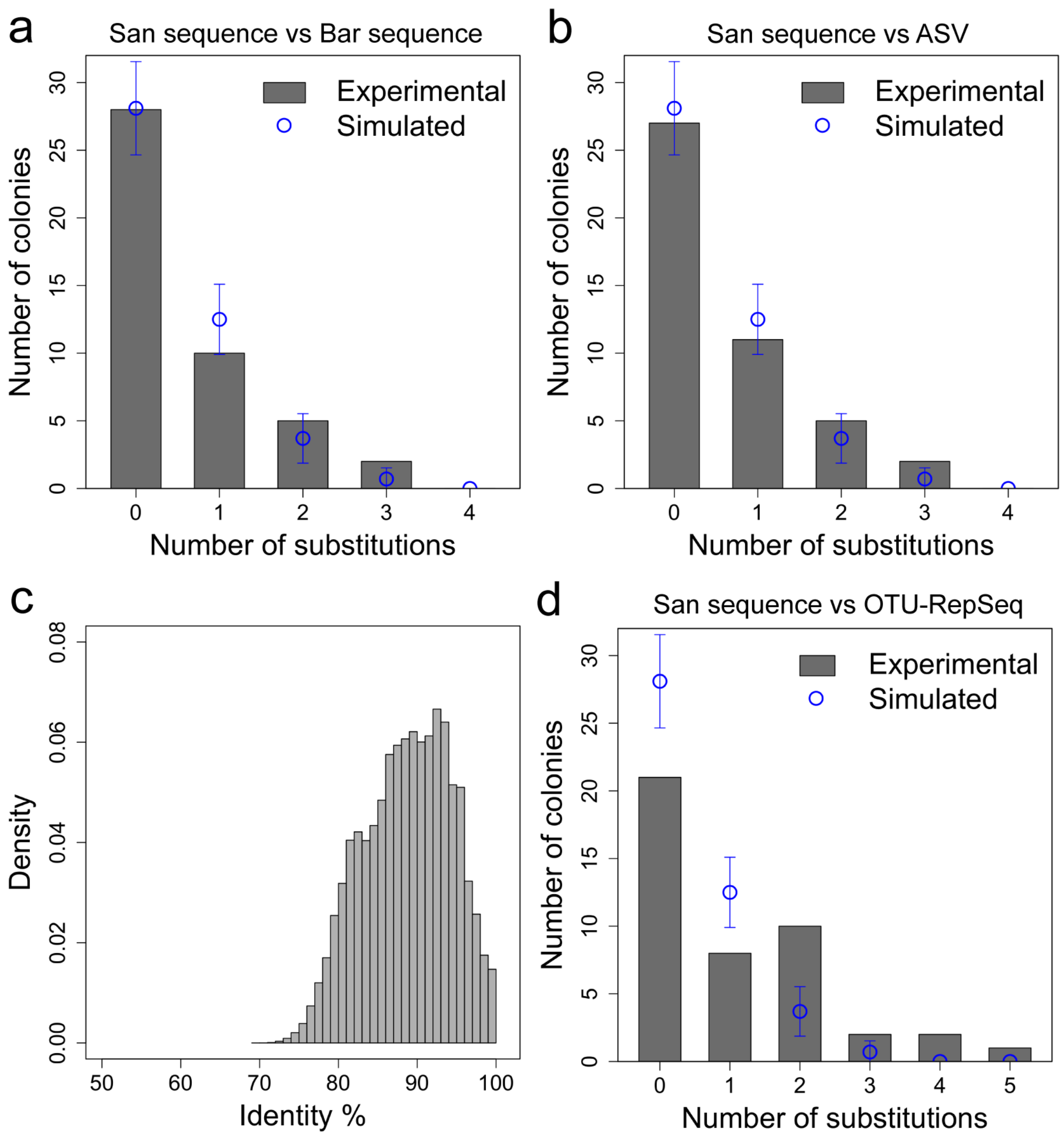

Supplementary Figure 10: Difference from San sequence to its closest Bar sequence, ASV, or OTU-RepSeq, and difference among 16S rRNA sequences in the database.

a, b, and **d,** Difference from the San sequence to its closest Bar sequence, ASV, or OTU-RepSeqs. The sequences detected from the cecal cell-sample VDd^{prox} were used. The San sequences were obtained as follows: in total, 48 colonies representing 48 amplified 16S rRNA gene molecules from randomly selected single bacteria were sequenced by Sanger sequencing for both strands (Methods). The regions between the sequences that were matched (at most two substitutions, one insertion, or one deletion were found) to primers 341F and 805R (Supplementary Table 3), *i.e.,* V3–V4, were used for further analyses. One colony showed very different sequences (edit distance: 36) from its two strands, which was not used for further analyses. The other 47 colonies were used for further analyses; 40 showed the same sequences from both strands, and seven showed small differences (one insertion or one deletion) between

their two strands at several consecutive bases. For these seven, the sequence from one strand that showed a clearer signal was used for further analyses for each colony. In comparison with Bar sequences (**a**), two colonies had one deletion from its closest Bar sequence. The number of deletions divided by both the total length of the San sequences of all colonies and the PCR cycles, 50 (the primers should have been used up after 50 cycles of PCR based on the concentration of the primers in droplet), were 2.1×10^{-6} deletion/base/doubling, which was comparable to the deletion rate (2.7×10^{-6} deletion/base/doubling) from a literature[14] for the same polymerase we used for the amplification, suggesting that the deletion of these two colonies may be generated by the amplification. The San sequences of the other 45 colonies all had zero or a few substitutions with their closest Bar sequence. The distribution of the substitutions in each colony (bars) was consistent with the distribution of the simulated number of substitutions for each colony based on both the length of its San sequence and the substitution error rate of the polymerase we used (2.28×10^{-5} substitutions/base/doubling, provided by the supplier, Thermo Fisher Scientific) (dots), suggesting that these substitutions may be generated by amplification as well. The simulated data are presented as mean values +/- SD (n = 10; ten independent simulations). In comparison with ASVs (**b**), similar results were found as Bar sequences (**a**). In comparison with OTU-RepSeqs (**d**), the distribution of the substitutions in each colony was not consistent with that of the simulated substitutions. Furthermore, one San sequence showed 18-insertion and two-substitution differences from its closest OTU-RepSeq, which cannot be explained by amplification errors. These results suggested that the OTU-RepSeqs were not consistent with the San sequences. **c,** Distribution of the identity between each pair of 10,000 16S rRNA genes that were randomly selected from the Silva database (v123.1). The identity was calculated based on the V3–V4 region. 99.99998% pairs showed > 70% identity. Source data for (**a**), (**b**), and (**d**) are provided in Supplementary Data 2. Source data for (**c**) are provided as a Source Data file.

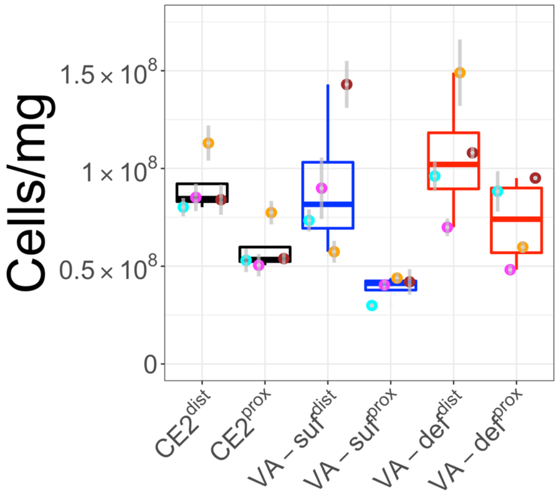

Supplementary Figure 11: Total cell abundance per unit weight of each cell-sample. CE2, CE2 nutrient group; VA-suf, VA-sufficient group; VA-def, VA-deficient group; dist and prox, locations; data are presented as mean values +/- SD for each dot (n = 4 for CE2, n = 5 for VA-suf and VA-def). Dots in the same color represent the samples from the same mouse (the samples in brown in the CE2 nutrient group were not sequenced). Boxes represent 25th to 75th percentiles (the interquartile range), horizontal black lines indicate medians, and whiskers show 1.5 times the interquartile range (n = 4). Source data are provided in Supplementary Data 8.

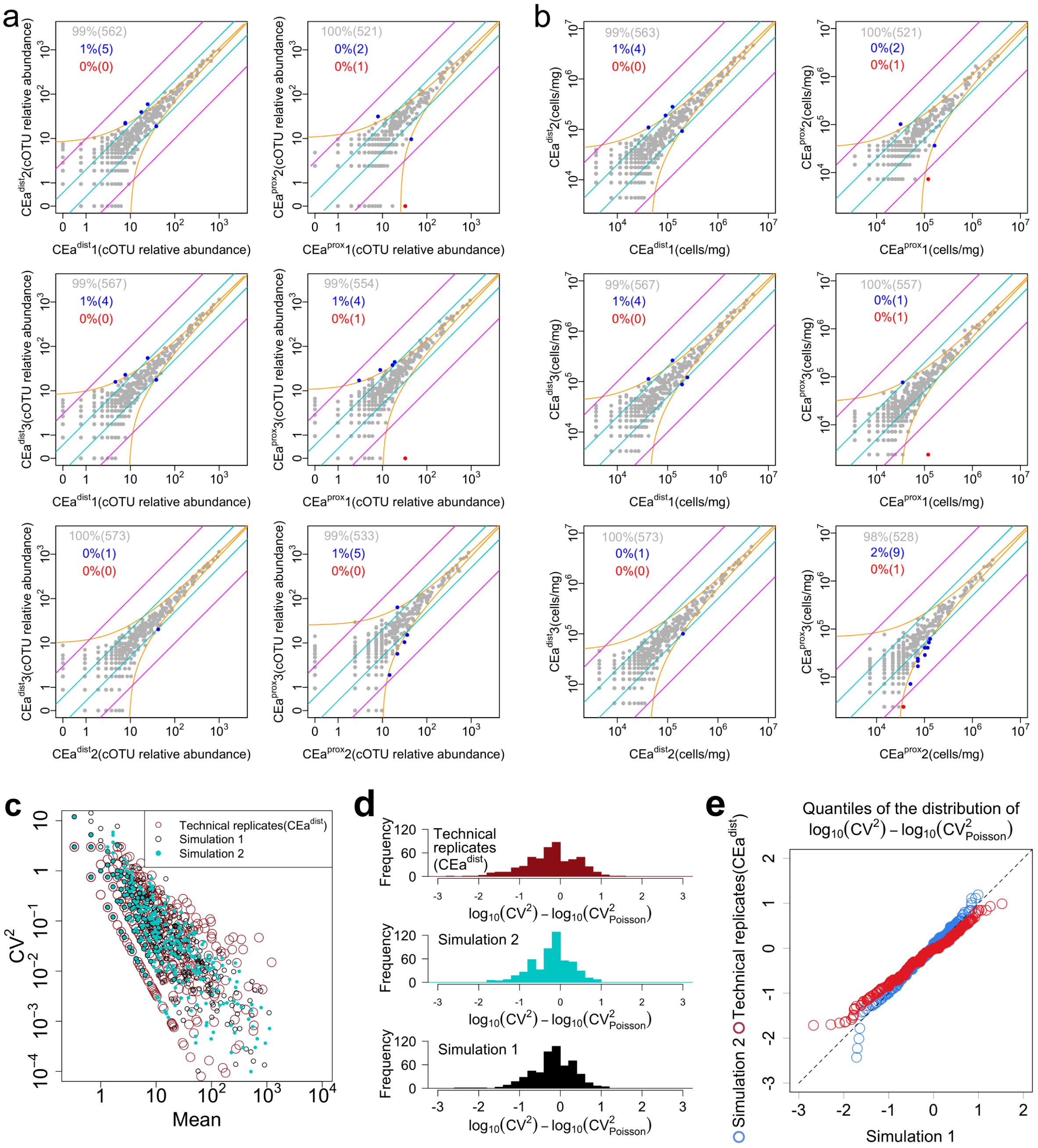

Supplementary Figure 12: Sampling noise for cOTU abundance in the BarBIQ measurement.

a, Comparison between technical replicates of CEa^{dist} and CEa^{prox} based on the relative cell abundances of each cOTU shown in the same format as Fig. 4a; CEa, mouse; dist and prox, locations; 1, 2, and 3, technical replicates. The top-left panel is shown as Fig. 4a. **b,** Comparison between technical replicates of CEa^{dist} and CEa^{prox} based on the absolute cell abundances per unit weight of each cOTU shown in the same format as Fig. 4a. **c,** CV^2 (square of the CV, coefficient of variation) of the counts in three technical replicates of CEa^{dist} for each cOTU as a function of the mean of the counts; simulated results (twice, 1 and 2) were obtained based on

Poisson distribution (see Methods). **d,** Distribution of $log_{10}(CV^2) - log_{10}(CV_{Poisson}^2)$. CV, CV of each cOTU; $CV_{Poisson}$, theoretical CV based on Poisson distribution (see Methods). **e,** A quantile-quantile plot[15] of the distributions of $log_{10}(CV^2) - log_{10}(CV_{Poisson}^2)$ between the measurement of CEa^{dist} and simulation 1 and between simulations 1 and 2. The distributions of $log_{10}(CV^2) - log_{10}(CV_{Poisson}^2)$ were comparable between the measurement and the simulation, suggesting that the noise for cOTU abundance measurements was mainly from Poisson distribution-based sampling. Source data for (**a**), (**c**), (**d**), and (**e**) are provided as a Source Data file. Source data for (**b**) are provided in Supplementary Data 8.

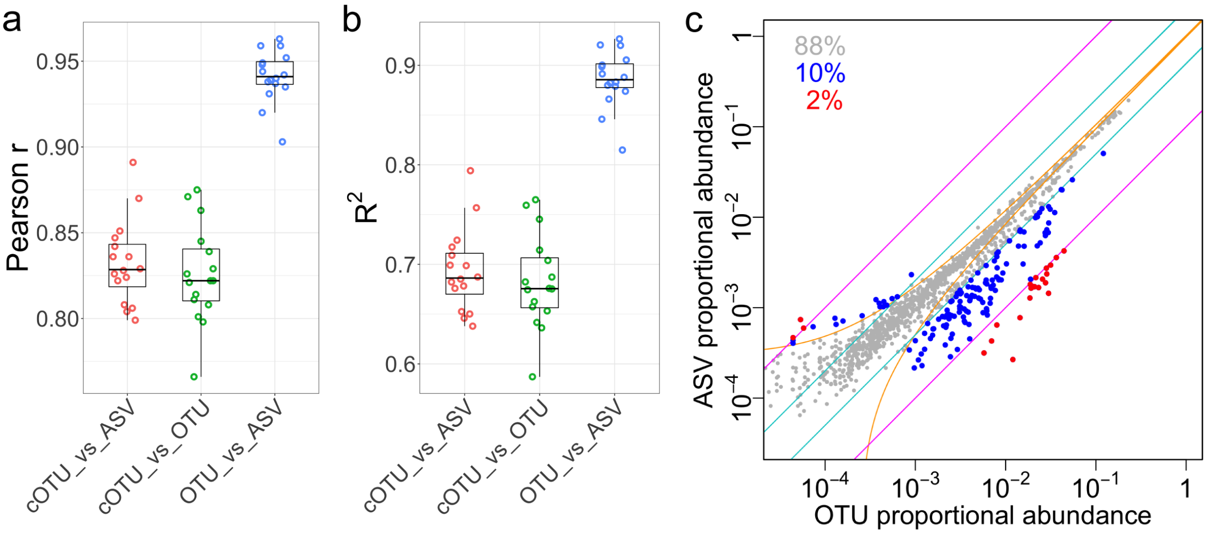

Supplementary Figure 13: Comparison of the quantification between different methods. **a** and **b,** Pearson *r* and coefficient of determination R^2 between the proportional abundances of the common pairs of cOTUs and ASVs (cOTU_vs_ASV), cOTUs and OTUs (cOTUs_vs_OTUs), and OTUs and ASVs (OTU_vs_ASV) for each cell-sample. Boxes represent 25th to 75th percentiles (the interquartile range), horizontal black lines indicate medians, and whiskers show 1.5 times the interquartile range (n = 16). **c,** Comparison between the proportional abundances of the common pairs of OTUs and ASVs with all 16 cell-samples shown in the same format as Fig. 4. Source data are provided as a Source Data file.

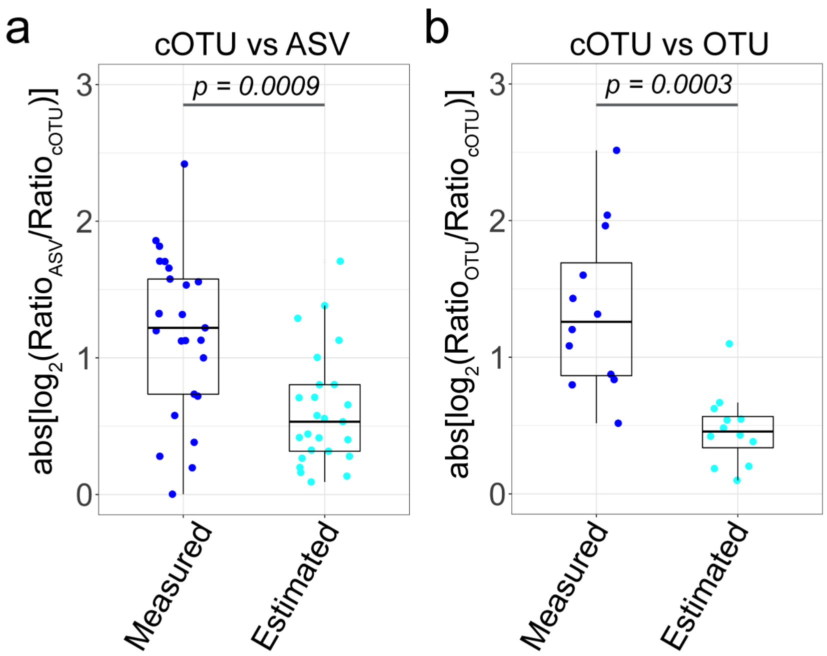

Supplementary Figure 14: Difference between cell abundance and 16S rRNA gene abundance for cecal cell-samples in the VA group.

a, Comparison of cOTUs and ASVs using five commonly detected cOTUs and ASVs, which have 16S rRNA gene copy numbers that are registered in the rrnDB database[13]. For "Measured", we first calculated the ratio of cell abundances between all possible pairs of cOTUs ($Ratio_{cOTU}$) and the ratio of 16S rRNA gene abundances between all possible pairs of ASVs ($Ratio_{ASV}$). Subsequently, we calculated a value, abs[log_2($Ratio_{ASV}$/$Ratio_{cOTU}$)], which represents the difference between the quantifications of cOTUs and ASVs. For "Estimated", we used the calculated $Ratio_{cOTU}$ and $Ratio_{ASV}$ above. Then, we normalized each $Ratio_{ASV}$ using their 16S rRNA gene copy numbers (*i.e.,* the $Ratio_{ASV}$ was calculated after the 16S rRNA gene abundances of the ASVs in the pair were divided by their 16S rRNA gene copy numbers respectively), and calculated abs[log_2($Ratio_{ASV}$/$Ratio_{cOTU}$)] using the normalized $Ratio_{ASV}$. The abs[log_2($Ratio_{ASV}$/$Ratio_{cOTU}$)] that had raw abundances of cOTUs and ASVs that were detected in sequencing were > 10 in each of 16 cell-samples were plotted. **b,** The same analysis as (**a**) for the three commonly detected pairs of cOTUs and OTUs whose 16S rRNA gene copy numbers were registered in the rrnDB database. For this analysis, zero of the y-axis means that the quantifications of the compared methods were consistent. Globally, both abs[log_2($Ratio_{ASV}$/$Ratio_{cOTU}$)] and abs[log_2($Ratio_{OTU}$/$Ratio_{cOTU}$)] were significantly decreased from "Measured" to "Estimated", which was consistent with the design principle of these methods as follows: BarBIQ measures cell abundance, and the ASV-based and OTU-based analyses measure 16S rRNA gene copies. Boxes represent 25th to 75th percentiles (the

interquartile range), horizontal black lines indicate medians, and whiskers show 1.5 times the interquartile range (n = 25 for cOTU vs ASV, n = 12 for cOTU vs OTU). *P* values were calculated by the Kruskal-Wallis rank sum test. Source data are provided as a Source Data file.

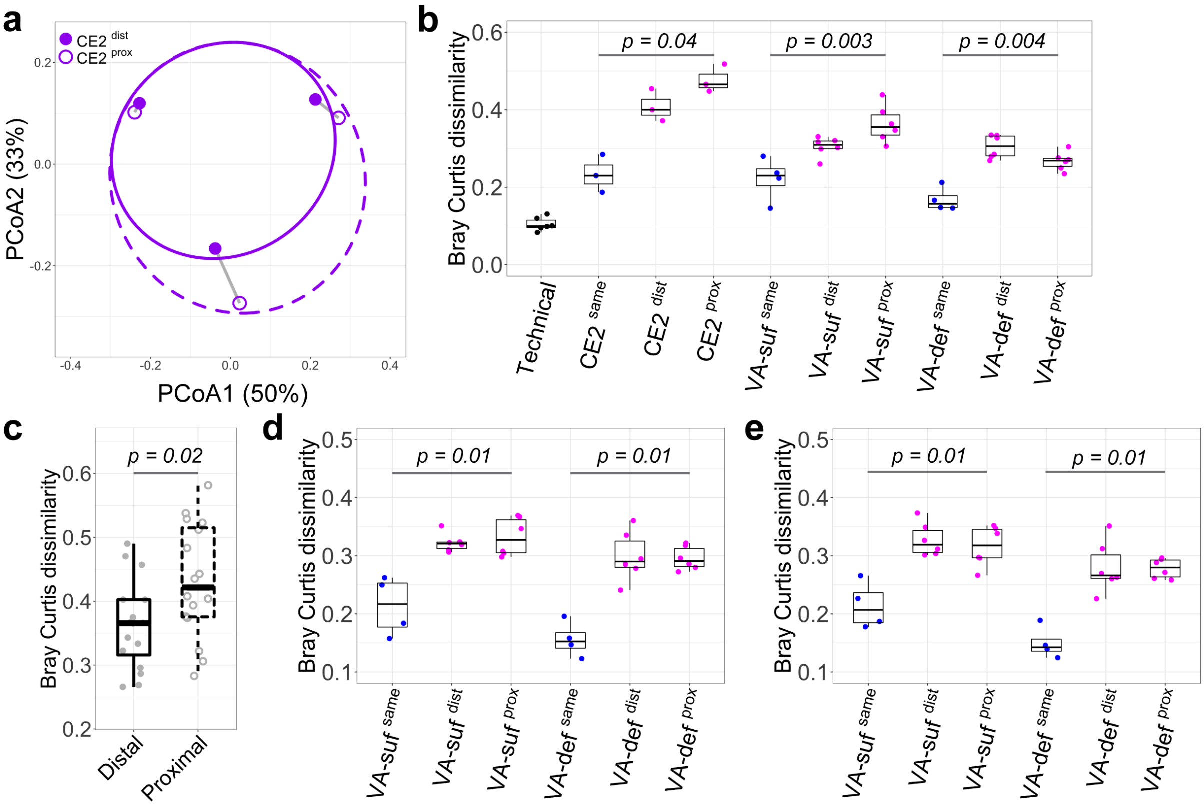

Supplementary Figure 15: Bray-Curtis dissimilarity.

a, Principal coordinates analysis (PCoA) of Bray-Curtis dissimilarities calculated using the relative cell abundances of cOTUs between each pair of cell-samples in the CE2 nutrient group. CE2, CE2 nutriment group; dist and prox, locations; gray line, linkage from the same mouse; circles, 95% confidence ellipses for each group. **b,** Quantitative comparison of Bray-Curtis dissimilarities in (**a**) and Fig. 5b. VA-suf, VA-sufficient group; VA-def, VA-deficient group; same, pairs of different locations from the same mouse; dist or prox, all possible pairs of samples from distal or proximal location in CE2, VA-suf, or VA-def; **c,** Quantitative comparison of Bray-Curtis dissimilarities based on absolute cell abundance per unit weight of cOTUs. Distal and Proximal, the same as Fig. 5c, e, and g. **d** and **e,** Quantitative comparison of Bray-Curtis dissimilarities in Fig. 5d and f, respectively. Labels, the same as (**b**). Boxes represent 25th to 75th percentiles (the interquartile range), horizontal black lines indicate medians, and whiskers show 1.5 times the interquartile range (n = 3 for $CE2^{same}$, $CE2^{dist}$, and $CE2^{prox}$; n = 4 for $VA\text{-}suf^{same}$, $VA\text{-}def^{same}$; n = 6 for Technical, $VA\text{-}suf^{dist}$, $VA\text{-}suf^{prox}$, $VA\text{-}def^{dist}$, and $VA\text{-}def^{prox}$; and n = 16 for Distal and Proximal). *P* values were calculated by the Kruskal-Wallis rank sum test. Source data are provided as a Source Data file.

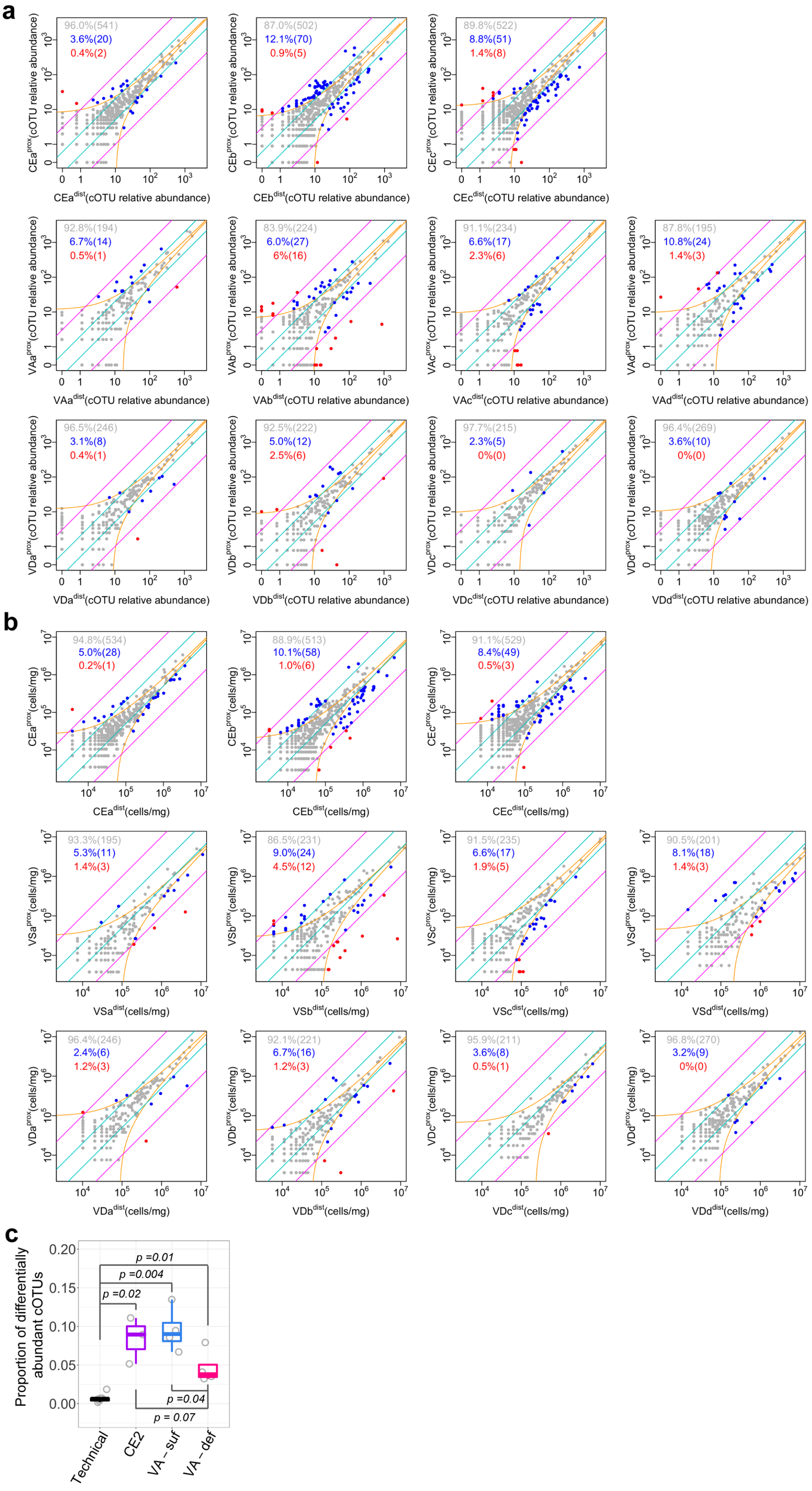

Supplementary Figure 16: Comparison of location-dependent cell abundance in each mouse.

a, Comparison between the relative cell abundances of cOTUs detected from the distal location and proximal location of each mouse, shown in the same format as Fig. 4a. CEa, CEb, CEc, VSa, VSb, VSc, VSd, VDa, VDb, VDc, and VDd, mouse; dist and prox, locations. Technical replicate 1 was used for CEa^{dist} and CEa^{prox}. The mouse VSa is shown as Fig. 6a. **b,** Comparison between the absolute cell abundances per unit weight of cOTUs detected from the distal location and proximal location of each mouse, shown in the same format as Fig. 4a. **c,** Proportion of location-dependent differentially abundant cOTUs (differences were larger than the sampling noise and 2-fold) in each mouse based on absolute cell abundances. Labels, same as Fig. 6b. *P* values were calculated by the Kruskal-Wallis rank sum test. Boxes represent 25th to 75th percentiles (the interquartile range), horizontal black lines indicate medians, and whiskers show 1.5 times the interquartile range (n = 6 for Technical, n = 3 for CE2, n = 4 for VA-suf and VA-def). Source data for (**a**) and (**c**) are provided as a Source Data file. Source data for (**b**) are provided in Supplementary Data 8.

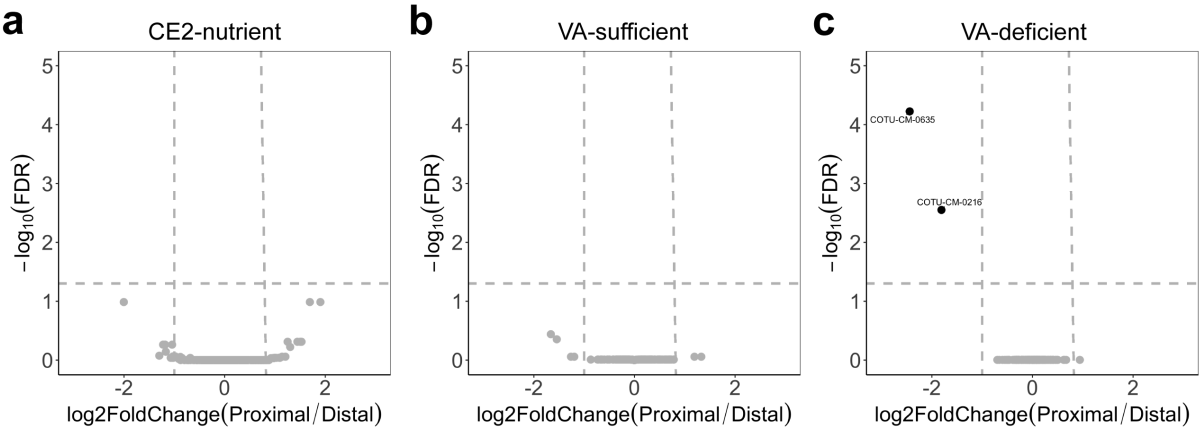

Supplementary Figure 17: Differential cell abundance of cOTUs between locations.

a, b, and **c,** Volcano plot showing the differential cell abundance of cOTUs between the proximal and distal locations in each group. FDR (false discovery rate) and log2FoldChange were calculated by DESeq2. Horizontal dotted line, FDR = 0.05; vertical dotted line, log2FoldChange = -1 or 1; labels, IDs of cOTUs. Source data are provided as a Source Data file.

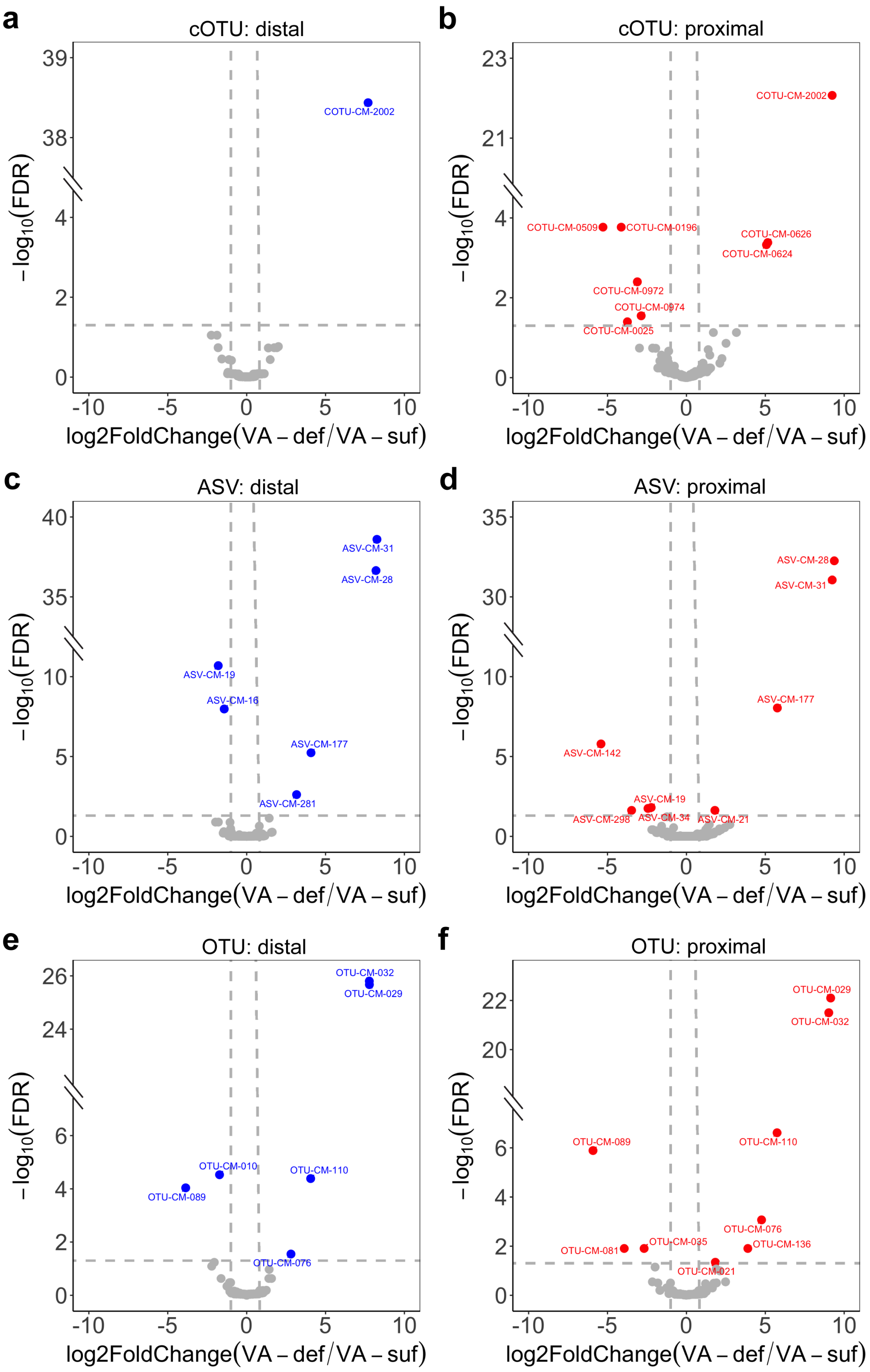

Supplementary Figure 18: Dietary vitamin A deficiency-based differential abundance analyses using cOTUs, ASVs, or OTUs.

a and **b,** Volcano plot showing the differential cell abundances of cOTUs between the VA-sufficient and VA-deficient groups for each location. The FDR and log2FoldChange were calculated by DESeq2. Horizontal dotted line, FDR = 0.05; vertical dotted line, log2FoldChange = -1 or 1; labels, IDs of cOTUs. **c** and **d,** Volcano plot showing the differential 16S rRNA gene abundances of ASVs between the VA-sufficient and VA-deficient groups for each location. Labels, IDs of ASVs. **e** and **f,** Volcano plot showing the differential 16S rRNA gene abundances of OTUs between the VA-sufficient and VA-deficient groups for each location. Labels, IDs of OTUs. Source data are provided as a Source Data file.

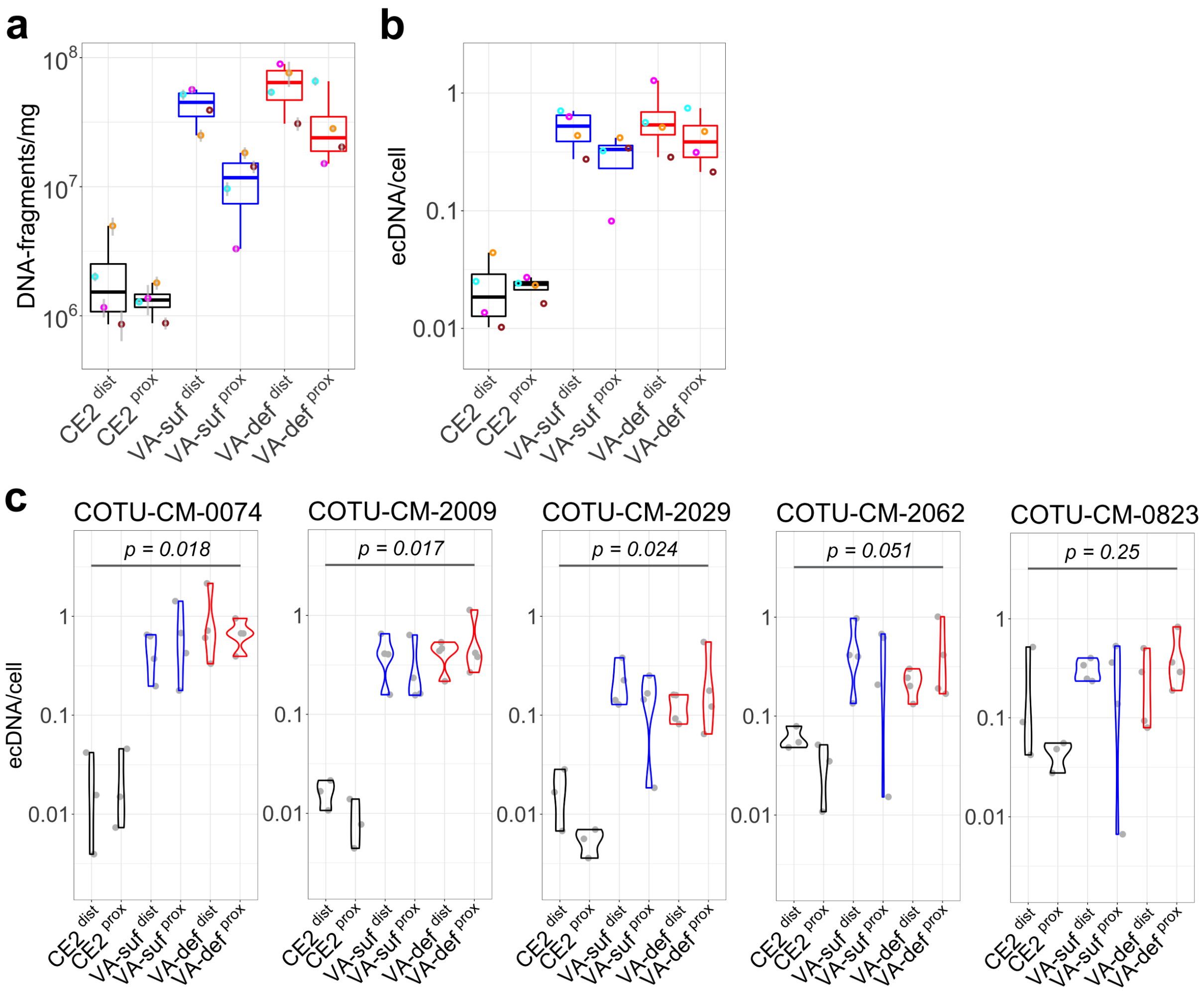

Supplementary Figure 19: Quantification of ecDNA in murine ceca.

a, Total DNA fragment abundance per unit weight of each ecDNA-sample. CE2, CE2 nutrient group; VA-suf, VA-sufficient group; VA-def, VA-deficient group; dist and prox, locations; data are presented as mean values +/- SD for each dot (n = 4 for CE2; n = 5 for VA-suf and VA-def). Dots in the same color represent the samples from the same mouse (the samples in brown in the CE2 nutrient group were not sequenced). **b,** The ratios of total DNA fragment abundances and total cell abundances per unit weight for each location and mouse. Boxes in (**a**) and (**b**) represent 25th to 75th percentiles (the interquartile range), horizontal black lines indicate medians, and whiskers show 1.5 times the interquartile range (n = 4). **c,** The ratios of DNA fragment abundances and cell abundances per unit weight of each location and mouse for five cOTUs that were commonly detected in all the cell- and ecDNA-samples. Source data for (**a**) are provided in Supplementary Data 8. Source data for (**b**) and (**c**) are provided as a Source Data file.

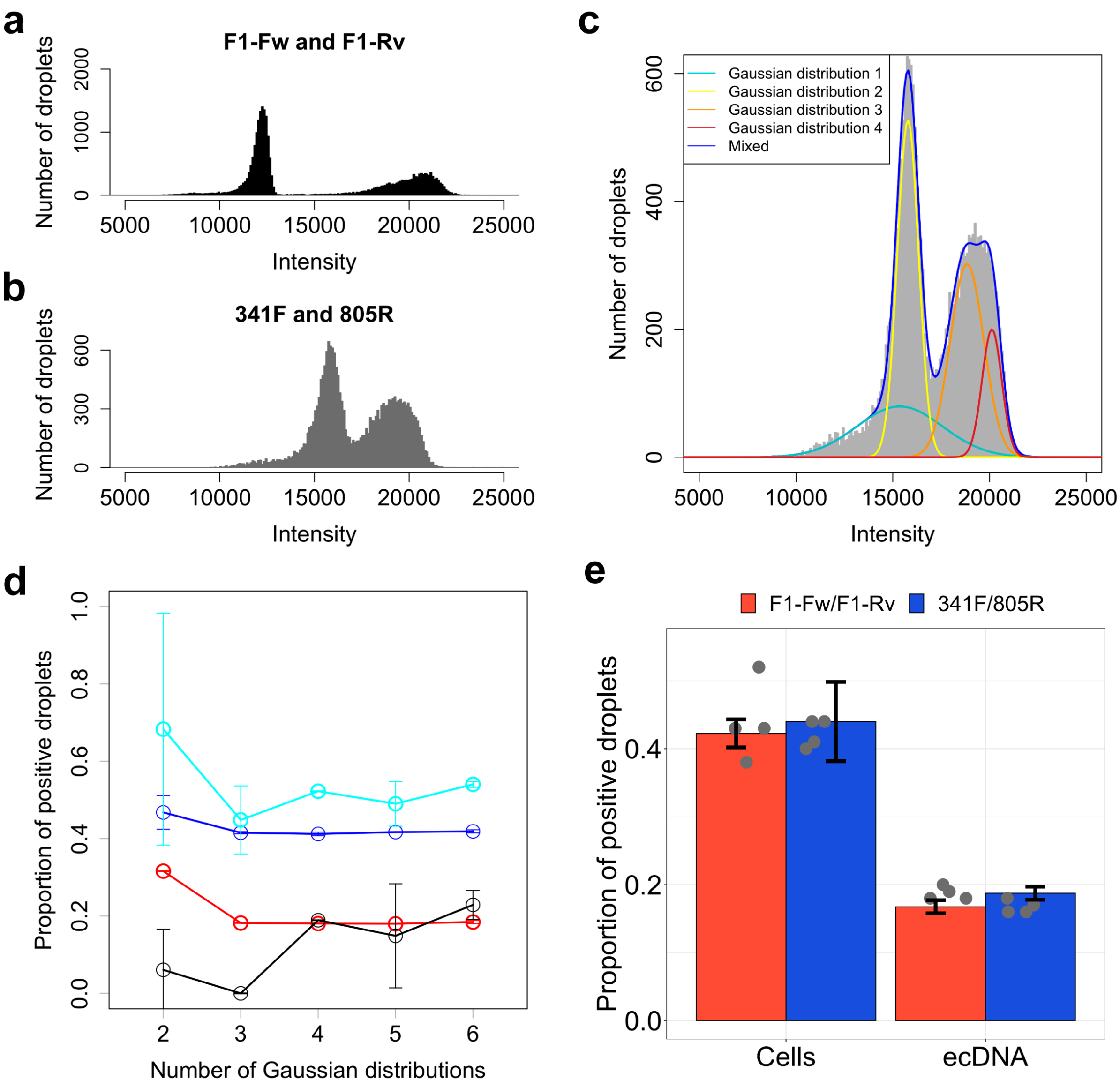

Supplementary Figure 20: Comparison between ddPCR measurements using primer sets F1-Fw/F1-Rv and 341F/805R for the same sample.

a, Distribution of the fluorescence intensities of the droplets measured by ddPCR for a cecal cell-sample (see main) using primers F1-Fw/F1-Rv. **b,** The same measurement as in (**a**) but using different primers (341F/805R). **c,** Fitting four Gaussian distributions to the fluorescence intensity distribution in (**b**). Mixed, sum of four Gaussian distributions. **d,** Calculated proportion of positive droplets based on fitting as a function of the number of fitted Gaussian distributions. Cyan, a cell-sample amplified by primers 341F/805R; blue, the same cell-sample as cyan but by primers F1-Fw/F1-Rv; red, an ecDNA-sample (see main) amplified by primers F1-Fw/F1-Rv; black, the same ecDNA-sample as red but amplified by primers 341F/805R; data are presented as mean values +/- SD (n =3; three independent fittings with different initial

seeds). **e,** A comparison of ddPCR measurements using primers F1-Fw/F1-Rv and using primers 341F/805R for the same sample; proportion of positive droplets calculated based on the fitting using 4 Gaussian distributions; Cells, the same cell-sample as in (**d**); ecDNA, the same ecDNA-sample as in (**d**); data are presented as mean values +/- SD (n = 4). Source data are provided as a Source Data file.

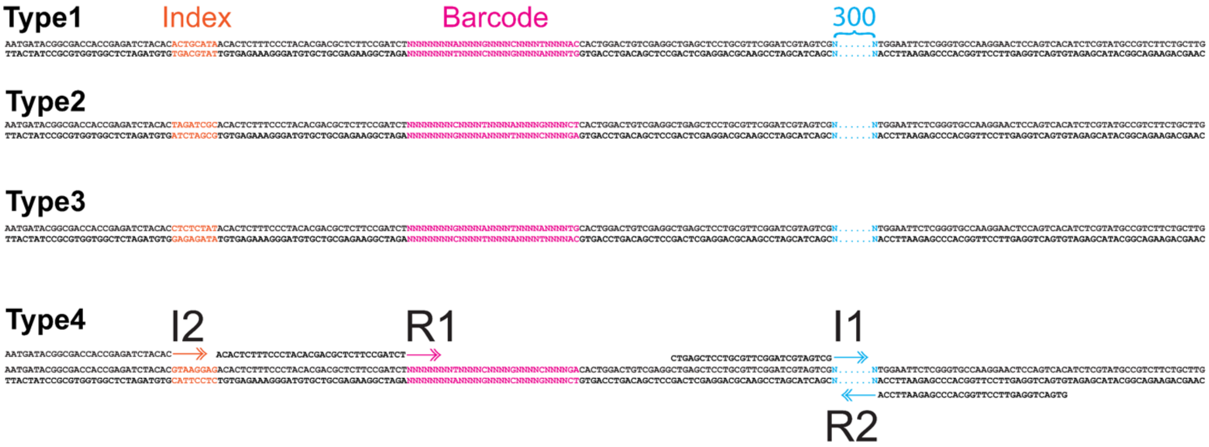

Supplementary Figure 21: Four types of DNA as the spike-in control.

Barcodes and indexes are different among the four types. R1, R2, I1, and I2, sequencing with the Illumina platform as in Supplementary Fig. 4.

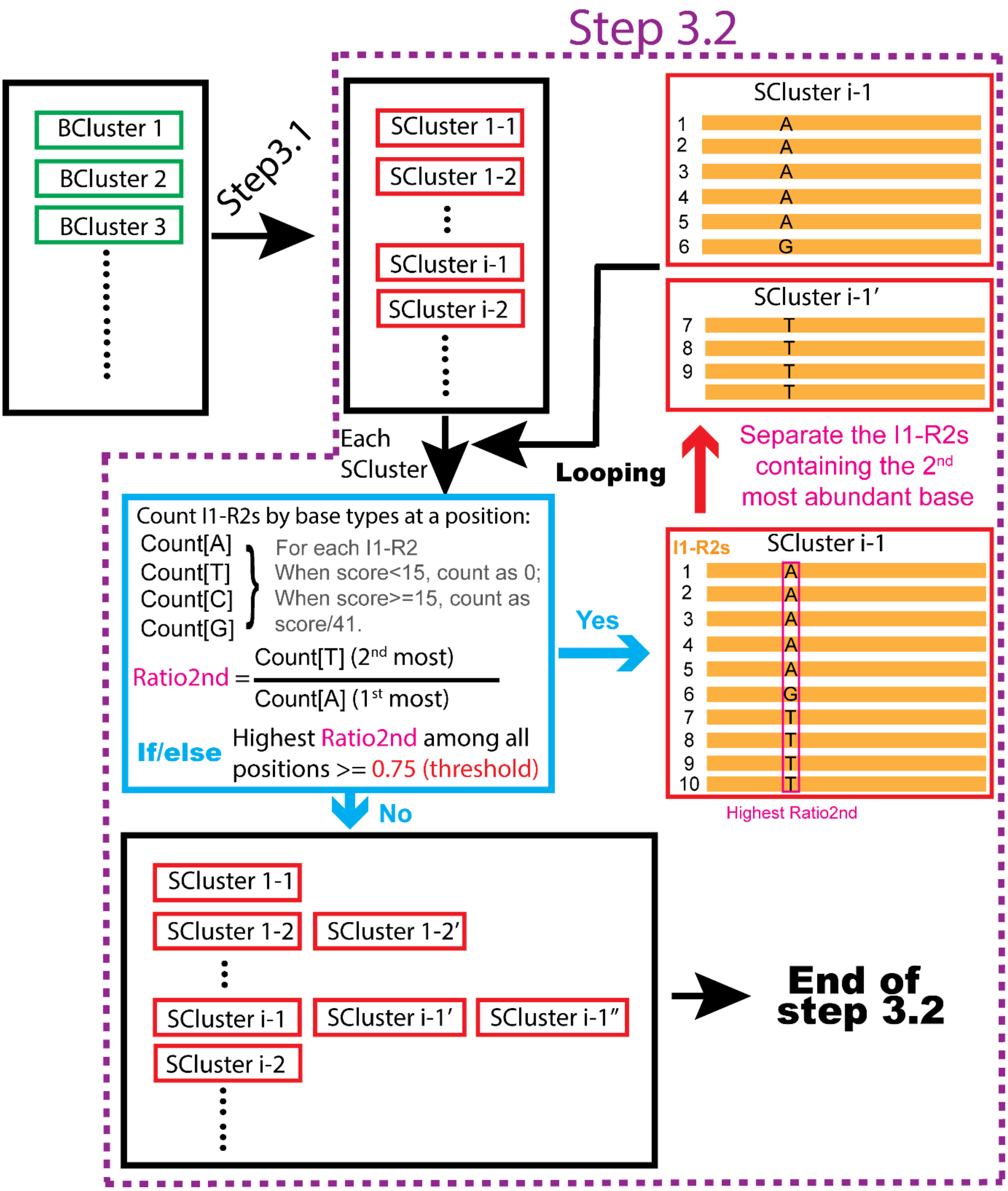

Supplementary Figure 22: Logic diagram of step 3.2.

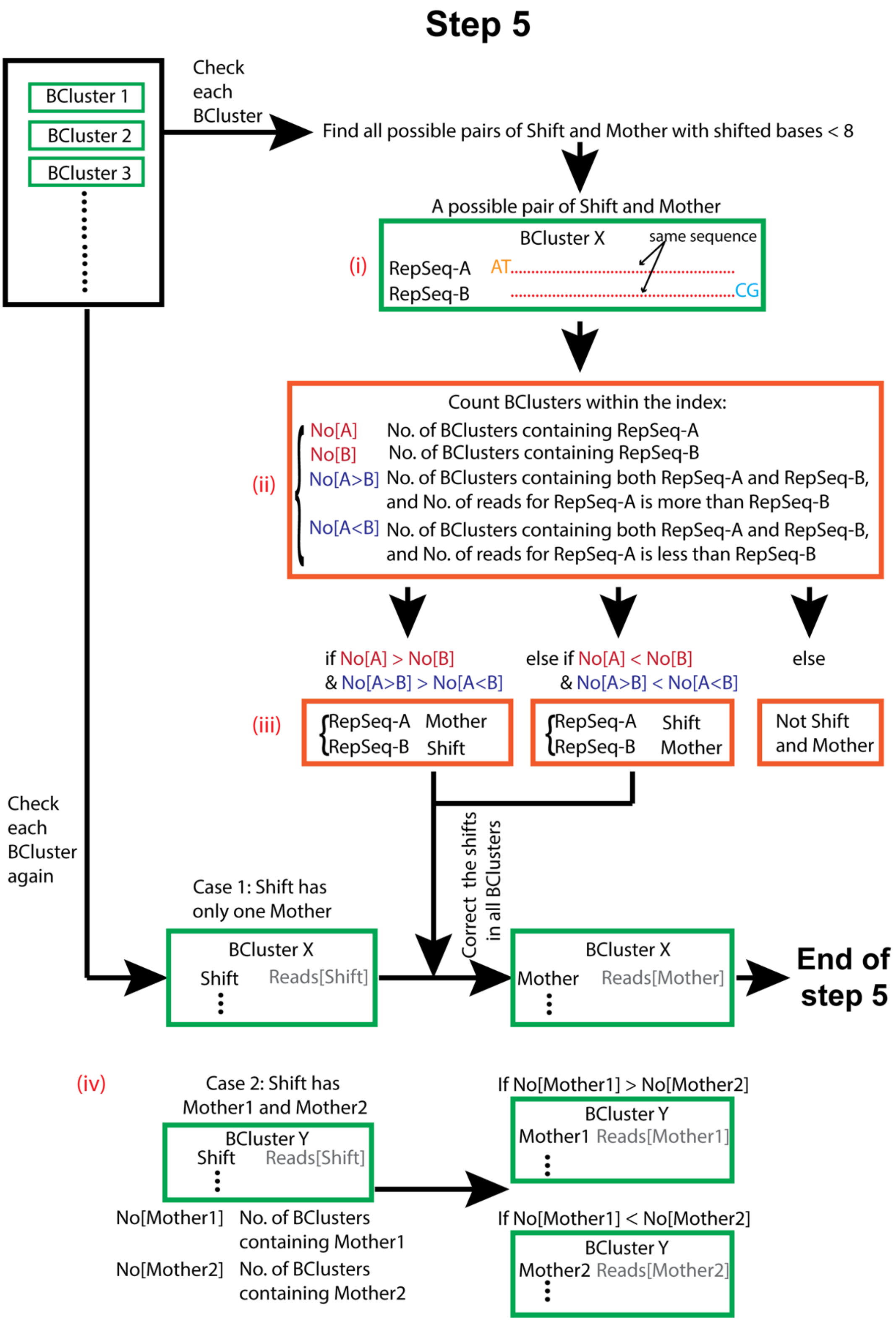

Supplementary Figure 23: Logic diagram of step 5.

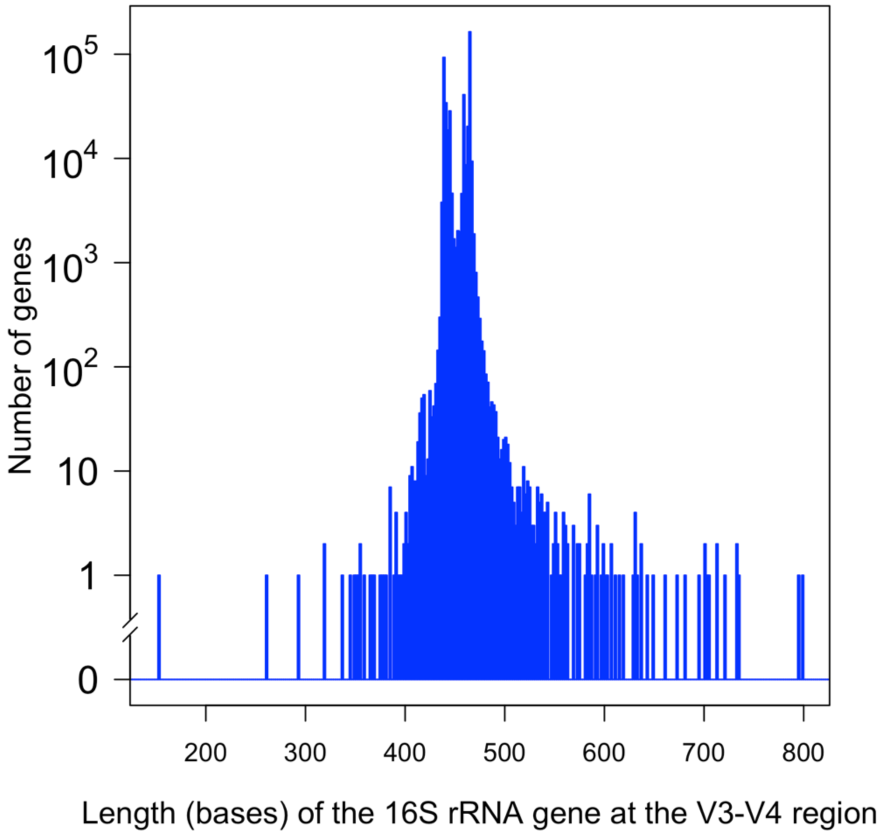

Supplementary Figure 24: Distribution of the lengths of 16S rRNA genes at the V3–V4 region.

The 16S rRNA genes registered in the Silva database (v123.1) and matched to primers 341F and 805R (Supplementary Table 3) are shown. The length is the number of bases from the first base matched with 341R to the last base matched with 805R. Source data are provided as a Source Data file.

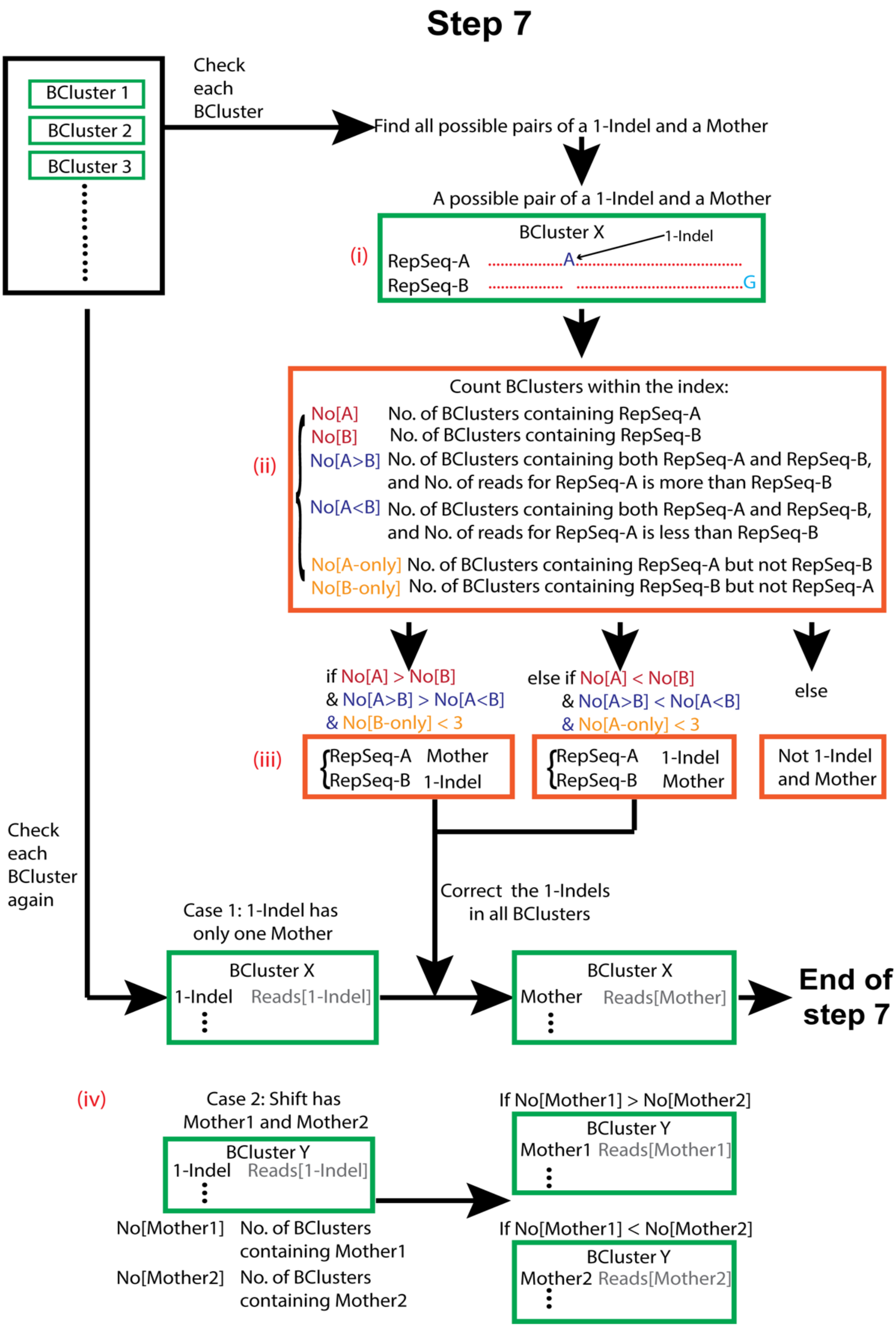

Supplementary Figure 25: Logic diagram of step 7.

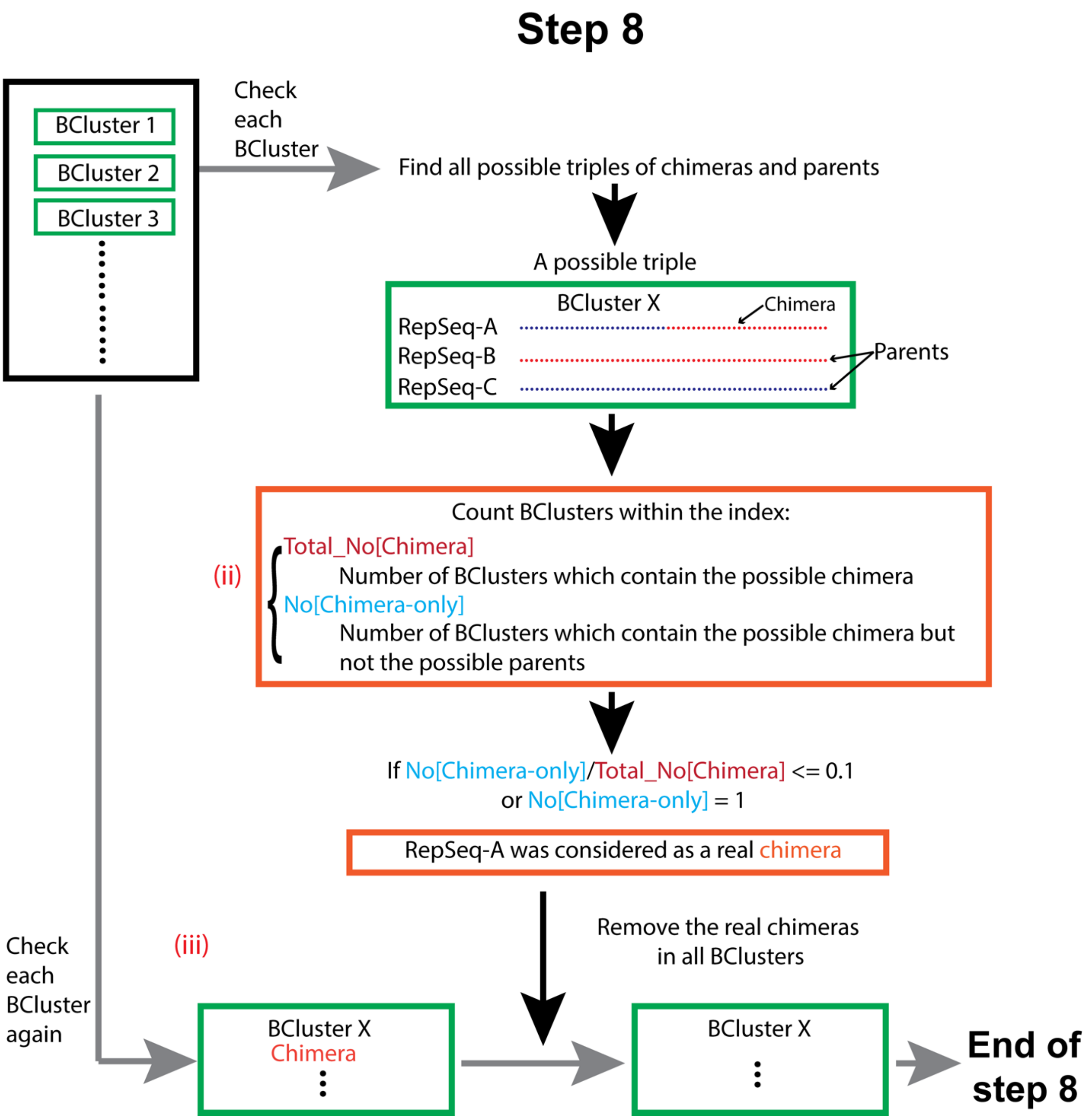

Supplementary Figure 26: Logic diagram of step 8.

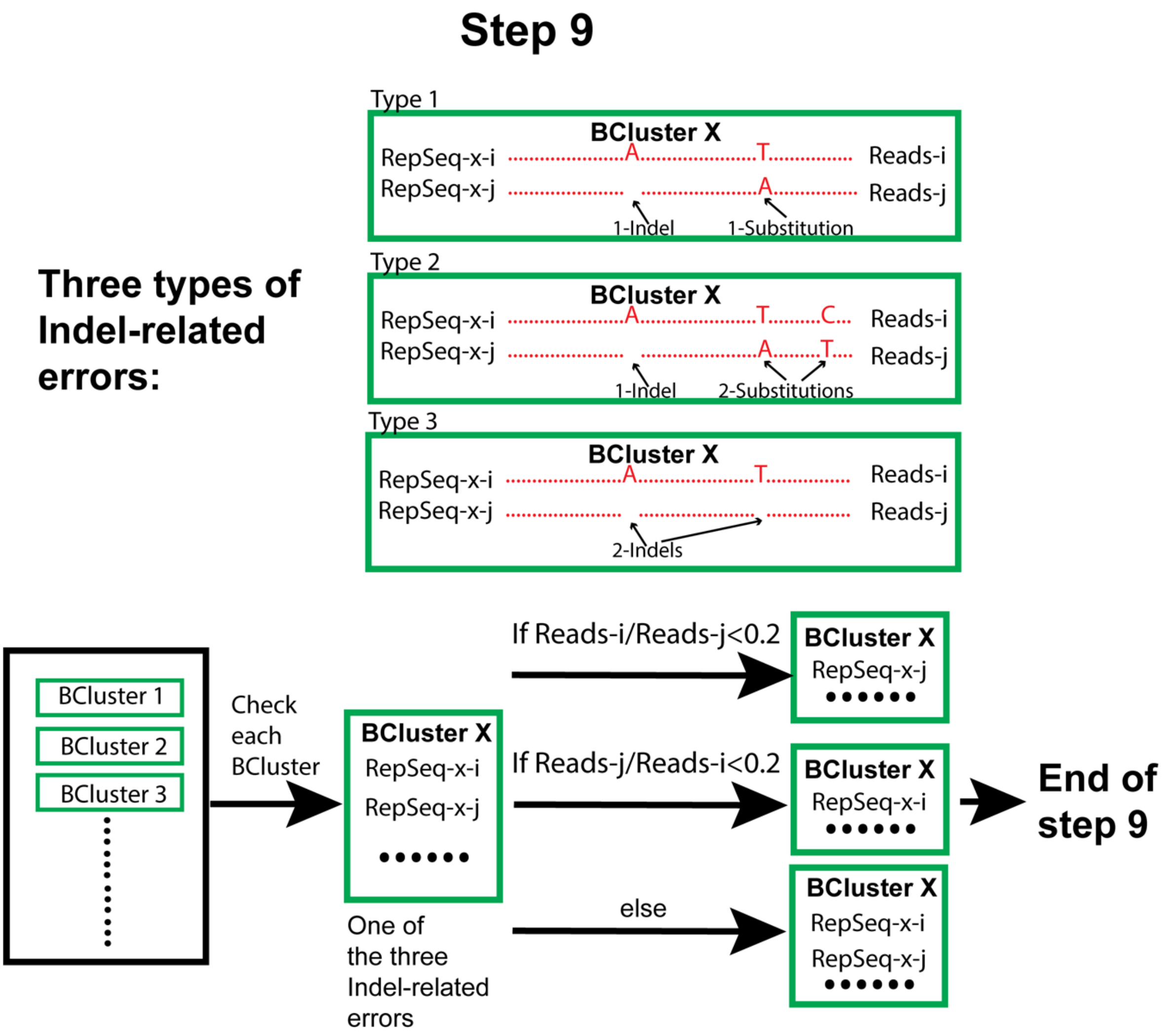

Supplementary Figure 27: Logic diagram of step 9.

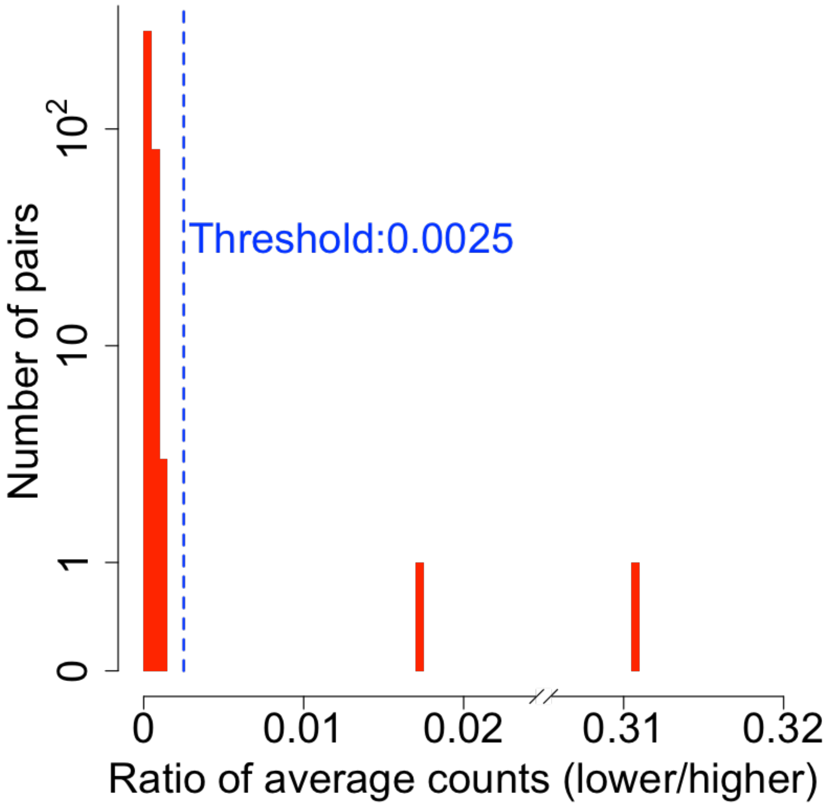

Supplementary Figure 28: Distribution of average count ratios between pairs of RepSeq types that had one substitution.

The average counts from three sampling replicates for each RepSeq type for the mock community are shown. Source data are provided as a Source Data file.

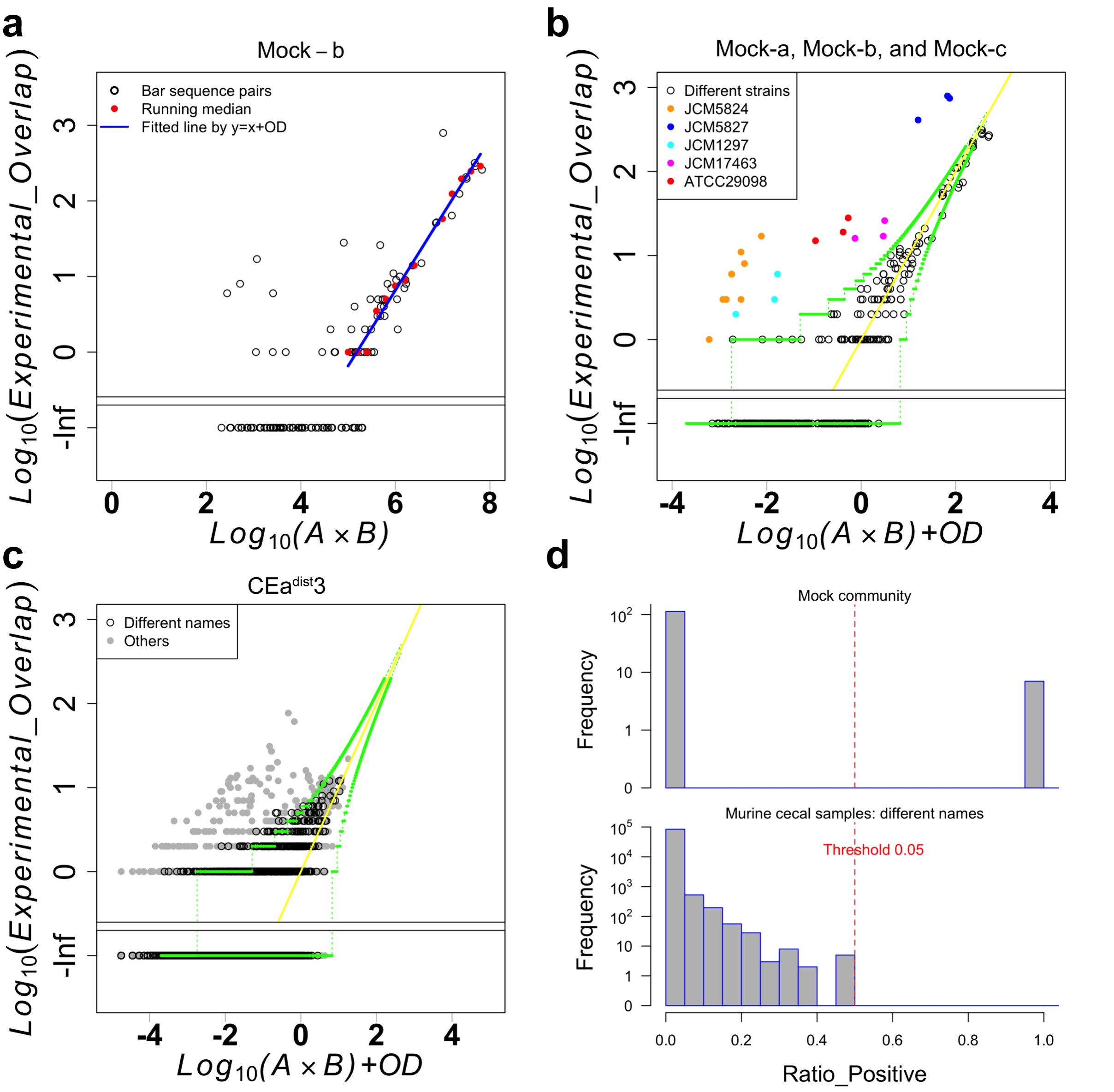

Supplementary Figure 29: Identifying multiple Bar sequences from the same bacterium. **a,** The *log₁₀(Experimental_Overlap)* against *log₁₀(A×B)*. Data from Mock-b are shown. Dots, all possible pairs of Bar sequences; *Experimental_Overlap*, *A*, and *B*, refer to the number of BClusters that contained both Bar sequence A and Bar sequence B, Bar sequence A, and Bar sequence B, respectively, for each pair; running medians, see Supplementary Note 2, data processing step 15. **b,** The *log₁₀(Experimental_Overlap)* against *log₁₀(A×B)+OD*. The results for Mock-a, Mock-b, and Mock-c are shown. Different strains, Bar-sequence pairs from different strains; JCM/ATCC<number>, Bar-sequence pairs from the given strain; green lines, 99.9% one-sided confidence intervals of *log₁₀(A×B)+OD* obtained by simulation; yellow line, x=y; OD, the estimated $log_{10}\left(\frac{\mu}{Droplets}\right)$ by fitting in (**a**) (see Supplementary Note 2, data processing step 15). Contaminated Bar sequences (see Supplementary Note 2, data processing

step 17) are not shown. **c,** The *log_{10}(Experimental_Overlap)* against *$log_{10}(A \times B)+OD$*. Result of the technical replicate 3 for the cell-sample at the distal location of the mouse CEa. Different names, Bar sequences for the pair mapped to different bacterial names in the Silva database (v138). **d,** Distributions of Ratio_Positive (see Supplementary Note 2, step 15). The results from the mock community sample or all cecal cell-samples. Different names, the same as in (**c**). Source data are provided as a Source Data file.

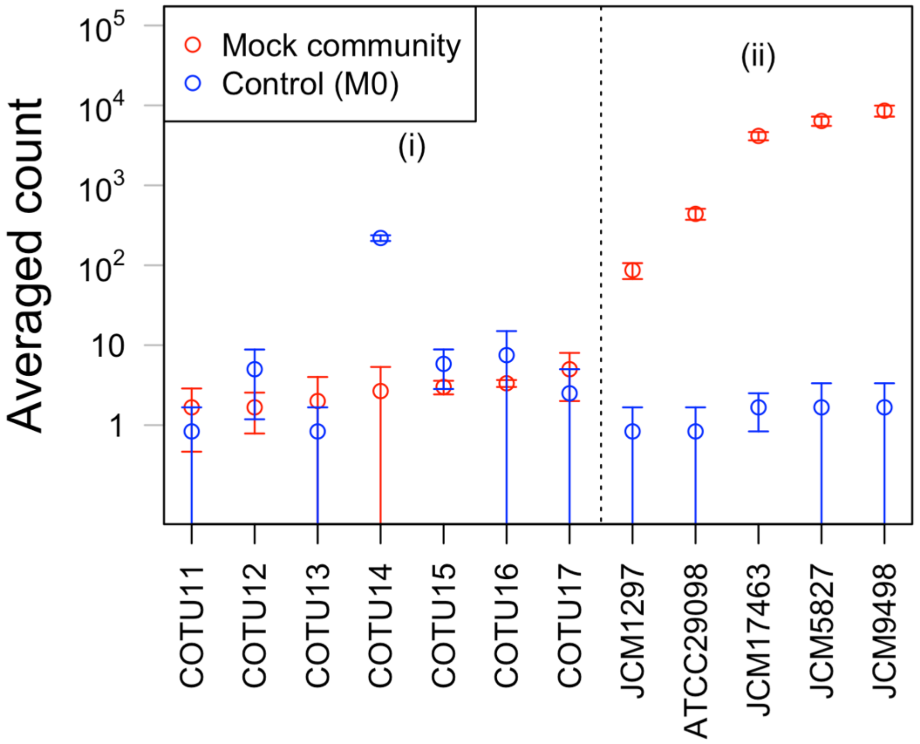

Supplementary Figure 30: Comparison of average cOTU counts between the mock community and the control M0.

JCM/ATCC<number>, Bar sequence(s) in cOTU matched to San sequence(s) of the given strain. COTU<number>, Bar sequence(s) in cOTUs not matched to any San sequence of the ten strains; i, ii, the two conditions (see Supplementary Note 2, step 17); data are presented as mean values +/- SEM (n = 3). Source data are provided as a Source Data file.

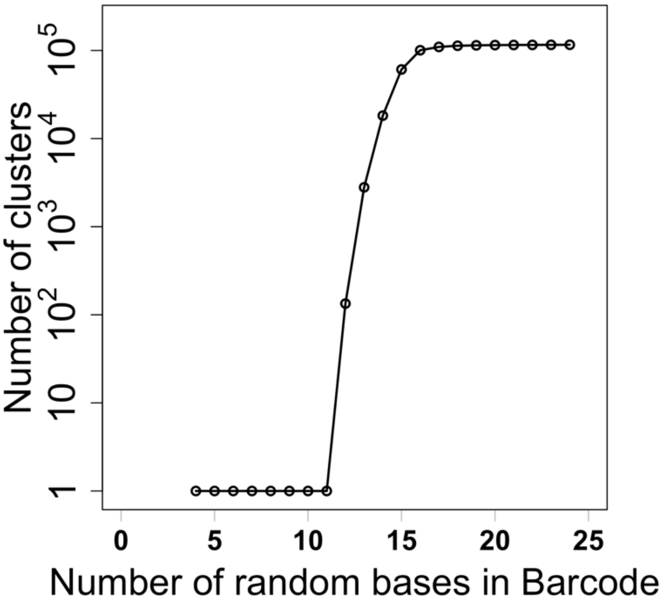

Supplementary Figure 31: Dependence of the number of clusters (unique barcodes) on the number of random bases designed in the barcodes.

The number of clusters and clusters of all samples, excluding the co-sequenced spike-in controls, in sequencing run 1 are shown. Source data are provided as a Source Data file.

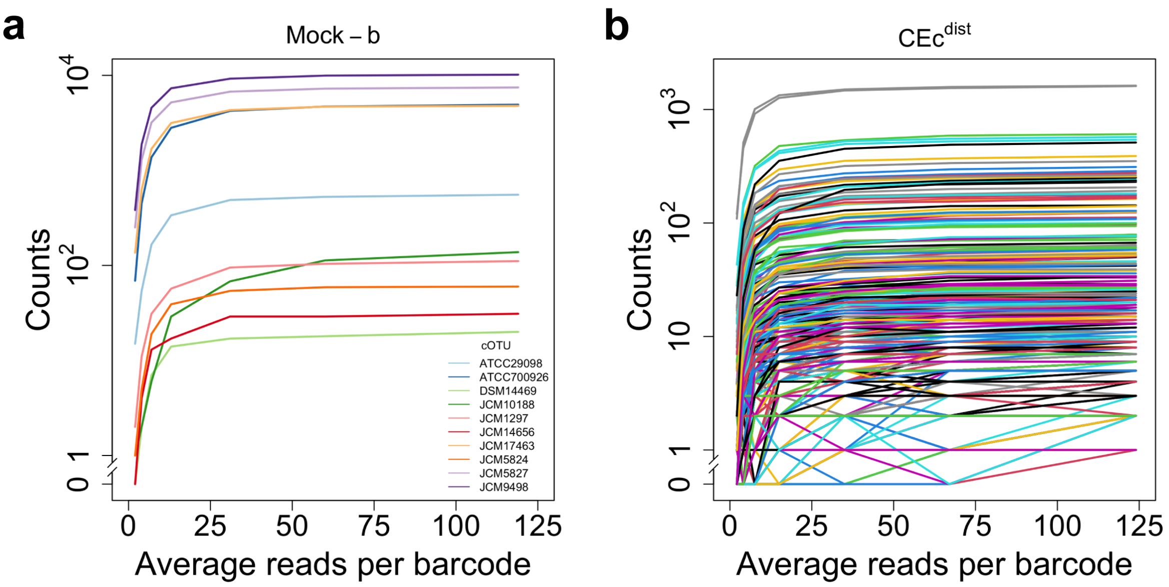

Supplementary Figure 32: Dependence of the counts of cOTUs on the average number of reads per unique barcode.

a, Data from Mock-b. The strain name for each cOTU is shown. **b,** Data from CEc^{dist}. Source data are provided as a Source Data file.

Supplementary Tables

Supplementary Table 1: Information for the ten cultured strains.

Bacterium name	Strain ID	Source	Culture medium	Culture condition	Wash	Storage	Abundance*	Phylum	Gram**
Collinsella aerofaciens	JCM10188	RIKEN BRC	GAM Agar	Anaerobic	-	##	$7.8\pm2.7\times10^{2}$	Actinobacteria	+
Bacteroides caccae	JCM9498	RIKEN BRC	GAM	Anaerobic	-	#	$3.7\pm1.1\times10^{4}$	Bacteroidetes	-
Bacteroides ovatus	JCM5824	RIKEN BRC	GAM	Anaerobic	-	#	$3.3\pm1.3\times10^{2}$	Bacteroidetes	-
Bacteroides thetaiotaomicron	JCM5827	RIKEN BRC	GAM	Anaerobic	-	#	$3.8\pm1.1\times10^{4}$	Bacteroidetes	-
Blautia hydrogenotrophica	JCM14656	RIKEN BRC	GAM	Anaerobic	PBS	##	$1.3\pm0.5\times10^{2}$	Firmicutes	+
Clostridium symbiosum	JCM1297	RIKEN BRC	GAM	Anaerobic	-	#	$5.5\pm2.3\times10^{2}$	Firmicutes	+
Agathobacter rectalis (Eubacterium rectale)	JCM17463	RIKEN BRC	GAM	Anaerobic	-	#	$7.2\pm2.9\times10^{3}$	Firmicutes	+
Marvinbryantia formatexigens	DSM14469	DSMZ	PYG	Anaerobic	PBS	##	$1.6\pm0.7\times10^{2}$	Firmicutes	+
Desulfovibrio piger	ATCC29098	ATCC	ATCC medium 1249	Anaerobic	-	#	$1.6\pm0.5\times10^{3}$	Proteobacteria	-
Escherichia coli	ATCC700926	ATCC	LB	Aerobic	-	#	$1.0\pm0.2\times10^{4}$	Proteobacteria	-

*Designed cell abundance per unit volume (mean ± s.d., n = 5, cells/µl) in the mock community according to the microscopic imaging measurements for each strain.

** “+”: Gram-positive; “-”: Gram-negative.

Stored in culture medium with 10% glycerol at −80 °C.

Stored in phosphate-buffered saline (PBS) at −80 °C.

GAM, Gifu Anaerobic Medium (Nissui).

GAM Agar, Modified GAM Agar (Nissui).

LB, Luria-Bertani (Nacalai Tesque).

PYG, Peptone Yeast Glucose, DSMZ medium 104.

ATCC medium 1249, Modified Baar's medium for sulphate reducers.

Supplementary Table 2: Diet formulation for vitamin A-sufficient and vitamin A-deficient diets.

		Vitamin A-sufficient diet	Vitamin A-deficient diet
Product # (Formulated by Research Diets, Inc.)		A18041301	A21022401
Nutritional Class	Ingredient	g/Kg	g/Kg
L-Amino Acids ("protein")	L-Arginine	6.0	6.0
	L-Histidine-HCl-H2O	4.6	4.6
	L-Isoleucine	7.6	7.6
	L-Leucine	16.0	16.0
	L-Lysine-HCl	13.3	13.3
	L-Methionine	5.1	5.1
	L-Phenylalanine	8.5	8.5
	L-Threonine	7.2	7.2
	L-Tryptophan	2.1	2.1
	L-Valine	9.4	9.4
	L-Alanine	5.1	5.1
	L-Asparagine-H2O	7.1	7.1
	L-Aspartic Acid	5.1	5.1
	L-Cystine	4.3	4.3
	L-Glutamic Acid	21.0	21.0
	L-Glutamine	17.4	17.4
	Glycine	3.1	3.1
	L-Proline	17.9	17.9
	L-Serine	10.1	10.1
	L-Tyrosine	9.3	9.3
Carbohydrate	Corn Starch	404.2	404.2
	Maltodextrin 10	134.2	134.2
	Sucrose	108.9	108.9
	Cellulose	50.8	50.8
Fat	Soybean Oil	71.2	71.2
Micronutrients	t-butylhydroquinone	0.014	0.014
	Mineral Mix S10022C	3.6	3.6
	Calcium Carbonate	7.5	7.5
	Potassium Citrate, 1 H2O	2.5	2.5
	Potassium Phosphate, Monobasic	7.0	7.0
	Calcium Phosphate, dibasic	7.1	7.1
	Sodium Chloride	2.6	2.6
	Sodium Bicarbonate	7.6	7.6
	**Vitamin Mix V10037***	**10.2**	**0.0**
	**Vitamin Mix V13002, No added Vit A**	**0.0**	**10.2**
	Choline Bitrartrate	2.5	2.5

* The added vitamin A level in the vitamin A-sufficient diet A18041301 is 4,000 IU/Kg.

Supplementary Table 3: Sequences of primers and barcodes used in all experiments.

Primers	Sequences
341F	5'-CCTACGGGNGGCWGCAG-3'
805R	5'-GACTACHVGGGTATCTAATCC-3'
P7-R2P-341F	5'-CAAGCAGAAGACGGCATACGAGATGTGACTGGAGTTCCTTGGCACCCGAG AATTCCACCTACGGGNGGCWGCAG-3'
Biotin-Link-805R	5'-/5Biosg/GCTCCTGCGTTCGGATCGTAGTCGGAC/iBiodT/ACHVGGGTATCTAA TCC-3'
Biotin-Link-barcode-F	5'-/5Biosg/CGACTACGATCCGAACGCAGGAGCTCAGCC/iBiodT/CGACAGTCCAG TG-3'
P5-index-R1P-barcode-R	5'-AATGATACGGCGACCACCGAGATCTACACXXXXXXXXACACTCTTTCCCTA CACGACGCTCTTCCGATCT-3' (X: Index, see Supplementary Table 5)
NoBiotin-Link-barcode-F	5'-CGACTACGATCCGAACGCAGGAGCTCAGCCTCGACAGTCCAGTG-3'
F1-Fw	5'-AGRGTTTGATYMTGGCTCAG-3'
F1-Rv	5'-CTGGCACGDAGTTAGCC-3'
F1-full-Fw	5'-CAAGCAGAAGACGGCATACGAGATGTGACTGGAGTTCCTTGGCACCCGAG AATTCCAAGRGTTTGATYMTGGCTCAG-3'
F3-full-Rv	5'-GCTCCTGCGTTCGGATCGTAGTCG TACGGYTACCTTGTTACGACTT-3'
T7-Promoter	5'-TAATACGACTCACTATAG-3'
SP6-Promoter	5'-ATTTAGGTGACACTATAG-3'
F2-Rv	5'-CTTGTGCGGGCCCCCGTCAATTC-3'
Barcode-1	5'-TCAGCCTCGACAGTCCAGTGACNNNNTNNNNGNNNNANNNNCNNNNNNNN AGATCGGAAGAGCGTCGTGTAGGGAAAGAGTGT-3'
Barcode-2	5'-TCAGCCTCGACAGTCCAGTGTGNNNNANNNNCNNNNTNNNNGNNNNNNNN AGATCGGAAGAGCGTCGTGTAGGGAAAGAGTGT-3'
Barcode-3	5'-TCAGCCTCGACAGTCCAGTGGANNNNCNNNNANNNNGNNNNTNNNNNNNN AGATCGGAAGAGCGTCGTGTAGGGAAAGAGTGT-3'
Barcode-4	5'-TCAGCCTCGACAGTCCAGTGCTNNNNGNNNNTNNNNCNNNNANNNNNNNN AGATCGGAAGAGCGTCGTGTAGGGAAAGAGTGT-3'
P1_qPCR_Fw	5'-AATGATACGGCGCACCACCGA-3'
P2_qPCR_Rv	5'-CAAGCAGAAGACGGCATACGA-3'
I1_primer	5'-CTGAGCTCCTGCGTTCGGATCGTAGTCG-3'
CONV-341F	5'-TCGTCGGCAGCGTCAGATGTGTATAAGAGACAGCCTACGGGNGGCWGCA G-3'
CONV-805R	5'-GTCTCGTGGGCTCGGAGATGTGTATAAGAGACAGGACTACHVGGGTATCTA ATCC-3'

Supplementary Table 4: Perl modules or R packages used in the data analysis.

Name	Category	Citations
ape	R package	Paradis E. & Schliep K. 2019. ape 5.0: an environment for modern phylogenetics and evolutionary analyses in R. Bioinformatics, 35, 3, 526–528.
dplyr	R package	Hadley Wickham, Romain Fran<U+00E7>ois, Lionel Henry and Kirill M<U+00FC>ller (2018). dplyr: A Grammar of Data Manipulation. R package version 0.7.6. https://CRAN.R-project.org/package=dplyr
ggplot2	R package	H. Wickham. ggplot2: Elegant Graphics for Data Analysis. Springer-Verlag New York, 2016.
gridExtra	R package	Baptiste Auguie (2017). gridExtra: Miscellaneous Functions for "Grid" Graphics. R package version 2.3. https://CRAN.R-project.org/package=gridExtra
igraph	R package	Csardi G, Nepusz T: The igraph software package for complex network research, InterJournal, Complex Systems 1695. 2006. http://igraph.org
Matrix	R package	Bates DM, Maechler M. Package ‘Matrix’. R package version 1.2–12. 2017
pheatmap	R package	Raivo Kolde (2019). pheatmap: Pretty Heatmaps. R package version 1.0.12. https://CRAN.R-project.org/package=pheatmap
plotrix	R package	Lemon, J. (2006) Plotrix: a package in the red light district of R. R-News, 6(4): 8-12.
RColorBrewer	R package	Erich Neuwirth (2014). RColorBrewer: ColorBrewer Palettes. R package version 1.1-2. https://CRAN.R-project.org/package=RColorBrewer
scales	R package	Hadley Wickham (2018). scales: Scale Functions for Visualization. R package version 1.0.0. https://CRAN.R-project.org/package=scales
smacof	R package	Jan de Leeuw, Patrick Mair (2009). Multidimensional Scaling Using Majorization: SMACOF in R. Journal of Statistical Software, 31(3), 1-30. URL http://www.jstatsoft.org/v31/i03/
stats	R package	R Core Team (2018). R: A language and environment for statistical computing. R Foundation for Statistical Computing, Vienna, Austria. URL https://www.R-project.org/.
vegan	R package	Jari Oksanen, F. Guillaume Blanchet, Michael Friendly, Roeland Kindt, Pierre Legendre, Dan McGlinn, Peter R. Minchin, R. B. O'Hara, Gavin L. Simpson, Peter Solymos, M. Henry H. Stevens, Eduard Szoecs and Helene Wagner (2019). vegan: Community Ecology Package. R package version 2.5-4. https://CRAN.R-project.org/package=vegan
DESeq2	R package	Reference[16]
IPC::System::Simple	Perl module	Paul Fenwick
Bio::SeqIO	Perl module	Christopher Fields
Bio::Seq	Perl module	Christopher Fields
Text::Levenshtein::XS	Perl module	Nick Logan
Text::WagnerFischer	Perl module	Dree Mistrut
List::Util	Perl module	Graham Barr, Paul Evans
Statistics::Basic	Perl module	Paul Miller
Excel::Writer::XLSX	Perl module	John McNamara
Math::round	Perl module	Geoffrey Rommel

Supplementary Table 5: Information on the presented sequencing runs.

Sequencing run	Name	Description	Index	Reads from MiSeq	Processed data
1	Mock-a	Three independent measurements for the mock community	GGCTCTGA	10548818	Supplementary Data 1 & 6
	Mock-b		AGGCGAAG	4899060	
	Mock-c		GTACTGAC	4714685	
	M0-a	Three independent measurements for the control (M0)	CCTATCCT	2204110	Supplementary Data 1 & 6
	M0-b		TAATCTTA	1934004	
	M0-c		CAGGACGT	2461126	
	Spike-in-control-run1-type1	Four types of spike-in controls for BarBIQ sequencing	ACTGCATA	1673615	-
	Spike-in-control-run1-type2		TAGATCGC	1092643	-
	Spike-in-control-run1-type3		CTCTCTAT	621515	-
	Spike-in-control-run1-type4		GTAAGGAG	629907	-
2	CEa^{dist}1	Cells of three technical replicates (filtration) of the sample from the distal location in mouse CEa	CTCACATA	1687682	Supplementary Data 1 & 8
	CEa^{dist}2		ACATAGCG	1762873	
	CEa^{dist}3		AACAGGAA	1993315	
	CEa^{prox}1	Cells of three technical replicates (filtration) of the sample from the proximal location in mouse CEa	ATAGAGGC	1512069	
	CEa^{prox}2		AGGCGAAG	2220882	
	CEa^{prox}3		CAGGACGT	1537713	
	CEa^{prox}1-ecDNA	ecDNAs of three technical replicates (filtration) of the sample from the proximal location in mouse CEa	CCTATCCT	733321	
	CEa^{prox}2-ecDNA		TAATCTTA	804879	
	CEa^{prox}3-ecDNA		GTACTGAC	760419	
	CEa^{dist}-Unfiltered	Unfiltered sample from the distal location in mouse CEa	TATAGCCT	2102311	
	CEa^{prox}-Unfiltered	Unfiltered sample from the proximal location in mouse CEa	AGAATCAA	1617569	
	Control-Cell-1	Cells of a control (empty tube) for cell-samples in the CE2-nutrient group	TATGAGTA	290779	-
	Control-ecDNA-1	ecDNAs of a control (empty tube) for ecDNA-samples in the CE2-nutrient group	GCGAAGAT	292232	-
	Control-Unfiltered-1	Unfiltered control (empty tube) for unfiltered-samples in the CE2-nutrient group	GACTAACG	280919	-
	Spike-in-control-run2-type1	Four types of spike-in controls for BarBIQ sequencing	ACTGCATA	1252020	-
	Spike-in-control-run2-type2		TAGATCGC	850681	-
	Spike-in-control-run2-type3		CTCTCTAT	473765	-
	Spike-in-control-run2-type4		GTAAGGAG	477803	-
3	CEa^{dist}1-ecDNA	ecDNAs of three technical replicates (filtration) of a sample from the distal location in mouse CEa	ATAGAGGC	950135	Supplementary Data 1 & 8
	CEa^{dist}2-ecDNA		CCTATCCT	1137168	
	CEa^{dist}3-ecDNA		AGGCGAAG	1145204	
	CEb^{dist}	Cells from the distal location in mouse CEb	GGTGAAGG	2563902	
	CEb^{dist} -ecDNA	ecDNAs from the distal location in mouse CEb	CAGGACGT	1288690	
	CEb^{prox}	Cells from the proximal location in mouse CEb	GTACTGAC	2854221	
	CEb^{prox}-ecDNA	ecDNAs from the proximal location in mouse CEb	AGAATCAA	1325080	
	CEc^{dist}	Cells from the distal location in mouse CEc	CTCACATA	2710495	
	CEc^{dist} -ecDNA	ecDNAs from the distal location in mouse CEc	ACATAGCG	1243756	
	CEc^{prox}	Cells from the proximal location in mouse CEc	AACAGGAA	2900730	
	CEc^{prox}-ecDNA	ecDNAs from the proximal location in mouse CEc	TATAGCCT	1429968	
	Control-Cell-2	Cells of a control (empty tube) for cell-samples in the CE2-nutrient group	TATGAGTA	366369	-
	Control-ecDNA-2	ecDNAs of a control (empty tube) for ecDNA-samples in the CE2-nutrient group	GCGAAGAT	492617	-
	Control-Unfiltered-2	Unfiltered control (empty tube) for unfiltered-samples in the CE2-nutrient group	GACTAACG	403669	-
	Spike-in-control-run3-type1	Four types of spike-in controls for BarBIQ sequencing	ACTGCATA	1310438	-
	Spike-in-control-run3-type2		TAGATCGC	868954	-
	Spike-in-control-run3-type3		CTCTCTAT	491752	-
	Spike-in-control-run3-type4		GTAAGGAG	494660	-
	VDa^{dist}	Cells from the distal location in mouse VDa	TATAGCCT	1788062	

4	VDb^{prox}	Cells from the proximal location in mouse VDb	CCTATCCT	1240853	Supplementary Data 1 & 8
	VDd^{prox}	Cells from the proximal location in mouse VDd	AGGCGAAG	1866995	
	VSa^{prox}	Cells from the proximal location in mouse VSa	TAATCTTA	1981563	
	VSb^{dist}	Cells from the distal location in mouse VSb	CAGGACGT	1854914	
	VSc^{prox}	Cells from the proximal location in mouse VSc	GTACTGAC	1858975	
	VDa^{dist}-ecDNA	ecDNAs from the distal location in mouse VDa	CCCTTGTG	1697321	
	VDb^{prox}-ecDNA	ecDNAs from the proximal location in mouse VDb	AGAATCAA	1632665	
	VDd^{prox}-ecDNA	ecDNAs from the proximal location in mouse VDd	GACTAACG	1828847	
	VSa^{prox}-ecDNA	ecDNAs from the proximal location in mouse VSa	CTCACATA	1512792	
	VSb^{dist}-ecDNA	ecDNAs from the distal location in mouse VSb	AGCGCTAG	1446806	
	VSc^{prox}-ecDNA	ecDNAs from the proximal location in mouse VSc	GATATCGA	1584756	
	Control-Cell-3	Cell of a control (empty tube) for cell-samples in the VA group	GCGAAGAT	201724	-
	Control-ecDNA-3	ecDNAs of a control (empty tube) for ecDNA-samples in the VA group	CGCAGACG	116237	-
	Spike-in-control-run4-type1	Four types of spike-in controls for BarBIQ sequencing	ACTGCATA	1055180	-
	Spike-in-control-run4-type2		TAGATCGC	719167	-
	Spike-in-control-run4-type3		CTCTCTAT	401556	-
	Spike-in-control-run4-type4		GTAAGGAG	400115	-
5	VDb^{dist}	Cells from the distal location in mouse VDb	AGGTGCGT	1457049	Supplementary Data 1 & 8
	VDd^{dist}	Cells from the distal location in mouse VDd	GAACATAC	1450537	
	VSa^{dist}	Cells from the distal location in mouse VSa	ACATAGCG	1475927	
	VSb^{prox}	Cells from the proximal location in mouse VSb	GTGCGATA	1271124	
	VSc^{dist}	Cells from the distal location in mouse VSc	CCAACAGA	1387774	
	VDb^{dist}-ecDNA	ecDNAs from the distal location in mouse VDb	TATAACCT	1455102	
	VDd^{dist}-ecDNA	ecDNAs from the distal location in mouse VDd	AAGGATGA	1571182	
	VSa^{dist}-ecDNA	ecDNAs from the distal location in mouse VSa	TCGTGACC	1307141	
	VSb^{prox}-ecDNA	ecDNAs from the proximal location in mouse VSb	CTACAGTT	540265	
	VSc^{dist}-ecDNA	ecDNAs from the distal location in mouse VSc	ATATTCAC	1631871	
	VDc^{prox}	Cells from the proximal location in mouse VDc	ACTCTATG	1274898	
	VDc^{dist}	Cells from the distal location in mouse VDc	GTCTCGCA	1271665	
	VSd^{prox}	Cells from the proximal location in mouse VSd	AAGACGTC	1302496	
	VSd^{dist}	Cells from the distal location in mouse VSd	GGAGTACT	1401961	
	VDc^{prox}-ecDNA	ecDNAs from the proximal location in mouse VDc	GTTAATTG	1259332	
	VDc^{dist}-ecDNA	ecDNAs from the distal location in mouse VDc	AACCGCGG	1344651	
	VSd^{prox}-ecDNA	ecDNAs from the proximal location in mouse VSd	CCAAGTCC	1093845	
	VSd^{dist}-ecDNA	ecDNAs from the distal location in mouse VSd	TTGGACTT	1348773	
	VDa^{prox}	Cells from the proximal location in mouse VDa	TATGAGTA	2007618	
	VDa^{prox}-ecDNA	ecDNAs from the proximal location in mouse VDa	CGCGGTTC	1892005	
	Control-Cell-4	Cells of a control (empty tube) for cell-samples in the VA group	TTGGTGAG	109563	-
	Control-ecDNA-4	ecDNAs of a control (empty tube) for ecDNA-samples in the VA group	GCGCCTGT	93397	-

	Control-Cell-5	Cells of a control (empty tube) for cell-samples in the VA group	ACCGGCCA	100972	-
	Control-ecDNA-5	ecDNAs of a control (empty tube) for ecDNA-samples in the VA group	CAGTGGAT	99011	-
	Spike-in-control-run5-type1	Four types of spike-in controls for BarBIQ sequencing	ACTGCATA	980501	-
	Spike-in-control-run5-type2		TAGATCGC	666034	-
	Spike-in-control-run5-type3		CTCTCTAT	372136	-
	Spike-in-control-run5-type4		GTAAGGAG	368838	-

Supplementary References

1. Kozich, J. J., Westcott, S. L., Baxter, N. T., Highlander, S. K. & Schloss, P. D. Development of a Dual-Index Sequencing Strategy and Curation Pipeline for Analyzing Amplicon Sequence Data on the MiSeq Illumina Sequencing Platform. *Appl. Environ. Microbiol.* **79**, 5112–5120 (2013).
2. Ogawa, T., Kryukov, K., Imanishi, T. & Shiroguchi, K. The efficacy and further functional advantages of random-base molecular barcodes for absolute and digital quantification of nucleic acid molecules. *Sci. Rep.* **7**, 13576 (2017).
3. Bolger, A. M., Lohse, M. & Usadel, B. Trimmomatic: A flexible trimmer for Illumina sequence data. *Bioinformatics* **30**, 2114–2120 (2014).
4. Tan, G., Opitz, L., Schlapbach, R. & Rehrauer, H. Long fragments achieve lower base quality in Illumina paired-end sequencing. *Sci. Rep.* **9**, 2856 (2019).
5. Quast, C. *et al.* The SILVA ribosomal RNA gene database project: improved data processing and web-based tools. *Nucleic Acids Res.* **41**, D590–D596 (2013).
6. Mysara, M., Saeys, Y., Leys, N., Raes, J. & Monsieurs, P. CATCh, an ensemble classifier for chimera detection in 16S rRNA sequencing studies. *Appl. Environ. Microbiol.* **81**, 1573–1584 (2015).
7. Borgström, E. *et al.* Phasing of single DNA molecules by massively parallel barcoding. *Nat. Commun.* **6**, 7173 (2015).
8. Sheth, R. U. *et al.* Spatial metagenomic characterization of microbial biogeography in the gut. *Nat. Biotechnol.* **37**, 877–883 (2019).
9. Ratcliff, W. C., Denison, R. F., Borrello, M. & Travisano, M. Experimental evolution of multicellularity. *Proc. Natl. Acad. Sci. U. S. A.* **109**, 1595–1600 (2012).
10. Claessen, D., Rozen, D. E., Kuipers, O. P., Søgaard-Andersen, L. & Van Wezel, G. P. Bacterial solutions to multicellularity: A tale of biofilms, filaments and fruiting bodies. *Nat. Rev. Microbiol.* **12**, 115–124 (2014).
11. Schneider, C. A., Rasband, W. S. & Eliceiri, K. W. NIH Image to ImageJ: 25 years of image analysis. *Nat. Methods* **9**, 671–675 (2012).
12. Potapov, V. & Ong, J. L. Examining sources of error in PCR by single-molecule sequencing. *PLoS One* **12**, 1–19 (2017).

13. Stoddard, S. F., Smith, B. J., Hein, R., Roller, B. R. K. & Schmidt, T. M. rrnDB: Improved tools for interpreting rRNA gene abundance in bacteria and archaea and a new foundation for future development. *Nucleic Acids Res.* **43**, D593–D598 (2015).

14. Ricardo, P. C., Françoso, E. & Arias, M. C. Fidelity of DNA polymerases in the detection of intraindividual variation of mitochondrial DNA. *Mitochondrial DNA Part B Resour.* **5**, 108–112 (2020).

15. Wilk, M. B. & Gnanadesikan, R. Probability plotting methods for the analysis for the analysis of data. *Biometrika* **55**, 1–17 (1968).

16. Love, M. I., Huber, W. & Anders, S. Moderated estimation of fold change and dispersion for RNA-seq data with DESeq2. *Genome Biol.* **15**, 1–21 (2014).
