# Supplementary material for: High-throughput identification and quantification of single bacterial cells in the microbiota
Source: Nat Commun. 2022 Feb 22;13:863. doi: 10.1038/s41467-022-28426-1 (PMC8863893; doi:10.1038/s41467-022-28426-1)
Supplement: Supplementary file 2 — Description of Additional Supplementary Files [file 41467_2022_28426_MOESM2_ESM.docx]

**Description of Additional Supplementary Files**

File Name: Supplementary Data 1
Description: BarBIQ-identified sequences (Bar sequences).

File Name: Supplementary Data 2
Description: Sanger sequencing-identified 16S rRNA sequences (San sequences).

File Name: Supplementary Data 3

Description: Sequences of amplicon sequence variants (ASVs).

File Name: Supplementary Data 4

Description: Representative sequences of operational taxonomic units (OTU-RepSeqs).

File Name: Supplementary Data 5

Description: Sequencing-determined abundances of amplicon sequence variants (ASVs).

File Name: Supplementary Data 6

Description: cOTU abundances of the mock community and the control M0.

File Name: Supplementary Data 7

Description: Sequencing-determined abundances of operational taxonomic units (OTUs).

File Name: Supplementary Data 8

Description: cOTU abundances of cecal samples.

File Name: Source Data
Description: Source data for figures in main text and Supplementary Information.
