# Supplementary material for: High-throughput identification and quantification of single bacterial cells in the microbiota
Source: Nat Commun. 2022 Feb 22;13:863. doi: 10.1038/s41467-022-28426-1 (PMC8863893; doi:10.1038/s41467-022-28426-1)
Supplement: Supplementary file 11 — Reporting Summary [file 41467_2022_28426_MOESM11_ESM.pdf]

nature research

Corresponding author(s): Katsuyuki Shiroguchi

Last updated by author(s): Jan 17, 2022

Reporting Summary

Nature Research wishes to improve the reproducibility of the work that we publish. This form provides structure for consistency and transparency in reporting. For further information on Nature Research policies, see our Editorial Policies and the Editorial Policy Checklist.

Statistics

For all statistical analyses, confirm that the following items are present in the figure legend, table legend, main text, or Methods section.

n/a	Confirmed	
[ ]	[x]	The exact sample size (*n*) for each experimental group/condition, given as a discrete number and unit of measurement
[ ]	[x]	A statement on whether measurements were taken from distinct samples or whether the same sample was measured repeatedly
[ ]	[x]	The statistical test(s) used AND whether they are one- or two-sided<br>*Only common tests should be described solely by name; describe more complex techniques in the Methods section.*
[x]	[ ]	A description of all covariates tested
[ ]	[x]	A description of any assumptions or corrections, such as tests of normality and adjustment for multiple comparisons
[ ]	[x]	A full description of the statistical parameters including central tendency (e.g. means) or other basic estimates (e.g. regression coefficient) AND variation (e.g. standard deviation) or associated estimates of uncertainty (e.g. confidence intervals)
[ ]	[x]	For null hypothesis testing, the test statistic (e.g. *F*, *t*, *r*) with confidence intervals, effect sizes, degrees of freedom and *P* value noted<br>*Give P values as exact values whenever suitable.*
[x]	[ ]	For Bayesian analysis, information on the choice of priors and Markov chain Monte Carlo settings
[x]	[ ]	For hierarchical and complex designs, identification of the appropriate level for tests and full reporting of outcomes
[ ]	[x]	Estimates of effect sizes (e.g. Cohen's *d*, Pearson's *r*), indicating how they were calculated

Our web collection on statistics for biologists contains articles on many of the points above.

Software and code

Policy information about availability of computer code

Data collection	Illumina Miseq, QX200 Droplet Digital PCR System.
Data analysis	Perl (version 5.22.1), R (version 3.5.1 or 4.1.1), JupyterLab (version 0.34.9), nucleotide-sequence-clusterizer (version 0.0.7), bwa (version 0.7.15), Mothur (version 1.35.1), Trimmomatic (version 0.38), DADA2 (Version v1.20.0), RDP Classifier (with training set 16), NCBI BLAST (version 2.7.1), Fastq-tools-0.8 (version 0.8--1), DESeq2 (Version 1.32.0). The BarBIQ pipeline is available at github (https://github.com/Shiroguchi-Lab/BarBIQ).

For manuscripts utilizing custom algorithms or software that are central to the research but not yet described in published literature, software must be made available to editors and reviewers. We strongly encourage code deposition in a community repository (e.g. GitHub). See the Nature Research guidelines for submitting code & software for further information.

Data

Policy information about availability of data

All manuscripts must include a data availability statement. This statement should provide the following information, where applicable:

- Accession codes, unique identifiers, or web links for publicly available datasets
- A list of figures that have associated raw data
- A description of any restrictions on data availability

The sequencing data are available from the NCBI Sequence Read Archive under accessions PRJNA639639[https://www.ncbi.nlm.nih.gov/bioproject/?term=PRJNA639639], PRJNA639647[https://www.ncbi.nlm.nih.gov/bioproject/?term=PRJNA639647], PRJNA780331[https://www.ncbi.nlm.nih.gov/bioproject/?term=PRJNA780331], and PRJNA780361[https://www.ncbi.nlm.nih.gov/bioproject/?term=PRJNA780361]. The databases, GreenGenes (Release 13_5)[https://greengenes.secondgenome.com/], Ribosomal Database Project (Release 11.5)[https://rdp.cme.msu.edu], Silva (Release 123.1 and 138; SSU Ref NR 99)[https://www.arb-silva.de], and rrnDB (version 5.7)[https://rrndb.umms.med.umich.edu] are available online. Source data are provided with this paper.

Field-specific reporting

Please select the one below that is the best fit for your research. If you are not sure, read the appropriate sections before making your selection.

- [x] Life sciences
- [ ] Behavioural & social sciences
- [ ] Ecological, evolutionary & environmental sciences

For a reference copy of the document with all sections, see nature.com/documents/nr-reporting-summary-flat.pdf

Life sciences study design

All studies must disclose on these points even when the disclosure is negative.

Sample size	To reduce to an absolute minimum based on ethical guidelines, but to statistically compare individuals for our research purpose (sample size was not determined by a statistic method since the sample size was not large), we used 19 mice in total (6 mice were used in each of two groups for the vitamin A study, others were used for technical evaluation).
Data exclusions	No data were excluded.
Replication	All experiments were repeated twice or more (exact number of replications for each experiment is shown in the manuscript).
Randomization	We used random allocation of mice for the vitamin A-dependent experiments which is described in the Methods.
Blinding	The random allocation in our experiment was not blinded since all mice were apparently indistinguishable by human eyes.

Reporting for specific materials, systems and methods

We require information from authors about some types of materials, experimental systems and methods used in many studies. Here, indicate whether each material, system or method listed is relevant to your study. If you are not sure if a list item applies to your research, read the appropriate section before selecting a response.

Materials & experimental systems

n/a	Involved in the study	
x		Antibodies
x		Eukaryotic cell lines
x		Palaeontology and archaeology
	x	Animals and other organisms
x		Human research participants
x		Clinical data
x		Dual use research of concern

Methods

n/a	Involved in the study	
x		ChIP-seq
x		Flow cytometry
x		MRI-based neuroimaging

Animals and other organisms

Policy information about studies involving animals; ARRIVE guidelines recommended for reporting animal research

Laboratory animals	Six-week-old, eight-week-old, or nine-week-old C57BL/6J male or C57BL/6N female mice.
Wild animals	The study did not involve wild animals.
Field-collected samples	The study did not involve samples collected from field.
Ethics oversight	All mouse procedures were performed in accordance with the ethical guidelines of the institute under the protocols approved by the Institutional Animal Care and Use Committee of RIKEN or the Animal Experimentation Committee of the Institute for Frontier Life and Medical Sciences, Kyoto University.

Note that full information on the approval of the study protocol must also be provided in the manuscript.
